# Indole C6 Functionalization of Tryprostatin B Using Prenyltransferase CdpNPT

**DOI:** 10.3390/catal10111247

**Published:** 2020-10-28

**Authors:** Eric D. Gardner, Dustin A. Dimas, Matthew C. Finneran, Sara M. Brown, Anthony W. Burgett, Shanteri Singh

**Affiliations:** 1 Department of Chemistry and Biochemistry, University of Oklahoma, Norman, OK 73019, USA; 2 Department of Pharmaceutical Sciences, University of Oklahoma, Oklahoma City, OK 73117, USA; † Neuroscience Graduate Program, University of Michigan Medical School, Ann Arbor, MI 48109, USA.

**Keywords:** biocatalysts, chemoenzymatic synthesis, prenyltransferase, tryprostatin, late-stage functionalization

## Abstract

Tryprostatin A and B are prenylated, tryptophan-containing, diketopiperazine natural products, displaying cytotoxic activity through different mechanisms of action. The presence of the 6-methoxy substituent on the indole moiety of tryprostatin A was shown to be essential for the dual inhibition of topoisomerase II and tubulin polymerization. However, the inability to perform late-stage modification of the indole ring has limited the structure–activity relationship studies of this class of natural products. Herein, we describe an efficient chemoenzymatic approach for the late-stage modification of tryprostatin B using a cyclic dipeptide *N*-prenyltransferase (CdpNPT) from *Aspergillus fumigatus*, which generates novel analogs functionalized with allylic, benzylic, heterocyclic, and diene moieties. Notably, this biocatalytic functionalizational study revealed high selectivity for the indole C6 position. Seven of the 11 structurally characterized analogs were exclusively C6-alkylated, and the remaining four contained predominant C6-regioisomers. Of the 24 accepted substrates, 10 provided >50% conversion and eight provided 20–50% conversion, with the remaining six giving <20% conversion under standard conditions. This study demonstrates that prenyltransferase-based late-stage diversification enables direct access to previously inaccessible natural product analogs.

## 1. Introduction

**Figure 1.**
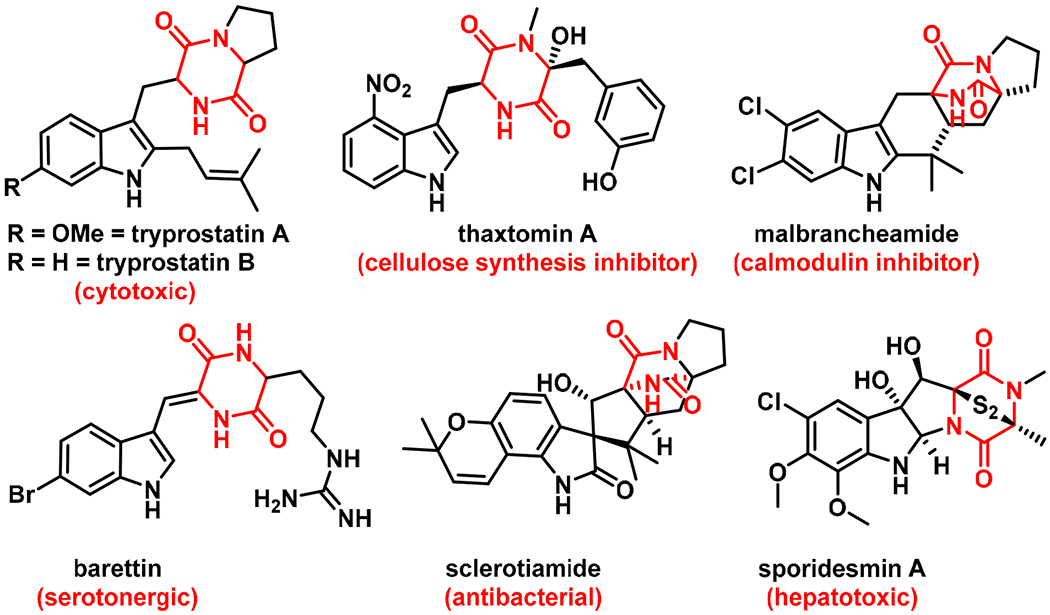
Representative bioactive indole diketopiperazines with diverse activities.

Indole diketopiperazines (DKPs) represent a large class of biologically active natural products (NPs), isolated predominately from fungi, [1,2] with members exhibiting a wide variety of biological activities such as anticancer (tryprostatin, malbrancheamide) [3], herbicidal (thaxtomin A) [4], serotonergic (barettin) [5], antimicrobial (sclerotiamide) [6], antioxidant (neoechinulin A) [7], and immunomodulatory (cristatin A) [8] activities (Figure 1). These DKPs are biosynthesized through the cyclization of tryptophan with other amino acids (proline, leucine, histidine, phenylalanine, etc.), followed by modification by enzymes such as aromatic prenyltransferases (PTs), cytochrome P450s, and halogenases that build the final NP scaffold. Among the DKPs, tryprostatins (TPS-A and TPS-B, Figure 1) are Trp-Pro cyclic dipeptides with an indole C2-prenyl group, originally isolated from *Aspergillus fumigatus* BM 939 [9]. The distinguishing feature between the two compounds is the presence (TPS-A) or absence (TPS-B) of a methoxy substitution at the indole C6-position. Both compounds are known to display anticancer activities as a result of the indole C2 prenyl group, which has been linked to their inhibition of topoisomerase II [10]. However, TPS-A has also been shown to inhibit tubulin polymerization and breast cancer resistance protein (BCRP, ABCG2) [11], and an effective inhibition of BCRP by TPS-A has been shown to restore the efficacy of clinically used chemotherapeutics when tested on BCRP+ breast cancer cell lines [12]. The proposed mechanism of action (MOA), where TPS-A induces tubulin inhibition without directly binding to tubulin, was found to be distinct from the typical tubulin inhibitors such as paclitaxel or vinblastine [9]. Thus, the 6-methoxy substituent of TPS-A appears to enable binding to a new range of biological targets, highlighting the importance of the C6 indole functionalization of the TPS scaffold.

However, the developed synthetic methods to modify indole DKPs thus far have been largely focused on indole functionalization at the C2, C3, and N1 positions [11]. Among the limited methods to functionalize the benzene portion of indole, functionalization at C4 and C7 typically rely on coordination of the catalyst to specific directing groups installed on either C3 or N1, respectively [13–15]. However, this method suffers from low functional group tolerance and a limited substrate scope [14–22]. The few described methods for C5 and C6 indole functionalization provide limited functional group tolerance [19,23]. Recently, Baran et al. developed a remarkable example of iridium-catalyzed ligand-controlled selective C6 tryptophan borylation;however, even this groundbreaking directing group-free method requires protecting groups and is unable to tolerate tryptophan containing cyclic dipeptides [24]. Our aim is to address this urgent need to develop methodology capable of diversifying the unreactive positions of the indole ring of these complex natural product scaffolds in order to access a massive untapped chemical space.

Within this context, aromatic PTs show great promise as biocatalysts both in a general sense and more specifically as tools for indole modification. They demonstrate broad substrate specificities for both alkyl-donors and acceptors, transferring a variety of allylic, benzylic, dienyl, and heterocyclic groups from their corresponding pyrophosphates on to both native and non-native acceptors [25–31]. Specific PTs have also been demonstrated to possess practical utility beyond generalized substrate promiscuity. For example, the naphterpin PT (NphB) has been shown to N-alkylate the antibacterial sulfabenzamide [26], and the indole PT cyclic dipeptide *N*-prenyltransferase (CdpNPT) has been demonstrated to modify the indole N1, C2, C3, C5, and C6 positions of the macrocyclic peptide antibiotic daptomycin, resulting in alkylated analogs with potency against daptomycin-resistant bacterial strains [28,32]. Thus, the increased activity of the novel daptomycin analogs has provided strong evidence of the PTs’ utility in drug design, while the alkylation of the indole C5 and C6 positions by CdpNPT demonstrates synthetic utility in modifying chemically inert benzenoid positions on indole rings.

Based on these findings and the demonstrated importance of indole C6 functionalization on the TPS scaffold, we hypothesized that CdpNPT could be used to generate benzenoid-functionalized TPS-analogs from TPS-B. Herein, we report the chemoenzymatic synthesis of TPS-B analogs using a library of synthetic alkyl donors, which revealed CdpNPT’s ability to transfer several allylic, dienyl, benzylic, and heterocylic groups predominantly onto C6 of TPS-B with high regioselectivity. Furthermore, the new TPS analogs were assayed for cytotoxic activity against the human leukemia cancer cell line K562. While the modification of the indole C6 position with alkyl, dienyl, and benzylic groups did not substantially change the cytotoxicity activity of tryprostatin analogs, these provided additional SAR information for indole DKPs. This study demonstrates the utility of aromatic PTs as a tool for the functionalization of inert positions of indole DKPs, providing access to previously inaccessible natural product analogs.

## 2. Results and Discussion

### 2.1. Design of Alkyl Pyrophosphate Donors

**Figure 2.**
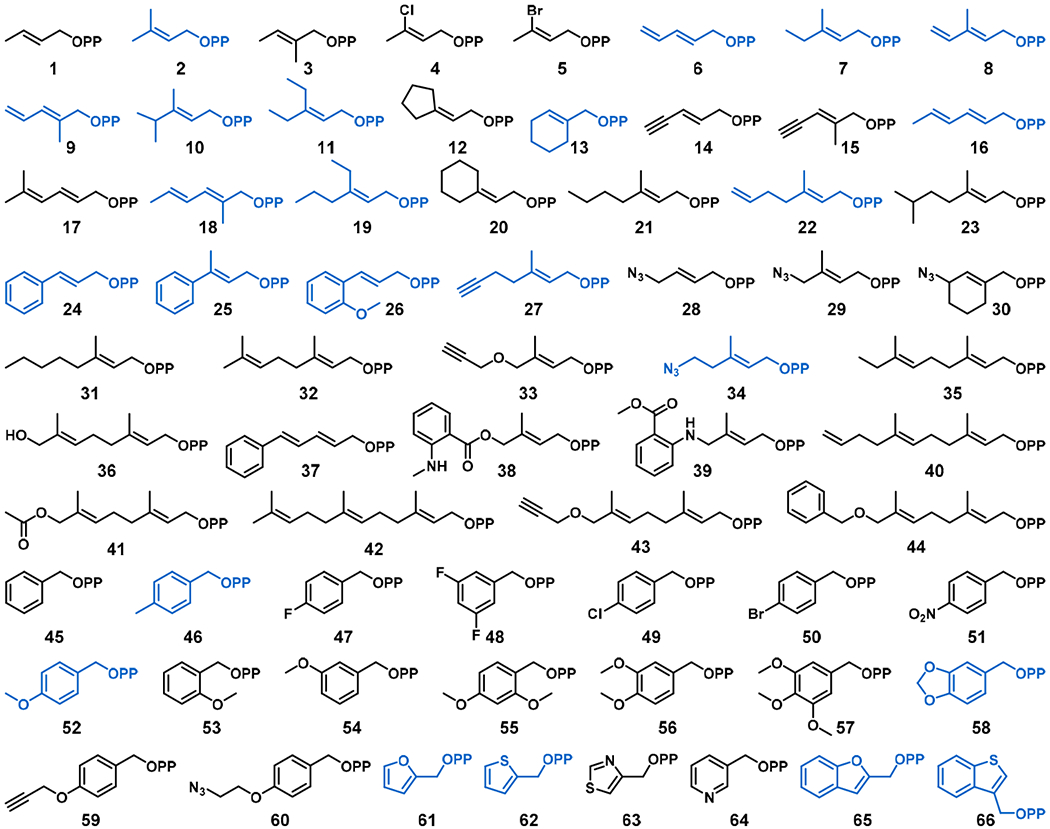
Library of alkyl-pyrophosphate (alkyl-PP) analogs used in this study to probe the donor substrate scope of cyclic dipeptide *N*-prenyltransferase (CdpNPT) for tryprostatin B (TPS-B) modification. Alkyl-PP donors accepted by CdpNPT are colored in blue.

The alkyl-pyrophosphate (alkyl-PP) donor library developed for the present study is shown in Figure 2, and each compound was designed to represent steric and electronic configuration within the established requirements of PT donor substrates. To undergo successful electrophilic aromatic substitution in a PT reaction, the pyrophosphate (OPP) of the alkyl-PP donor must dissociate to generate a stabilized carbocation [33]. Benzylic and allylic carbocations are stabilized through resonance and inductive effects to promote OPP dissociation, with electron-donating substituents further increasing the reactivity [34–36]. As such, both allylic (1–44) and benzylic (45–60) analogs were well-represented in the final library. Within the allylic alkyl-PPs, structural and electronic differences were established through variations in carbon chain length, branching patterns, and the inclusion of heteroatoms such as nitrogen, oxygen, chlorine, and bromine. The benzylic analogs were distinguished through differences in the location, extent, and electron donation/withdrawal potential of various substituents on the benzene ring. Additionally, a subset of the alkyl-PPs were heterocyclic in nature (61–66), maintaining the required allylic configuration while varying in the identity of the heteroatom, the size of the ring system, and the location of allylic attachment. Then, the library of 66 synthetic alkyl-PP donors (Figure 2) [25–28] was used to determine the substrate scope of CdpNPT catalyzed TPS-B functionalization of TPS-B.

### 2.2. Donor Library Screening

Standard uniform assay conditions (1.5 mM alkyl-PP analog, 1 mM TPS-B, 10 μM CdpNPT, 25 mM Tris, 5 mM CaCl_2_, pH 7.8,18 h at 35 °C) were adopted to facilitate an initial assessment of CdpNPT’s donor scope with TPS-B as the acceptor. Once reactions were complete, the protein was precipitated with cold methanol and removed by centrifugation. Then, the clarified reactions were analyzed using a reverse phase high-pressure liquid chromatography (RP-HPLC) endpoint assay, and the production of alkyl-TPS derivatives was determined by the difference in retention between TPS-B and alkyl-TPS. All positive reactions underwent subsequent confirmation by high-resolution electrospray ionization mass spectra (ESI-HRMS) (see Supplementary Materials, Figure S1 and Tables S1 and S2).

**Figure 3.**
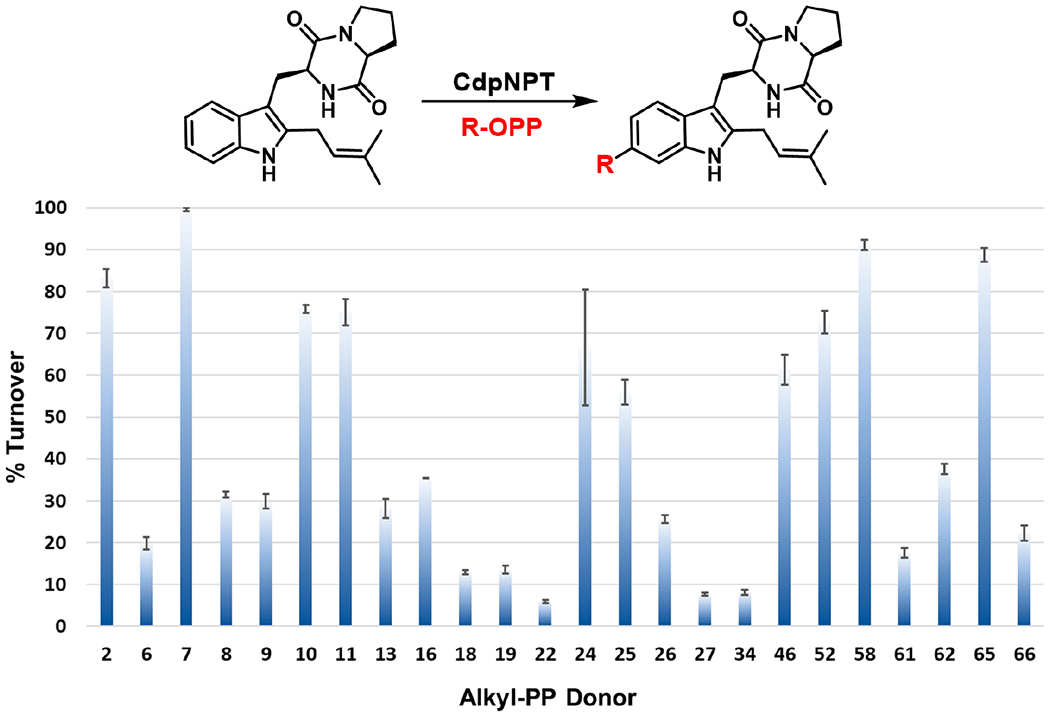
Alkyl donor profile of analytical-scale CdpNPT-catalyzed reactions with TPS-B. The average percent conversion of all positive reactions is shown with the associated standard deviations (n = 2) as determined by RP-HPLC at 280 nm (see Supporting Information, Figure S1). Each reaction was carried out in a 20 μL volume and contained 1.5 mM alkyl-PP, 1 mM TPS-B, and 10 μM purified CdpNPT in a reaction buffer (25 mM Tris pH 7.8, and 5 mM CaCl_2_) incubated at 35 °C for 18 h. No product formation was observed in the absence of CdpNPT or alkyl-PP.

The initial assessment of CdpNPT catalyzed reactions revealed 24 out of the 66 screened alkyl-PP donors produced new products in the presence of CdpNPT and TPS-B (Figure 3). Ten of these positive reactions showed >50% conversion (**7, 24, 58, 65, 2, 10, 11, 52, 46, 25**), eight displayed moderate conversion (20–50%; **62, 16, 8, 9, 13, 26, 66, 6**) and an additional six reactions produced low conversion (<20%; **61, 19, 18, 34, 27, 22**).

### 2.3. Allylic Donors

**Figure 4.**
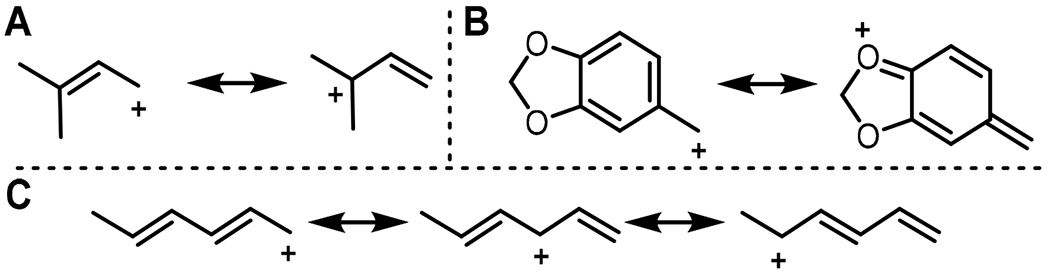
Depiction of the resonance stabilization of representative alkyl-PP donors (**A**) **2,** (**B**) **58,** and (**C**) **16**.

Among the allylic alkyl-PP analogs, DMAPP (**2**) represents the natural donor for CdpNPT-catalyzed reactions, in which it forms a highly stabilized carbocation intermediate through the delocalization of its positive charge between primary and tertiary carbons (Figure 4A). Thus, alkyl-PP analogs with similar size and planarity to **2** typically gave comparable conversion (**7, 10, 11**). However, longer-chain analogs **19**, **22**, **27**, and **34** were taken less efficiently, while the 10-carbon geranyl pyrophosphate (**32**) and the 15-carbon farnesyl pyrophosphate (**42**) generated no product. Therefore, the yields of product formation of the CdpNPT catalyzed reactions with TPS-B in general decreased with the increase in the chain length of the alkyl-PP analog. Furthermore, the conjugated aromatic donors **24** and **25** were well accepted by CdpNPT, which was likely due to the formation of a delocalized carbocation and the overall planarity of the substrate. This high turnover is notable considering **24** and **25** are seven carbons long, which is the maximum tolerated size observed in the allylic donors. Thus, these results support the hypothesis that while the capacity of a donor to delocalize the carbocation intermediate is critical for their efficient utility, steric constraints within the active site play a major role in their acceptability by CdpNPT.

### 2.4. Benzylic and Heterocyclic Donors

The carbocation stabilization on benzylic pyrophosphates takes place as a combination of resonance and inductive effects. While CdpNPT did not accept the *ortho*- and *meta*-substituted benzyl pyrophosphates (**48, 53, 54**), it was very efficient in transferring benzylic donors featuring electron donating groups at the *para* position, including the methyl (**46**) and methoxy (**52**) groups. Notably, the unsubstituted benzyl pyrophosphate **45** did not generate a detectable product, suggesting that carbocation stabilizing substituents are necessary for CdpNPT to transfer benzylic moieties. Even more interesting was piperonyl pyrophosphate (**58**), which produced the highest product yield among the aromatic (benzylic or heterocyclic) donors. This is even more surprising given that the di-methoxy analog **56** showed no discernable turnover. This phenomenon is likely due to steric differences between the two moieties. In both cases, the oxygen atom *para* to the benzyl tail provides similar resonance stabilization to the resulting carbocations (Figure 4B). Therefore, the drastic difference in activity likely results from the conformational constraints of the benzodioxole ring reducing the entropic barrier for binding when compared to the more flexible and bulkier methoxy groups of **56**. These observations again highlight the potential steric limitations of the binding pocket. Several heterocyclic analogs were also successfully transferred by CdpNPT such as furan (**61**), benzofuran (**65**), thiophene (**62**), and benzothiophene (**66**). The heteroatoms present in these donors can donate their lone pair of electrons to delocalize and thereby stabilize the positive charge more effectively than a simple benzyl carbocation.

### 2.5. Other Functionalized Donors

The synthetic utility of PT catalyzed late-stage functionalization would be greatly enhanced if these enzymes efficiently transferred donors containing polar functional groups or other synthetic handles such as azides or alkynes. Azide or alkyne-containing compounds are particularly desirable because they enable click chemistry-based derivatization. Of the alkyne- and azide-substituted donors, only **27** and **34** were accepted by CdpNPT, both of which showed poor conversions (<10%, Figure 3). This was likely the result of their length, as well as the unnatural linear rigidity imparted by the alkyne or azide group. The intolerance of these rigid linear groups is further noted in the shorter chain alkyne and azide donors, **14**, **15**, **28**, and **29**, which generated no detectable product under the standard reaction conditions. The low conversion exclusively shown in intermediate length alkyne/azide examples (**27**, **34**) is likely due to the increased conformational flexibility provided by the additional methylene. The azide donor **34** is the only example of a donor exceeding seven atoms in the linear chain that generated products, and also the only accepted donor containing a polar functional group. These exceptions could possibly be a result of hydrogen bonding interactions occurring at the solvent-exposed edge of the donor binding pocket.

The diene-containing donors **6**, **8**, **9**, and **16** were accepted by CdpNPT, although a lower degree of regioselectivity was observed for **6**, **8**, and **9**. This decrease in regioselectivity could be a result of the multiple carbocation resonance forms and increased conformational flexibility compared to the other donor classes (Figure 4C). Overall, this screening study revealed a high degree of donor promiscuity for CdpNPT. HPLC analysis revealed that the production of a single product was observed in 16 (**10, 11, 13, 16, 19, 22, 24, 25, 26, 27, 34, 46, 52, 58, 65, 66**) of the 24 accepted substrates.

### 2.6. Scale-Up and Characterization of CdpNPT Reaction Products

**Figure 5.**
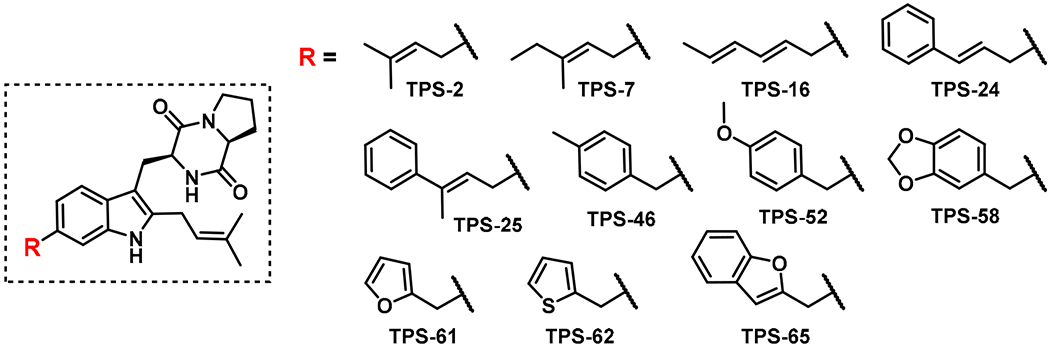
NMR-characterized structures of alkyl-TPS-B analogs, where the number following TPS represents the corresponding alkyl-PP used in the reaction. Alkyl-PP analogs **2,** **7,** **61,** and **62** produced 10–20% C-5 alkylated product. The regioisomers for **TPS-2**, **TPS-7**, and **TPS-61** were separated by semi-preparative RP-HPLC; however, **TPS-62** remained inseparable. (See Supplementary Materials).

To assess the regiospecificity of CdpNPT catalyzed alkyl-transfer, 11 representative reactions displaying structural diversity and high product yield in the analytical scale reactions (**2, 7, 16, 24, 25, 46, 52, 58, 61, 62, 65**) were scaled up at 10–15 μmol scale using standardized conditions (Figure 5). The resulting alkyl-TPS-B analogs were first extracted using ethyl acetate and then purified via RP-HPLC. Product formation was confirmed via HRMS (Supplementary Materials Table S2), and the regiospecificity was determined via ^1^H and 2D nuclear magnetic resonance (NMR) spectroscopy experiments acquired in CDCl_3_. Diagnostic ^1^H peaks for indole substitution patterns are well known in the literature, and these spectra were useful for the initial determination of each reaction’s regiospecificity. Typically, H4 is the most downfield indole CH signal at 7.4 ppm. For C6 alkylated products, the H4 peak appears as a doublet centered at this chemical shift, but C5 substituted products feature an H4 singlet shifted upfield due to the electron donating substituent (Supplementary Materials page S16). COSY, HSQC, and HMBC correlations were used to unequivocally verify the position of indole alkylation (Supplementary Materials Figure S3), and our analyses revealed the dominant products to be indole C6 alkylated in every case, with the occasional minor product being the C5 regioisomer (**2, 7, 61, 62**) as 10–20% of the reaction products (Supplementary Materials Table S3 and Figure S3). The donors that produced a mixture of regioisomers were **2**, **7**, the conjugated dienes (**6**, **8**, **9**, **18**), and the five-membered heterocycles (**61**, **62**). The common features between the less regioselective analogs is their small size and/or flexibility. The thiophene (**62**) and furan (**61**) pyrophosphates could in theory bind in two different orientations, with the heteroatom facing either side of the binding pocket, due to the small size and planarity of the donor substrate. These two competing binding conformations explain the observed decrease of regiospecificity, generating the C5 alkylated side product from the less favored conformation. Additionally, the smaller carbocations would have a greater ability to rotate before alkylation, decreasing the overall regiospecificity of their respective reactions. The larger, less flexible alkyl donors (**10, 11, 13, 19, 24, 27, 34**) all produced a single product likely due to the conformational constraints within the binding pocket. Based on the NMR confirmed products, all benzylic and cinnamyl donors (**25, 26, 46, 52, 58, 65, 66**) produced exclusively C6 alkylated products (NMR spectra and assignments available in the Supplementary Materials). Future studies to increase enzyme stability and enable enzyme reusability would enhance the practical utility and scalability of PT-based late-stage modification. Enzyme immobilization, covalent modification, and genetic engineering methods are currently being explored in order to address the limitations of this method [37–40].

### 2.7. Cytotoxicity Studies

A structurally diverse set of scaled up alkyl-TPS analogs with allylic, dienyl, benzylic, and heterobenzylic substituents (products of alkyl-PPs **2, 24, 25, 46, 52, 58, 61, 62, 65**) were tested for cytotoxicity against human leukemia K562 cells. (Table 1, Supplementary Materials Figure S2) Surprisingly, most of the alkylated analogs had similar potency to the parent compound. This remarkable tolerance of bulky substituents indicates that the diketopiperazine is perhaps the most dominant pharmacophore responsible for the observed cytotoxicity.

## 3. Materials and Methods

### 3.1. General Materials

Unless otherwise stated, all chemicals and reagents were purchased from Sigma-Aldrich (St. Louis, MO, USA), Acros (New Jersey, NJ, USA), Alfa-Aesar (Ward Hill, MA, USA), Ambeed (Arlington Heights, IL, USA), or AK Scientific (Union City, CA, USA) and were of reagent grade or better. The PD-10 column and Ni-NTA columns used for protein purification were purchased from GE Healthcare (Piscataway, NJ, USA).

### 3.2. General Methods

All synthetic reactions were conducted in oven-dried glassware under a nitrogen atmosphere with anhydrous solvents, unless otherwise noted. Flash column chromatography was performed with silica gel (SiliCycle Inc, Quebec City, Canada, P60, particle size 40–63 μm). HPLC was conducted using an Agilent 1220 system equipped with a diode array detector.

### 3.3. In Vitro CdpNPT Assay

The recombinant CdpNPT was overproduced in *Escherichia Coli* BL21(DE3) cells transformed with the codon-optimized synthetic CdpNPT gene in a pET28a vector. The resulting N-terminal His_6_–fusion protein was purified to homogeneity via Ni-NTA affinity chromatography, as previously reported [32]. Analytical scale in vitro CdpNPT reactions were conducted on a 20 μL scale containing 10 μM CdpNPT, 1 mM TPS-B, 1.5 mM alkyl-PP donor, 25 mM Tris pH 7.8, and 5 mM CaCl_2_ and were incubated at 35 °C for 18 h. Reactions were quenched by the addition of 40 μL cold methanol followed by centrifugation (9000× *g* for 30 min) to remove precipitated protein. Then, product formation was analyzed via RP-HPLC using Method A. Negative controls lacking enzyme or donor were included in the screening, and the percent conversion for each reaction was calculated using the peak integration at 280 nm. Specifically, the integrated total product areas were divided by the sum of the areas of integrated products and remaining substrate.

### 3.4. Enzymatic Scale-Up Reactions

All scale-up reactions were performed on a 10 μM or 15 μM scale in a 50 mL falcon tube containing 1 mM TPS-B, 1.5 mM alkyl-PP donor, 25 mM Tris, and 5 mM CaCl_2_ at pH 7.8. TPS-B was added as a 10 mM solution in DMSO to give a final DMSO content of 10%. CdpNPT was added for an initial concentration of 10 μM. Reaction mixtures were incubated at 35 °C, and progress was monitored via RP-HPLC using HPLC Method A. When low product formation was observed, additional enzyme was added, and incubation was continued for another 8–10 h. Once completed, reaction products were extracted four times with 15 mL volumes of ethyl acetate, which were then combined and concentrated in vacuo. Individual products were purified from the concentrates using semi-preparative RP-HPLC with HPLC (Method B).

### 3.5. Determination of Structures

The high-resolution mass spectrometry (HRMS) confirmation of products was conducted on an Agilent 6545-QTOF W/ 1290 HPLC mass spectrometer at the University of Oklahoma Department of Chemistry and Biochemistry. NMR spectra were obtained on a Varian VNMRS 500 MHz or a Varian INOVA 600 MHz instrument at the NMR facility of the Department of Chemistry and Biochemistry of the University of Oklahoma using 99.8% CDCl_3_ (Cambridge Isotope Laboratories, Tewksbury, MA, USA). ^1^H and ^13^C chemical shifts were referenced to internal solvent resonances. Peak multiplicities were designated by s (singlet), d (doublet), t (triplet), q (quartet), m (multiplet), and br (broad), and chemical shifts were reported in parts per million (ppm). All NMR spectra were recorded at ambient temperature and processed using MestReNova software (Version 12.0.3, Mestrelab Research, S.L, Escondido, CA, USA, 2018).

### 3.6. HPLC Method A

Analytical scale reactions were analyzed via RP-HPLC employing a Gemini-NX C-18 (5 μm, 4.6 mm × 250 mm) column (Phenomenex, Torrance, CA, USA) (gradient of 25% B to 100% B over 25 min, 100% B for 3 min, 100% B to 25% B over 0.1 min, 25% B for 7 min (A = ddH_2_O with 0.1% TFA; B = acetonitrile); flow rate = 1 mL min^−1^; A_280_).

### 3.7. HPLC Method B

Semi-preparative RP-HPLC was conducted using a Gemini-NX C18 (5 μm, 10 mm × 250 mm) column (Phenomenex, Torrance, CA, USA) to purify scaled-up TPS analogs as well as TPS-A (gradient of 25% B to 40% B over 3 min, 40% B to 100% B over 22 min, 100% B for 3 min, 100% B to 25% B in 0.1 min, 25% B for 7 min (A = ddH_2_O with 0.1% formic acid; B = acetonitrile); flow rate = 2 mL min^−1^; A_280_).

### 3.8. Synthesis Tryprostatin A and B

**Figure 6.**
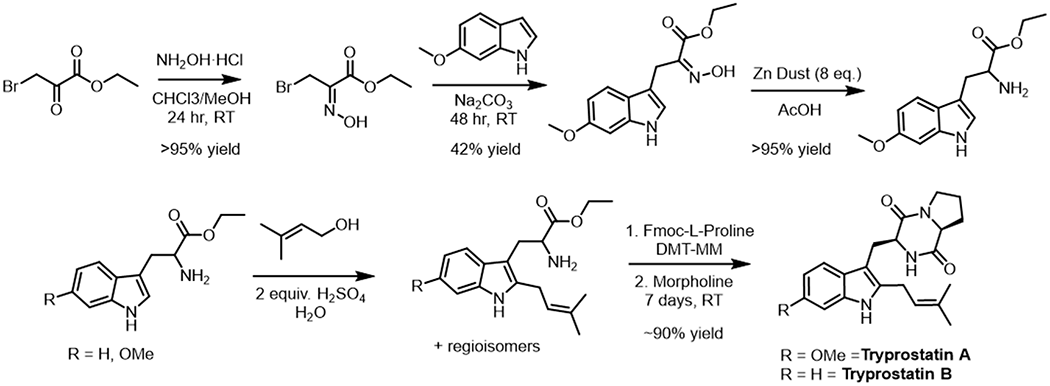
Synthetic schemes for the synthesis of 6-methoxytryptophan, TPS-A, and TPS-B using a previously reported biomimetic chemical prenylation.

Racemic 6-methoxy-tryptophan was prepared by the known reaction of ethyl bromo pyruvate oxime with 6-methoxy-indole followed by reduction via zinc dust in acetic acid [41]. TPS-A and TPS-B were both synthesized using a previously reported biomimetic prenylation reaction of their corresponding tryptophan ethyl esters, followed by DMT-MM mediated proline coupling and cyclization (Figure 6) [42].

### 3.9. Cell Titer-Blue Viability Assay

The K-562 cell line was obtained from ATCC (CCL-243^TM^). A cell titer-blue viability assay was performed in triplicate to evaluate TPS analog cytotoxicity. K-562 cells were cultured in RPMI Media (Thermo Catalog #: 22400105, Thermo Fisher Scientific, Waltham, MA, USA) containing 10% Hyclone (Fischer Sci Catalog #: SH3006603, Thermo Fisher Scientific, Waltham, MA, USA) and 1% penicillin–streptomycin (Thermo Catalog #: 15140122, Thermo Fisher Scientific, Waltham, MA, USA). Cells were incubated at 37 °C in 5.0% CO_2_. Cells were seeded out to 2000 cells per well in 75 μL of media within a 96-well plate. Taxol and Tryprostatin compounds were serial diluted in media to a 4× concentration, and 25 μL of each compound-containing solution were subsequently added to the 75 μL of cells to make a 1× concentration. Cells were incubated with compounds for 48 h at 37 °C in 5.0% CO_2_, followed by a 1.5 h incubation with Cell Titer-Blue. Fluorescence was measured using the Promega GloMax microplate reader at an excitation wavelength of 544 nm and an emission wavelength of 590 nm. Cell growth was normalized to untreated cells, and GI_50_ values were calculated using the four-parameter dose–response curve within GraphPad Prism software (GraphPad Software, San Diego, CA, USA, 2018).

## 4. Conclusions

This work has revealed the substrate promiscuity of CdpNPT toward alkyl-donors, combined with the high regiospecificity of the resulting reactions, these make CdpNPT a powerful biocatalyst for functionalizing the C6 position of TPS-B. Few synthetic methods are known for alkylating indole C6, and the existing methods are not suitable for the late-stage modification of natural products based on the complexity of the molecules. However, using prenyltransferase-based late-stage modification, we have developed a route to reach these previously inaccessible TPS-B analogs. Upon refining the substrate scope and regiospecificity of promiscuous PTs such as CdpNPT, NphB, and FgaPT2, these powerful biocatalysts are destined to become popular catalysts for synthetic late-stage modification and pharmaceutical production.

## Supplementary Material

Supplementary Material

## Figures and Tables

**Table 1.** Anticancer activity of TPS analogs against human leukemia K562 cells. The averaged GI_50_ values with associated standard deviation of compounds from three experimental replicates are shown.

Compound	K562 GI_50_ (μM)
TPS-A	97 ± 21
TPS-B	78 ± 31
TPS-2	54 ± 15
TPS-24	50 ± 29
TPS-25	56 ± 19
TPS-46	37 ± 8.8
TPS-52	77 ± 22
TPS-58	44 ± 18
TPS-61	89 ± 29
TPS-62	100 ± 33
TPS-65	53 ± 19
Taxol	0.0058 ± 0.0039
