# Supplementary material for: Indole C6 Functionalization of Tryprostatin B Using Prenyltransferase CdpNPT
Source: Catalysts. Author manuscript; Available in PMC 2021 Feb 25. (PMC7906295; doi:10.3390/catal10111247)
Supplement: Supplementary Material [file NIHMS1669452-supplement-Supplementary_Material.pdf]

catalysts

MDPI

Supplementary Materials

Indole C6 Functionalization of Tryprostatin B Using Prenyltransferase CdpNPT

Eric D. Gardner [1], Dustin A. Dimas [1], Matthew C. Finneran [1,†], Sara M. Brown [1], Anthony W. Burgett [2] and Shanteri Singh [1,*]

[1] Department of Chemistry and Biochemistry, University of Oklahoma; Norman, OK 73019, USA; ericgardner@ou.edu (E.D.G.); Dustin.Dimas-1@ou.edu (D.A.D.); mcfinner@umich.edu (M.C.F.); Sara.M.Brown-1@ou.edu (S.M.B.)
[2] Department of Pharmaceutical Sciences, University of Oklahoma; Oklahoma City, OK 73117, USA; Anthony-Burgett@ouhsc.edu
* Correspondence: Shanteri.singh@ou.edu
† Neuroscience Graduate Program, University of Michigan Medical School, Ann Arbor, MI 48109, USA

Table of contents

1. Figure S1: Analytical Scale CdpNPT Screening HPLC Traces…………..……….……… S2
2. Table S1: Raw HPLC data for analytical scale TPS reactions………………...…..………. S3
3. Figure S2: Cytotoxicity GI50 dose-response curves………………………..……..…….. S4
4. Table S2: HRMS data of positive reactions in TPS enzyme screening.…………..………. S5
5. Figure S3: Representative NMR correlations for structural assignment.………..………. S6
6. Table S3: NMR assignments of all purified TPS analogs……………………...…..… S6-S9
7. NMR spectra of TPS analogs……………………………………………………….... S10-S50
    a. NMR spectra for **TPS-A**…………………………..…………………..……….. S10
    b. NMR spectra for **TPS-B**……………………………….………………………. S11
    c. NMR spectra for **TPS-2**……………………………..……………...…….. S12-S16
    d. NMR spectra for **TPS-7**……………………………..…………………….. S17-S20
    e. NMR spectra for **TPS-16**…………………………………………………….. S21-S23
    f. NMR spectra for **TPS-24**…………………………………………………….. S24-S27
    g. NMR spectra for **TPS-25**…………………………………………………….. S28-S30
    h. NMR spectra for **TPS-46**…………….……………………………………..... S31-S34
    i. NMR spectra for **TPS-52**…………………………………………………….. S35-S37
    j. NMR spectra for **TPS-58**…………………………………………………….. S38-S41
    k. NMR spectra for **TPS-61**…………………………………………………….. S42-S43
    l. NMR spectra for **TPS-62**…………………………………………………….. S44-S46
    m. NMR spectra for **TPS-65**…………………………………………………….. S47-S50
8. Raw HRMS data of TPS analogs…………………………………….……………..... S51-S77

Analytical Scale CdpNPT Screening HPLC Traces

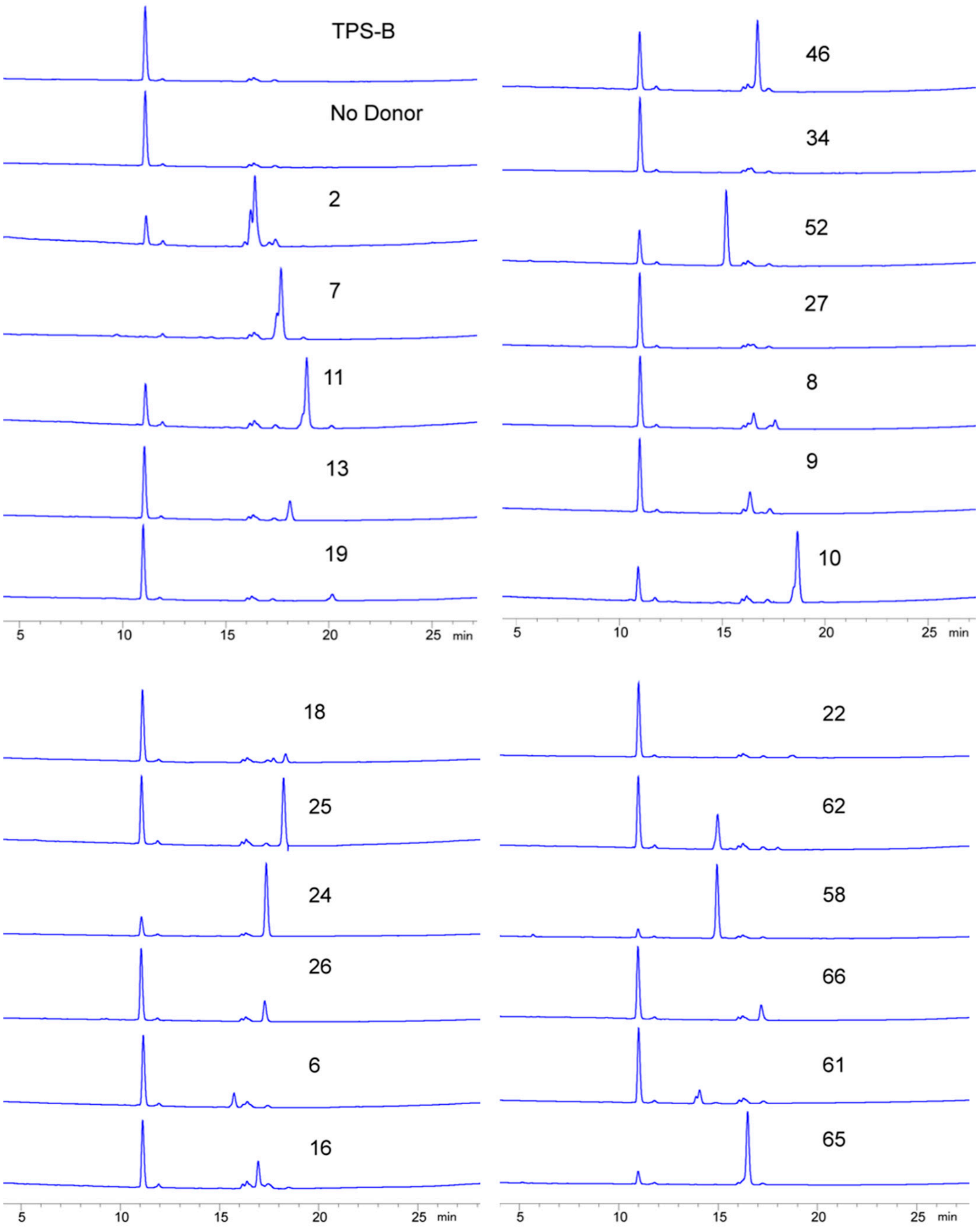

Figure S1. HPLC traces of analytical scale reactions containing TPS-B and CdpNPT under standard reaction conditions. Numerical labels indicate the alkyl-PP used in that reaction. TPS-B standard and enzymatic control reaction without the donor are indicated. The TPS-B used in these screenings is ~95% pure based on HPLC.

Table S1. Calculated percent turnover determined by HPLC in duplicate. Percent turnover was calculated by dividing the sum of all TPS-B and product peak areas by the peak area of remaining TPS-B.

OPP #	Trial 1 % Turnover	Trial 2 % Turnover	Mean % Turnover	Std Dev.
**2**	80.93	85.40	83.17	2.23
**6**	18.32	21.34	19.83	1.51
**7**	99.15	99.95	99.55	0.40
**8**	30.72	32.12	31.42	0.70
**9**	28.13	31.64	29.88	1.76
**10**	74.84	76.86	75.85	1.01
**11**	71.88	78.25	75.06	3.19
**13**	25.83	30.50	28.16	2.33
**16**	35.52	35.28	35.40	0.12
**18**	12.45	13.51	12.98	0.53
**19**	12.63	14.44	13.54	0.90
**22**	5.60	6.34	5.97	0.37
**24**	80.40	52.77	66.59	13.81
**25**	52.97	58.86	55.91	2.95
**26**	24.63	26.63	25.63	1.00
**27**	7.36	8.00	7.68	0.32
**34**	7.45	8.79	8.12	0.67
**46**	57.66	64.90	61.28	3.62
**52**	69.94	75.40	72.67	2.73
**58**	89.96	92.27	91.11	1.15
**61**	16.45	18.73	17.59	1.14
**62**	36.29	38.85	37.57	1.28
**65**	87.13	90.40	88.76	1.64
**66**	20.38	24.08	22.23	1.85

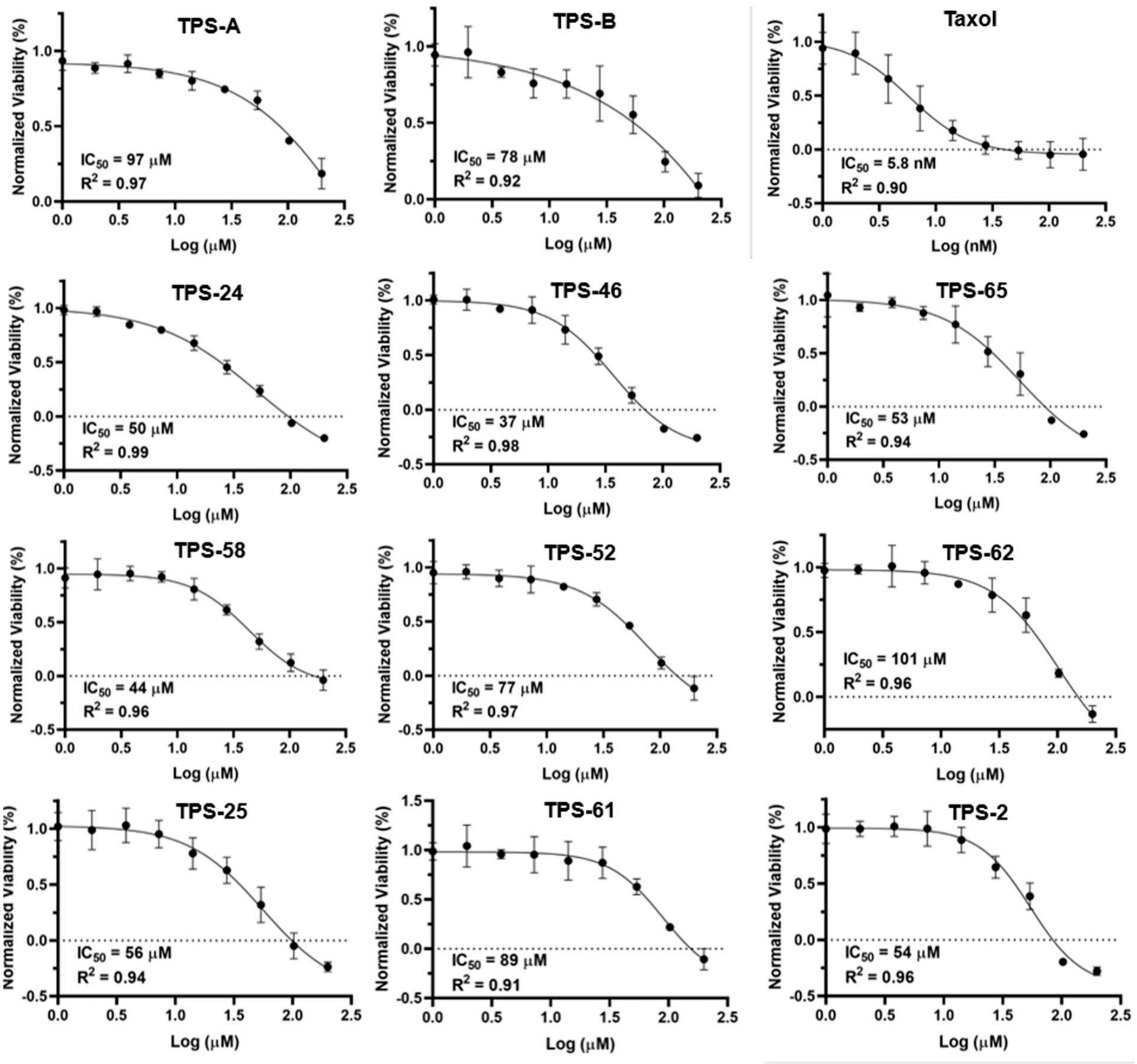

Figure S2. Cytotoxicity assay results for TPS analogs run in triplicate against K563 leukemia cells, with TPS-A, TPS-B, and Taxol as positive controls.

Table S2. HRMS data of the TPS analogs produced by CdpNPT and their corresponding alkyl donor

TPSB analog	Formula	Calculated Mass	Observed Mass
**TPS-A**	$C_{22}H_{27}N_3O_3$ [M+H]$^+$	382.2125	382.2133
**TPS-B**	$C_{21}H_{25}N_3O_2$ [M+H]$^+$	352.20194	352.203
**TPS-2**	$C_{26}H_{33}N_3O_2$ [M+H]$^+$	420.26453	420.2659
**TPS-6**	$C_{26}H_{31}N_3O_2$ [M+H]$^+$	418.241627	418.2498
**TPS-7**	$C_{27}H_{35}N_3O_2$ [M+H]$^+$	434.272927	434.2819
**TPS-8**	$C_{27}H_{33}N_3O_2$ [M+H]$^+$	432.257277	432.265
**TPS-9**	$C_{27}H_{33}N_3O_2$ [M+H]$^+$	432.257277	432.2652
**TPS-10**	$C_{28}H_{37}N_3O_2$ [M+H]$^+$	448.288577	448.2971
**TPS-11**	$C_{28}H_{37}N_3O_2$ [M+H]$^+$	448.288577	448.2964
**TPS-13**	$C_{28}H_{35}N_3O_2$ [M+H]$^+$	446.272927	446.2809
**TPS-16**	$C_{27}H_{33}N_3O_2$ [M+H]$^+$	432.257277	432.2653
**TPS-18**	$C_{28}H_{35}N_3O_2$ [M+H]$^+$	446.272927	446.2810
**TPS-19**	$C_{29}H_{39}N_3O_2$ [M+H]$^+$	462.304226	462.3118
**TPS-22**	$C_{29}H_{37}N_3O_2$ [M+H]$^+$	460.288577	460.2964
**TPS-24**	$C_{30}H_{33}N_3O_2$ [M+H]$^+$	468.26453	468.2651
**TPS-25**	$C_{31}H_{35}N_3O_2$ [M+H]$^+$	482.28018	482.2802
**TPS-26**	$C_{31}H_{35}N_3O_3$ [M+H]$^+$	498.267842	498.2758
**TPS-27**	$C_{29}H_{35}N_3O_2$ [M+H]$^+$	458.272927	458.2814
**TPS-34**	$C_{27}H_{34}N_6O_2$ [M+H]$^+$	475.274324	475.2823
**TPS-46**	$C_{29}H_{33}N_3O_2$ [M+H]$^+$	456.26453	456.2655
**TPS-52**	$C_{29}H_{33}N_3O_3$ [M+H]$^+$	472.25945	472.2602
**TPS-58**	$C_{29}H_{31}N_3O_4$ [M+H]$^+$	486.23871	486.2389
**TPS-61-P1**	$C_{26}H_{29}N_3O_3$ [M+H]$^+$	432.22815	432.2292
**TPS-61-P2**	$C_{26}H_{29}N_3O_3$ [M+H]$^+$	432.22815	432.2294
**TPS-62**	$C_{26}H_{29}N_3O_2S$ [M+H]$^+$	448.20531	448.2061
**TPS-65**	$C_{30}H_{31}N_3O_3$ [M+H]$^+$	482.2438	482.2442
**TPS-66**	$C_{30}H_{31}N_3O_2S$ [M+H]$^+$	498.213699	498.2216

Table S3. Tryprostatin Analog NMR Assignment Table

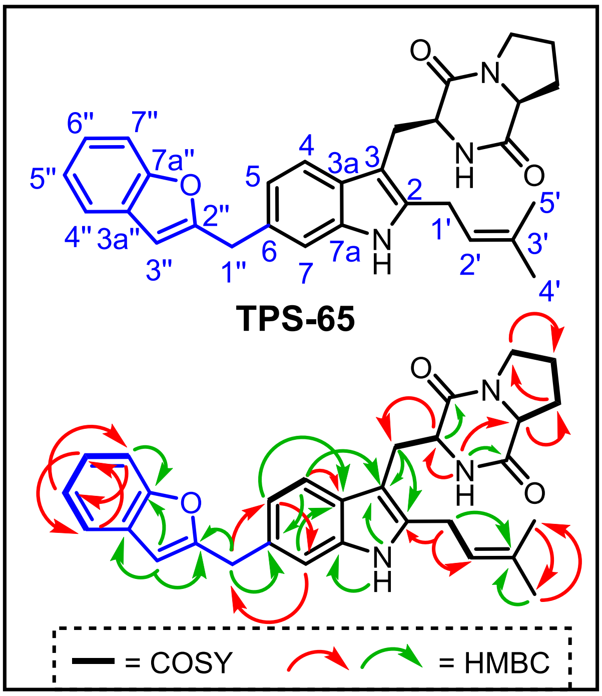

Figure S3: Representative NMR correlations used to assign TPS analogs

Segment	Position	δC (in ppm), type	δH (in ppm)
L-Trp	NH		5.62 (s)
	α	54.5, CH	4.33 (dd)
	β	25.6, CH_2	2.93 (m), 3.66 (d)
	1		7.98 (s)
	2	136.5, C	
	3	104.5, C	
	3a	126.8, C	
	4	117.9, CH	7.4 (d)
	5	121.3, CH	7.04 (d)
	6	131.0, C	
	7	111.1, CH	7.21 (s)
	7a	136.1, C	
	C=O		
Proline	α	59.3, CH	4.04 (t)
	β	28.4, CH_2	2.02 (m), 2.32 (dt)
	γ	22.7, CH_2	1.89 (m), 2.02 (m)
	δ	45.4, CH_2	3.58 (m), 3.65 (m)
	C=O	165.8, C	
C2-Prenyl	1'	25.1, CH_2	3.44 (qd)
	2'	119.8, CH	5.28 (m)
	3'	135.5, C	
	4'	18.0, CH_3	1.73 (s)
	5'	25.8, CH_3	1.76 (s)
C6-Sub	1''	35.2, CH_2	4.18 (s)
	2''	158.6, C	
	3''	103.3, CH	6.36 (s)
	3a''	128.9, C	
	4''	111.0, CH	7.38 (d)
	5''	123.4, CH	7.18 (td)
	6''	122.6, CH	7.15 (td)
	7''	120.5, CH	7.43 (d)
	7a''	155.0, C	

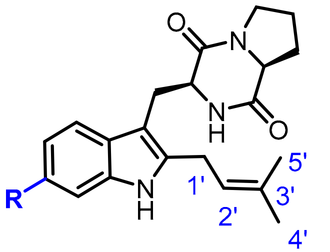

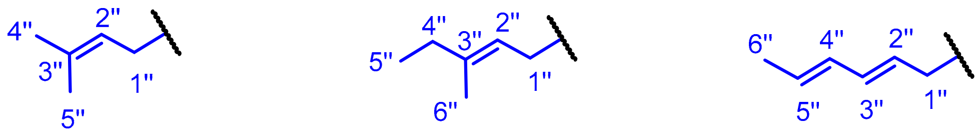

		TPS-2		TPS-7		TPS-16	
**Segment**	**Position**	**δC , type**	**δH**	**δC , type**	**δH**	**δC , type**	**δH**
L-Trp	NH		5.61 (s)		5.61 (s)		5.61 (s)
	α	54.6, CH	4.36 (m)	54.6, CH	4.34 (dd)	54.6, CH	4.35 (dd)
	β	25.5 CH_2	2.93 (dd), 3.66 (m)	25.6, CH_2	2.92 (dd), 3.64 (m)	25.7, CH_2	2.93 (dd), 3.65 (m)
	1		7.82 (s)		7.81 (s)		
	2						
	3						
	3a						
	4	117.7, CH	7.37 (d)	117.6, CH	7.37 (d)	117.6, CH	7.38 (d)
	5	121, CH	6.94 (d)	121.0, CH	6.94 (d)	121.1, CH	6.94 (d)
	6						
	7	110.1, CH	7.12 (s)	110.1, CH	7.11 (s)	110.5, CH	7.13
	7a						
	C=O						
Proline	α	59.3, CH	4.05 (t)	59.6, CH	4.05 (t)	59.3, CH	4.06 (t)
	β	28.4, CH_2	2.05 (m), 2.35 (m)	28.3, CH_2	2.04 (m), 2.33 (m)	28.4, CH_2	2.05 (m), 2.35 (m)
	γ	22.7, CH_2	1.92 (m), 2.05 (m)	22.6, CH_2	1.91 (m), 2.04 (m)	22.9, CH_2	1.92 (m), 2.05 (m)
	δ	45.5, CH_2	3.59 (m), 3.70 (m)	45.4, CH_2	3.59 (m), 3.67 (m)	45.4, CH_2	3.59 (m), 3.68 (m)
	C=O						
C2-Prenyl	1'	25.2, CH_2	3.46 (d)	25.1, CH_2	3.45 (m)	25.2, CH_2	3.46 (m)
	2'	119.8, CH	5.31 (t)	119.8, CH	5.29 (t)	119.6, CH	5.31 (t)
	3'						
	4'	17.8, CH_3	1.75 (s)	18.0, CH_3	1.74 (s)	18.0, CH_3	1.75 (s)
	5'	25.7, CH_3	1.76 (s)	25.7, CH_3	1.78 (s)	25.8, CH_3	1.79 (s)
C6-Sub	1''	36.5, CH_2	3.42 (d)	34.3, CH_2	3.44 (m)	39.1, CH_2	3.48 (m)
	2''	124.2, CH	5.37 (t)	122.3, CH	5.36	131.0, CH	5.72 (dt)
	3''					131.4, CH	6.06 (m)
	4''	17.8, CH_3	1.75 (s)	32.4, CH_2	2.05 (q)	131.4, CH	6.06 (m)
	5''	25.7, CH_3	1.79 (s)	12.7, CH_3	1..02 (t)	127.7, CH	5.63 (m)
	6''			16.0, CH_3	1.73 (s)	18.0, CH_3	1.73 (d)

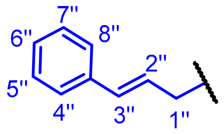

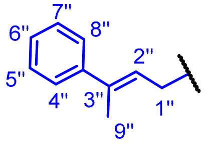

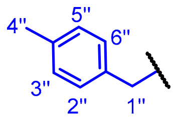

		TPS-24		TPS-25		TPS-46	
**Segment**	**Position**	**δC , type**	**δH**	**δC , type**	**δH**	**δC , type**	**δH**
L-Trp	NH		5.62 (s)		5.5 (s)		5.6 (s)
	α	54.5, CH	4.35 (dd)	54.7, CH	4.36 (dd)	54.5, CH	4.34 (dd)
	β	25.7, CH_2	2.92 (m), 3.66 (m)	25.7, CH_2	2.93 (dd), 3.64 (m)	25.7, CH_2	2.91 (m), 3.65 (m)
	1		7.95 (s)		7.83 (s)		7.87 (s)
	2	135.9, C				136.0, C	
	3	104.4, C				104.4, C	
	3a	126.5, C				126.3, C	
	4	117.5, CH	7.40 (d)	117.8, CH	7.39 (d)	117.6, CH	7.36 (d)
	5	120.9, CH	6.99 (d)	121.1, CH	6.99 (dd)	121.3, CH	6.95 (d)
	6	134.1, C				135.7, C	
	7	110.5, CH	7.17 (s)	110.3, CH	7.17 (s)	110.9, CH	7.06 (s)
	7a	135.9, C					
	C=O						
Proline	α	59.2, CH	4.04 (t)	59.3, CH	4.05 (t)	59.3, CH	4.04 (t)
	β	28.4, CH_2	2.02 (m), 2.32 (dt)	28.4, CH_2	2.03 (m), 2.33 (dt)	28.3, CH_2	2.02 (m), 2.32 (dt)
	γ	22.8, CH_2	1.89 (m), 2.02 (m)	22.7, CH_2	1.91 (m), 2.03 (m)	22.6, CH_2	1.90 (m), 2.02 (m)
	δ	45.4, CH_2	3.58 (m), 3.66 (m)	45.5, CH_2	3.58 (m), 3.67 (m)	45.4, CH_2	3.58 (m), 3.66 (m)
	C=O	165.9, C				165.9, C	
C2-Prenyl	1'	25.2, CH_2	3.44 (qd)	25.2, CH_2	3.45 (m)	25.0, CH_2	3.43 (qd)
	2'	119.6, CH	5.29 (t)	119.9, CH	5.29 (t)	119.8, CH	5.26 (t)
	3'	135.4, C				135.4, C	
	4'	18.0, CH_3	1.73 (s)	18.0, CH_3	1.74 (s)	17.9, C	1.72 (s)
	5'	25.8, CH_3	1.76 (s)	25.8, CH_3	1.78 (s)	25.7	1.76 (s)
C6-Sub	1''	39.5, CH_2	3.62 (d)	35.2, CH_2	3.65 (d)	41.7, CH_2	4.02 (s)
	2''	130.2, CH	6.44 (m)	127.4, CH	6.02 (t)	128.9, CH	7.08 (s)
	3''	129.2, CH	6.4 (m)			128.9, CH	7.08 (s)
	4''	137.7, C		125.8, CH	7.41 (m)	21.0, CH_3	2.30 (s)
	5''	125.9, CH	7.35 (d)	128.2, CH	7.29 (t)	128.9, CH	7.08 (s)
	6''	128.2, CH	7.27 (t)	126.7, CH	7.21 (t)	128.9, CH	7.08 (s)
	7''	126.8, CH	7.18 (t)	128.2, CH	7.29 (t)		
	8''	128.2, CH	7.27 (t)	125.8, CH	7.41 (m)		
	9''	125.9, CH	7.35 (d)	16.0, CH_3	2.16 (s)		

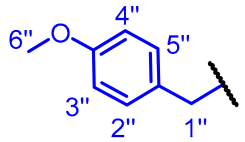

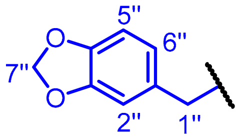

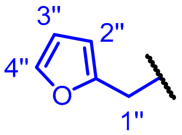

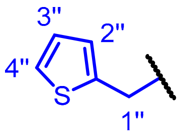

		TPS-52		TPS-58		TPS-61	TPS-62	
**Segment**	**Position**	**δC , type**	**δH**	**δC , type**	**δH**	**δH**	**δH**	**δC , type**
L-Trp	NH		5.62 (s)			5.60 (s)	5.61 (s)	
	α	54.5, CH	4.33 (dd)	54.5, CH	4.33 (dd)	4.35 (d)	4.36 (dd)	54.6, CH
	β	25.0, CH_2	2.91 (dd), 3.64 (m)	25.7, CH_2	2.91 (m), 3.63 (d)	2.93 (m), 3.66 (m)	2.93 (dd), 3.63 (m)	25.5, CH_2
	1		7.82 (s)		7.91 (s)	7.86 (s)	7.85 (s)	
	2			136.0, C				
	3			104.5, C				
	3a			126.3, C				
	4	117.7, CH	7.37 (d)	117.5, CH	7.37 (d)	7.40 (d)	7.40 (d)	117.7, CH
	5	121.2, CH	6.95 (d)	121.1, CH	6.93 (dd)	7.00 (d)	7.02 (dd)	121.1, CH
	6			135.6, C				
	7	110.6, CH	7.05 (s)	110.7, CH	7.07 (s)	7.16 (s)	7.16 (s)	110.7, CH
	7a			136.1, C				
	C=O							
Proline	α	59.7, CH	4.04 (t)	59.3, CH	4.04 (t)	4.05 (s)	4.05 (t)	59.3, CH
	β	28.5, CH_2	2.03 (m), 2.32 (m)	28.4, CH_2	2.02 (m), 2.32 (dt)	2.04 (m), 2.33 (m)	2.03 (m), 2.31 (m)	28.5, CH_2
	γ	22.6, CH_2	1.90 (m), 2.03 (m)	22.7, CH_2	1.89 (m), 2.02 (m)	1.90 (m), 2.33 (m)	1.89 (m), 2.01 (m)	22.8, CH_2
	δ	45.4, CH_2	3.58 (m), 3.66 (m)	45.4, CH_2	3.58 (m), 3.66 (m)	3.60 (m), 3.66 (m)	3.58 (t), 3.67 (m)	45.4, CH_2
	C=O			165.9, C				
C2-Prenyl	1'	25.2, CH_2	3.43 (m)	25.2, CH_2	3.43 (qd)	3.45 (m)	3.44 (t)	25.0, CH_2
	2'	119.5, CH	5.28 (t)	119.8, CH	5.27 (m)	5.29 (t)	5.30 (t)	119.7, CH
	3'			135.5, C				
	4'	18.1, CH_3	1.73 (s)	18.1, CH_3	1.73 (s)	1.74 (s)	1.74 (s)	17.7, CH_3
	5'	25.7, CH_3	1.76 (s)	25.9, CH_3	1.76 (s)	1.78 (s)	1.78 (s)	25.8, CH_3
C6-Sub	1''	41.1, CH_2	4.00 (s)	41.8, CH	3.96 (s)	4.05 (s)	4.23 (s)	36.3, CH_2
	2''	129.7, CH	7.11 (d)	109.2, CH	6.66 (m)	6.00 (d)	6.81 (dd)	125.1, CH
	3''	113.8, CH	6.81 (d)	145.7, C		6.28 (d)	6.92 (dd)	126.8, CH
	4''	113.8, CH	6.81 (d)	147.5, C		7.32 (s)	7.13 (dd)	123.9, CH
	5''	129.7, CH	7.11 (d)	108.0, CH	6.71 (d)			
	6''	55.2, CH_3	3.77 (s)	121.5, CH	6.66 (m)			
	7''			100.8, CH_2	5.88 (s)			

NMR Spectra for Tryprostatin Analogs:

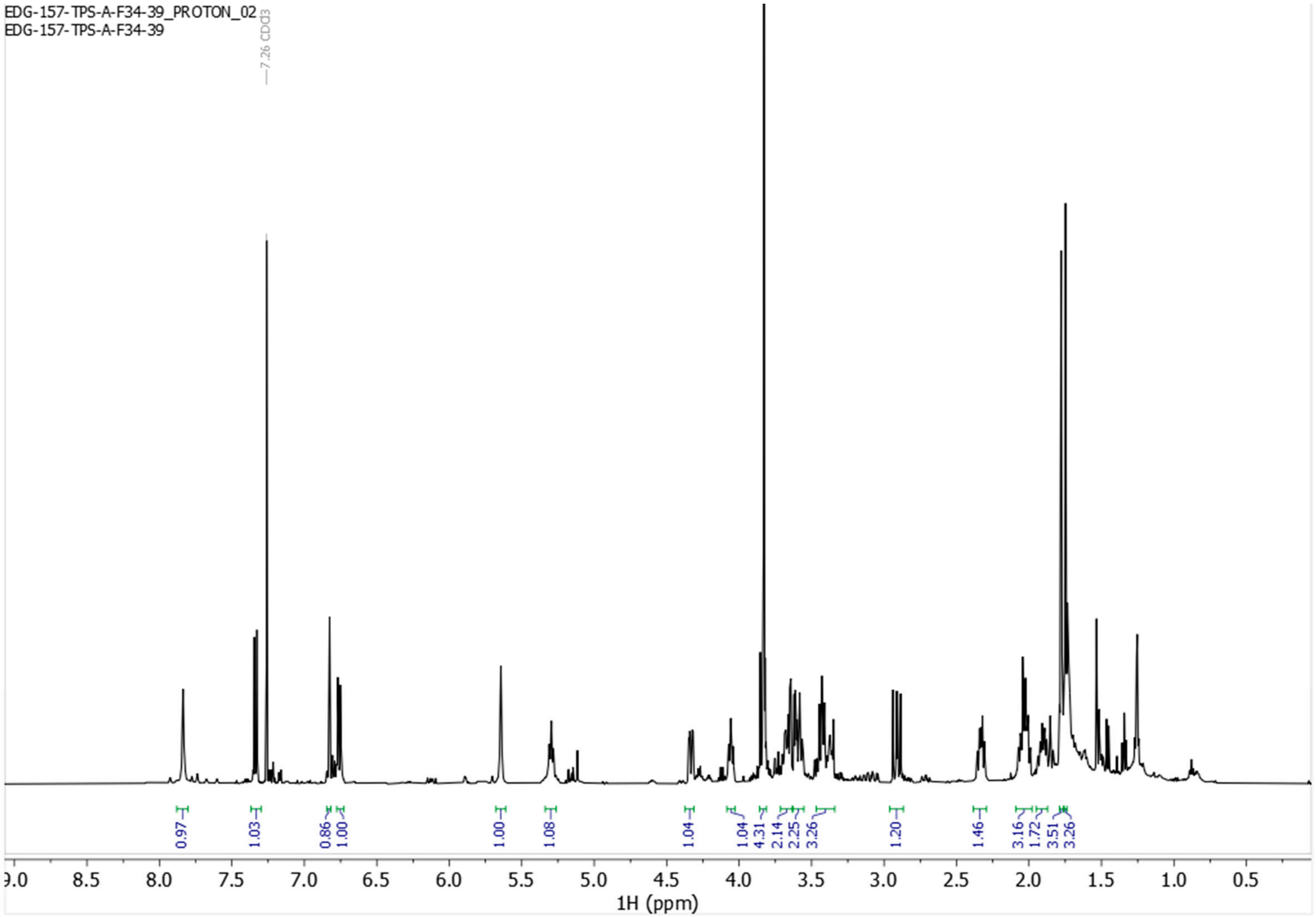

^{1}H NMR spectrum (500 MHz) of **TPS-A** in $CDCl_3$

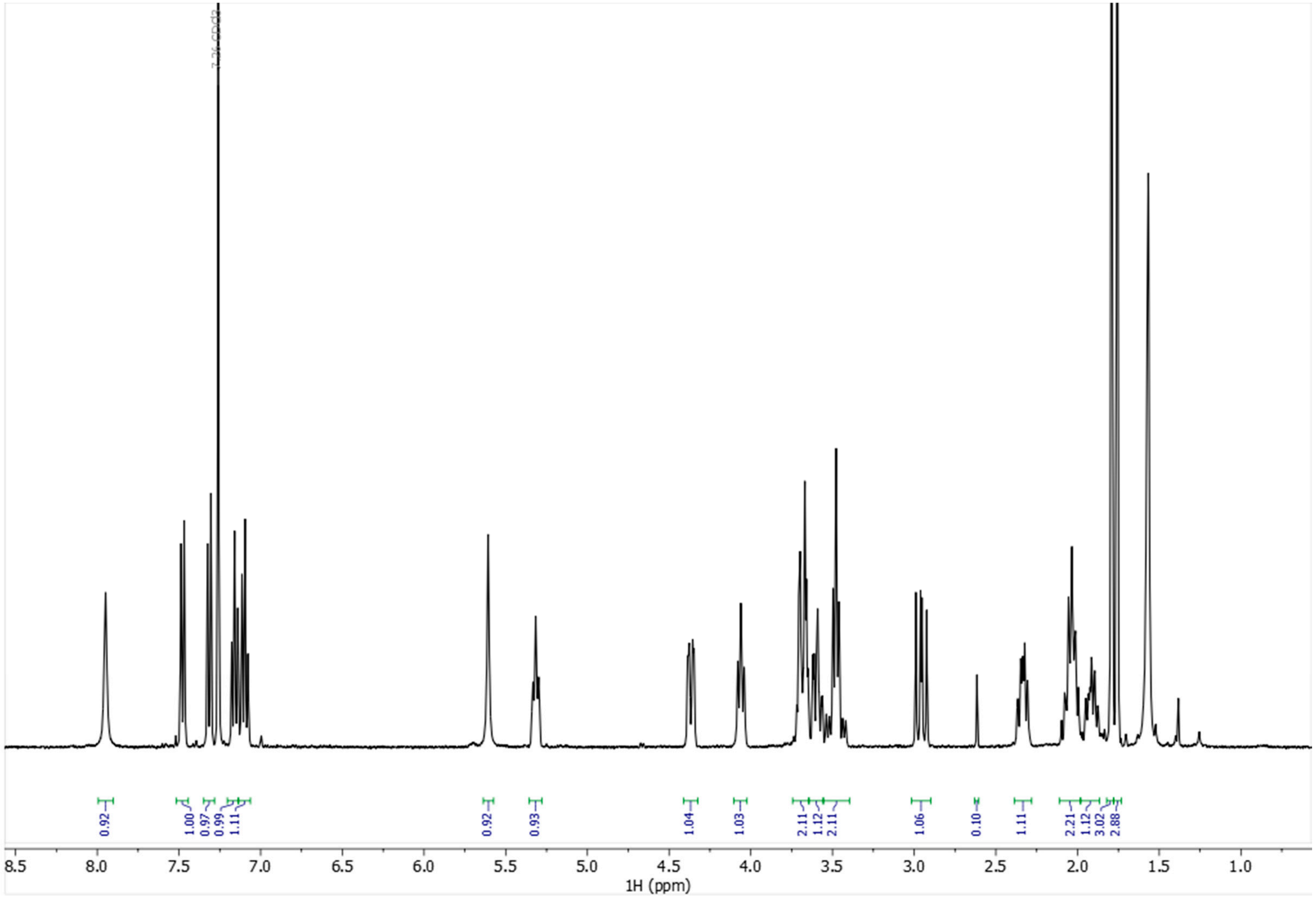

^{1}H NMR spectrum (400 MHz) of **TPS-B** in $CDCl_3$

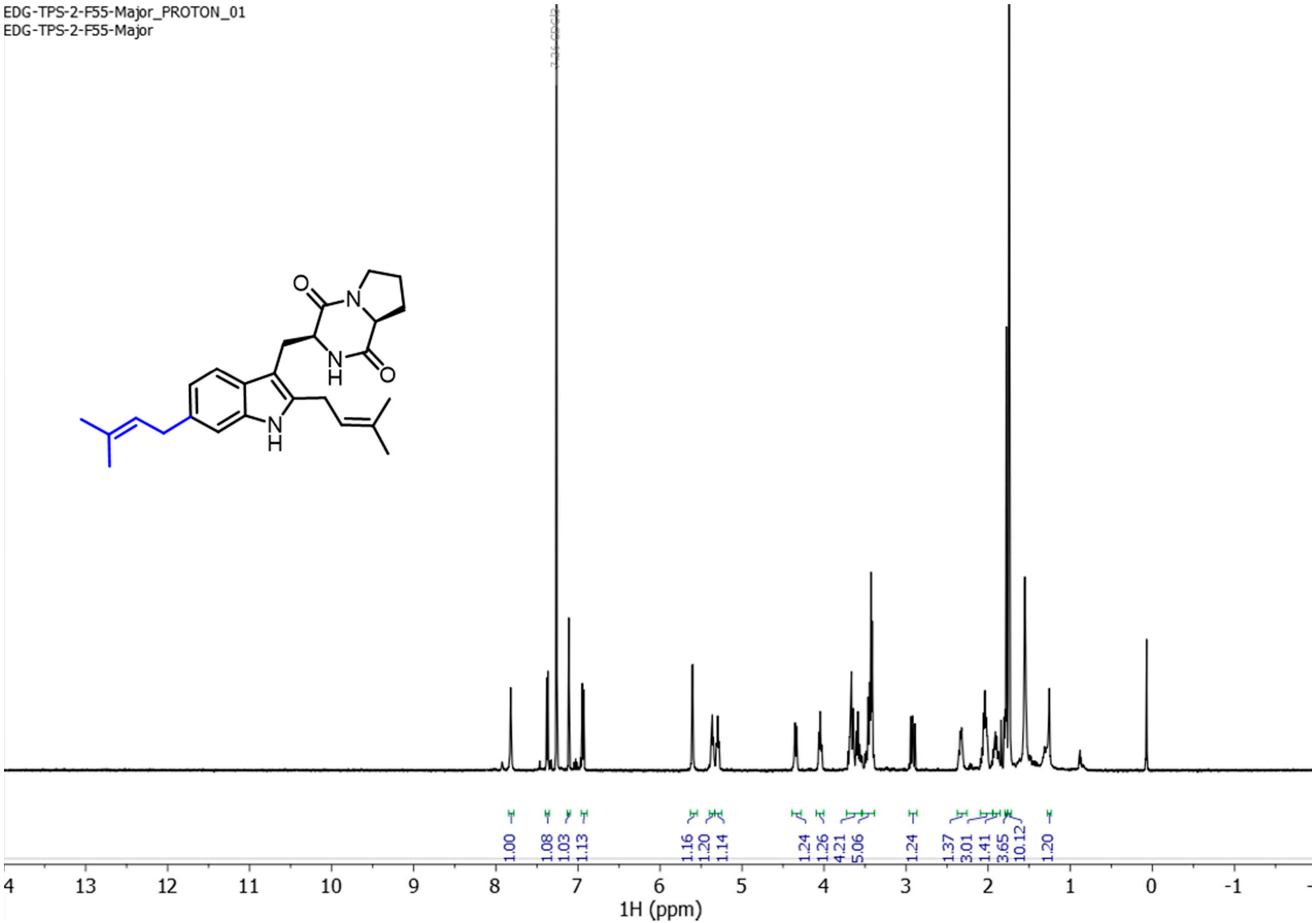

^{1}H NMR spectrum (500 MHz) of **TPS-2-C6** in $CDCl_3$

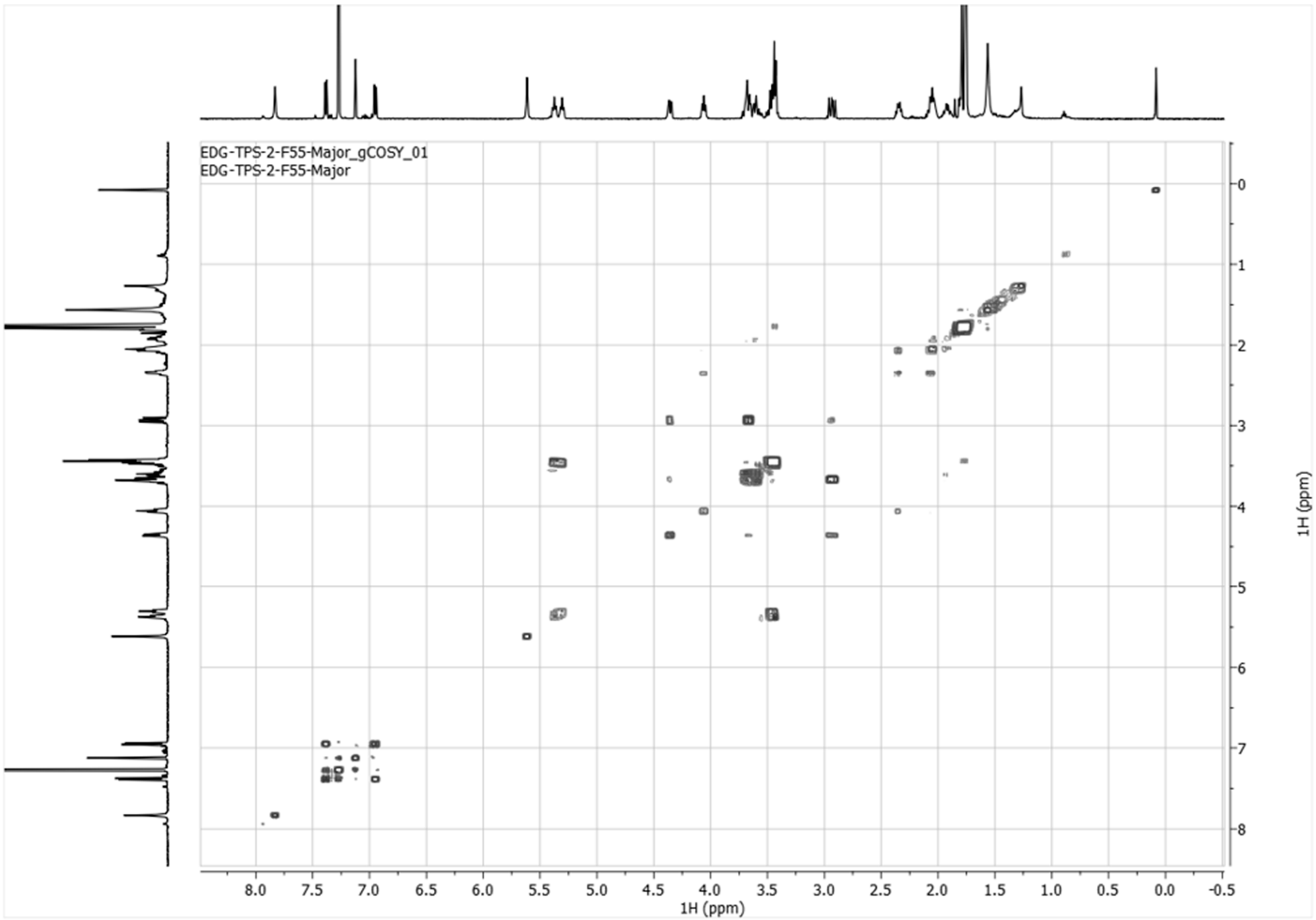

COSY NMR spectrum (500 MHz) of **TPS-2-C6** in $CDCl_3$

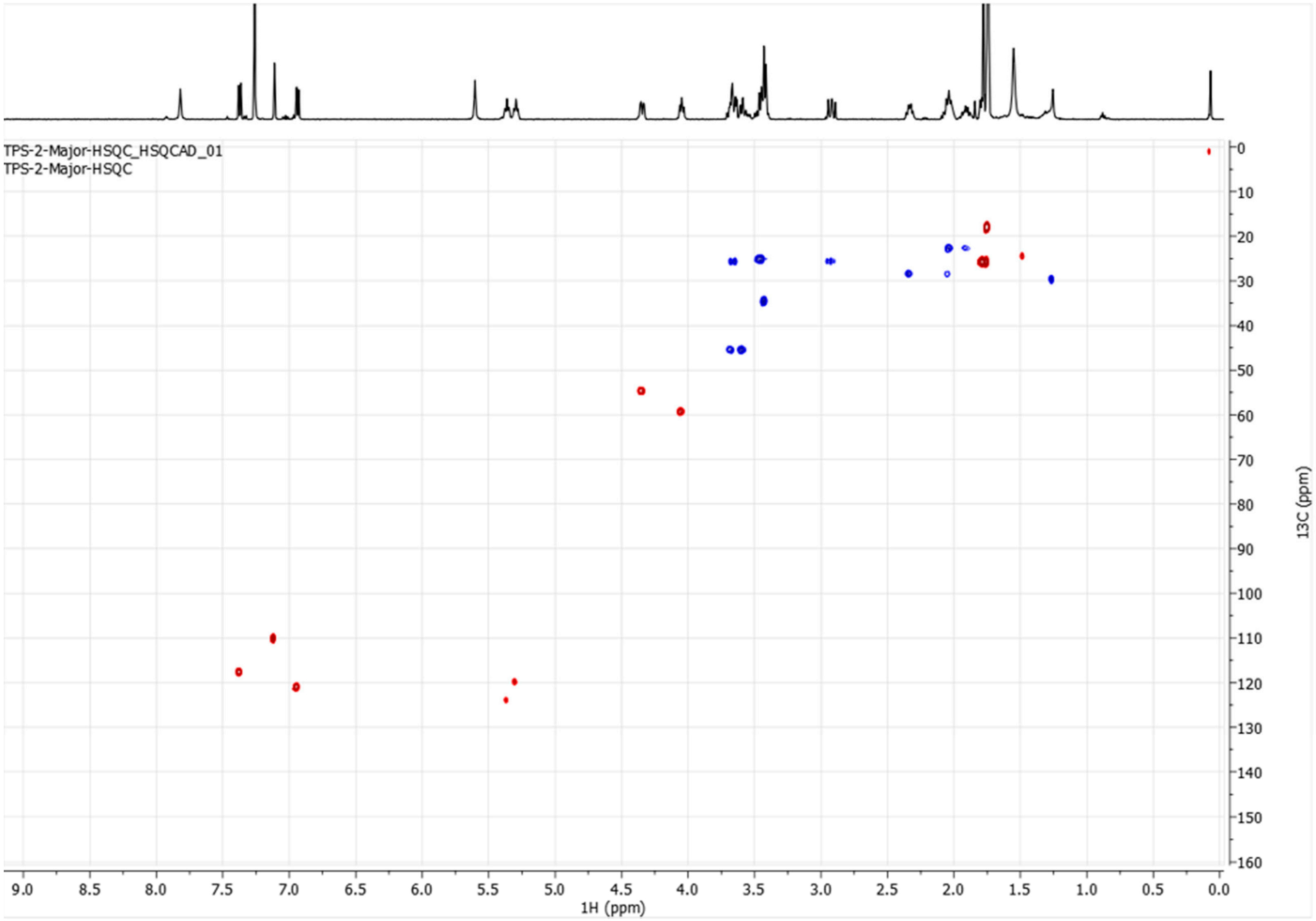

HSQC NMR spectrum (500 MHz) **of TPS-2-C6** in $CDCl_3$

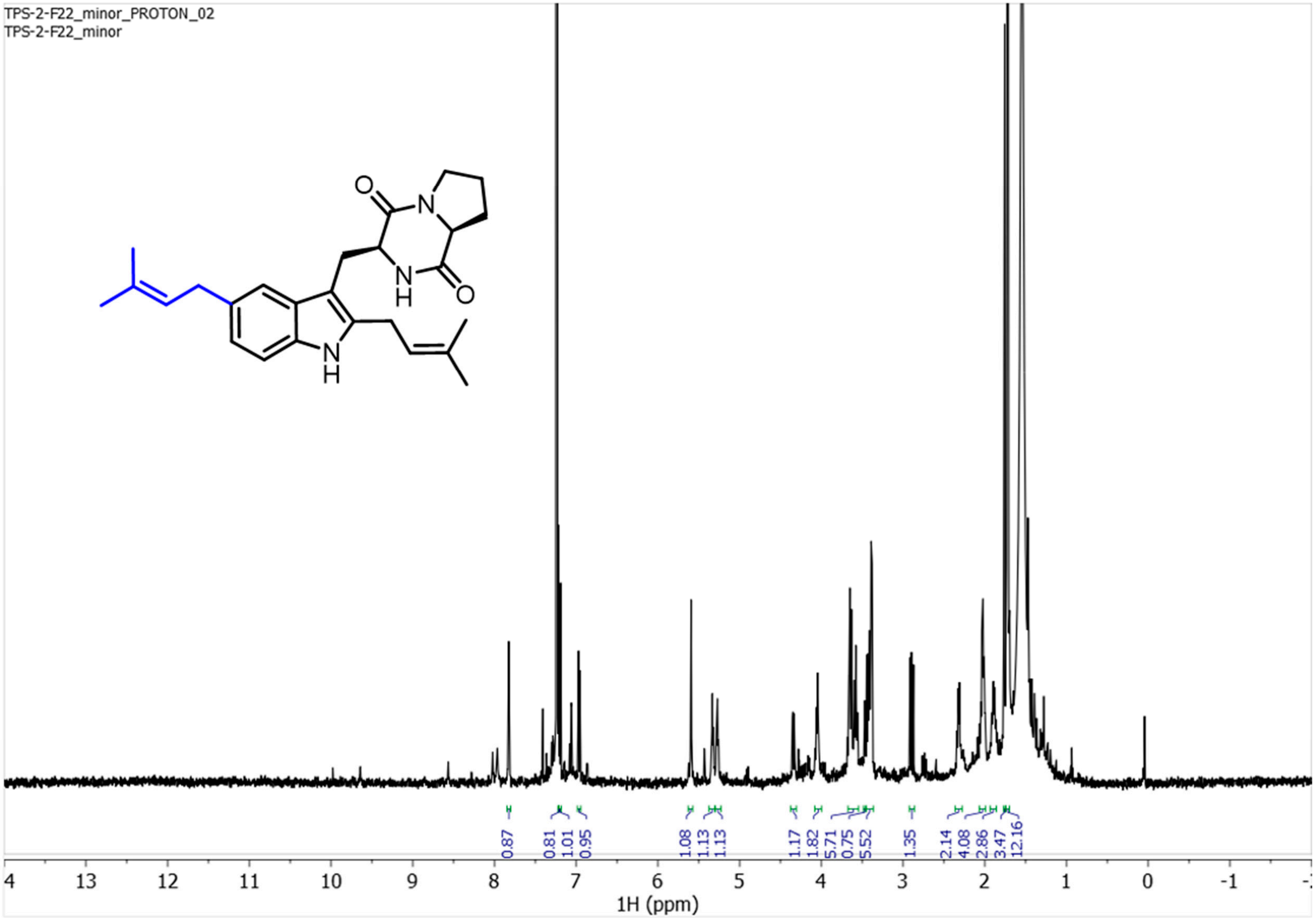

^{1}H NMR spectrum (500 MHz) **of TPS-2-C5** in $CDCl_3$

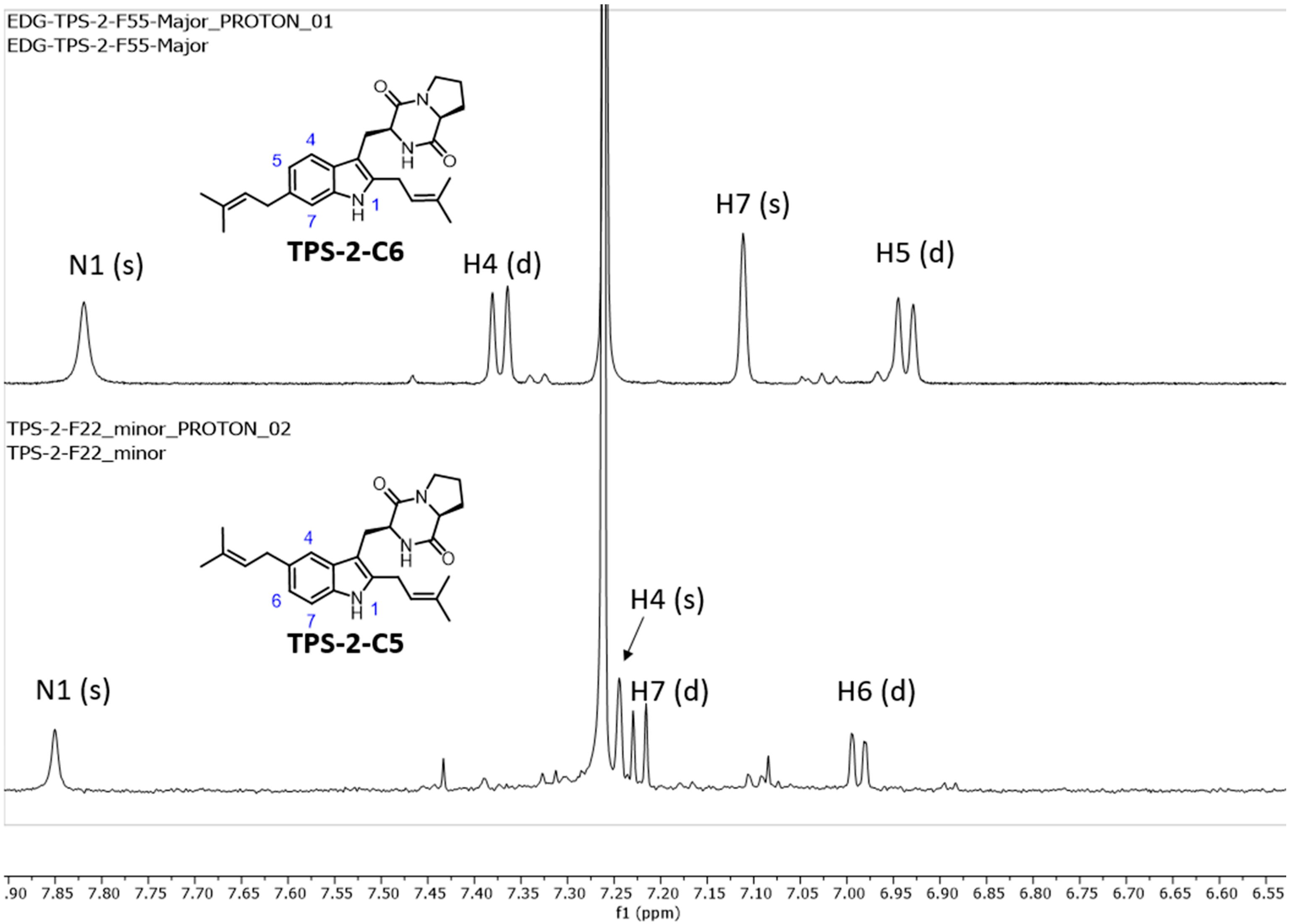

Stacked ^{1}H NMR Spectra (500 MHz) of isolated **TPS-2** regioisomers in $CDCl_3$

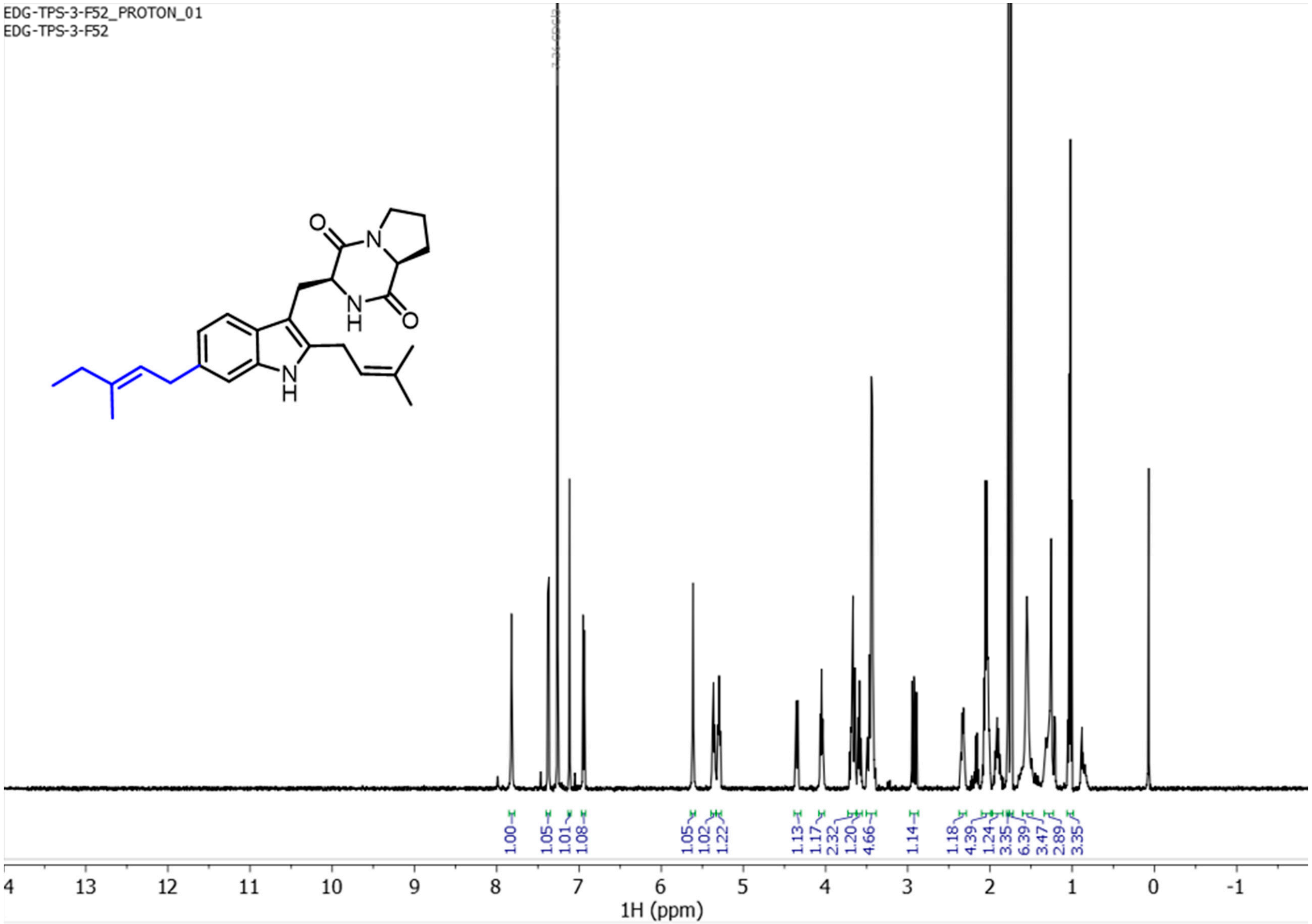

^{1}H NMR spectrum (500 MHz) of **TPS-7** in $CDCl_3$

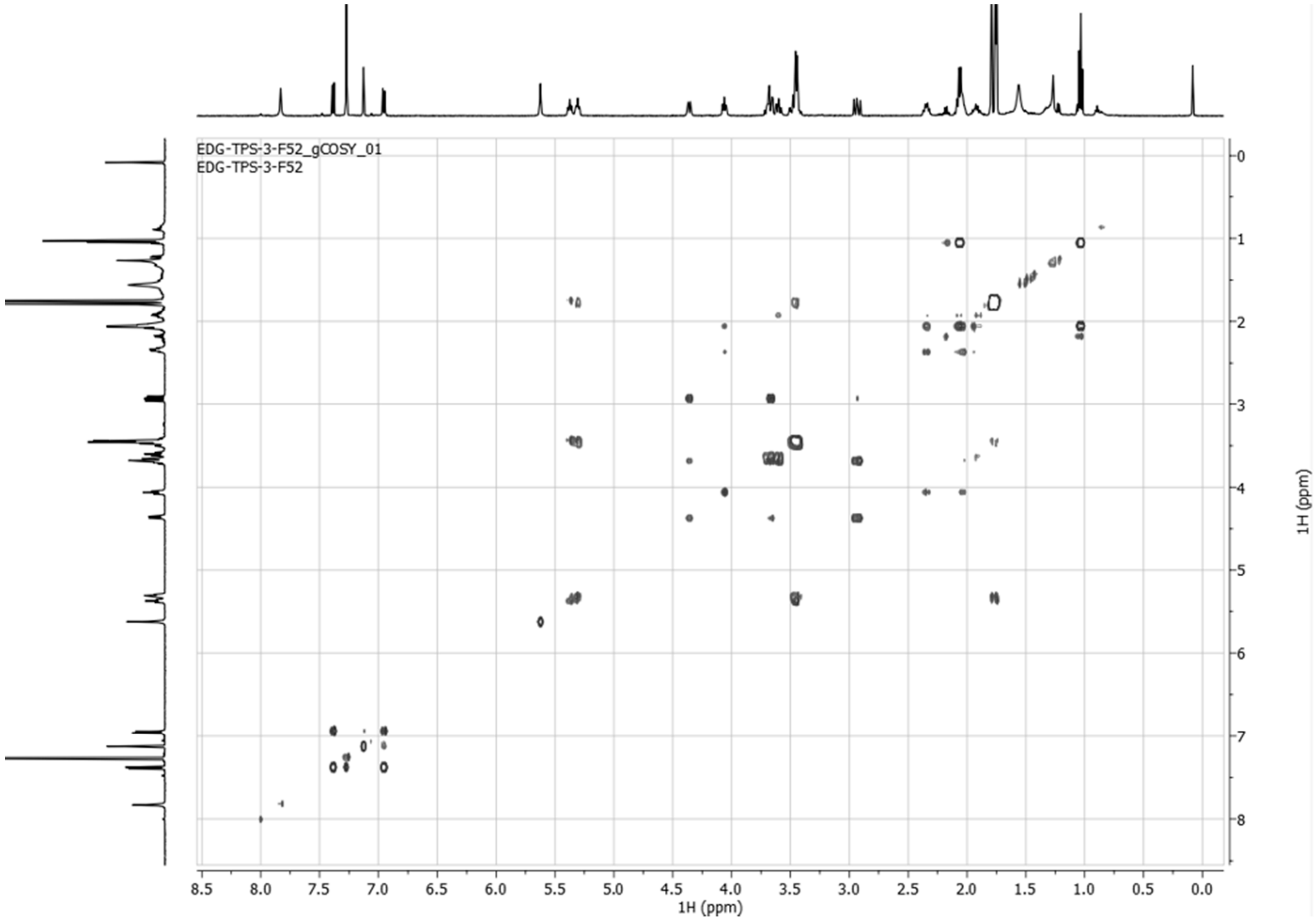

COSY spectrum (500 MHz) of **TPS-7** in $CDCl_3$

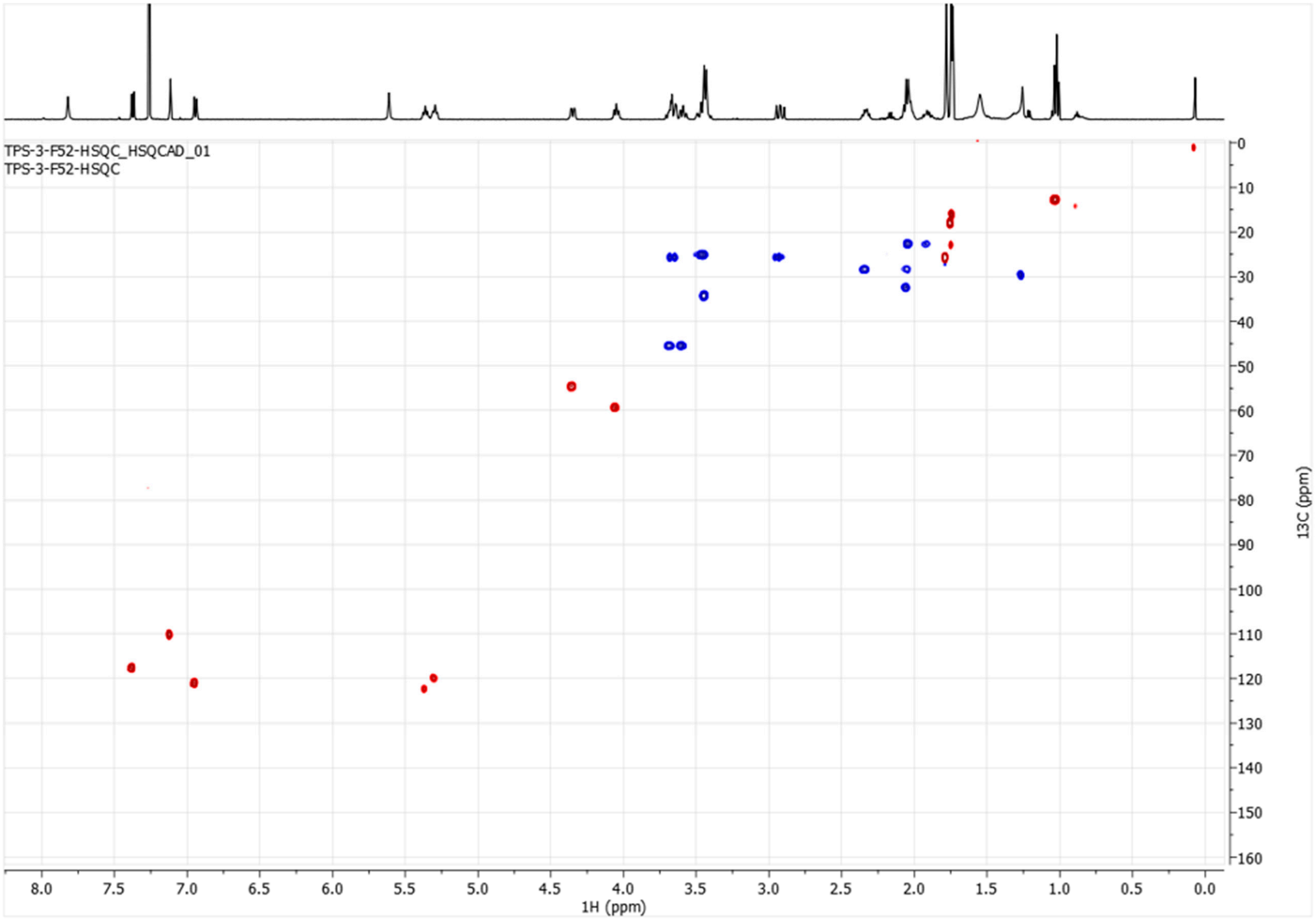

HSQC NMR spectrum (500 MHz) of **TPS-7** in $CDCl_3$

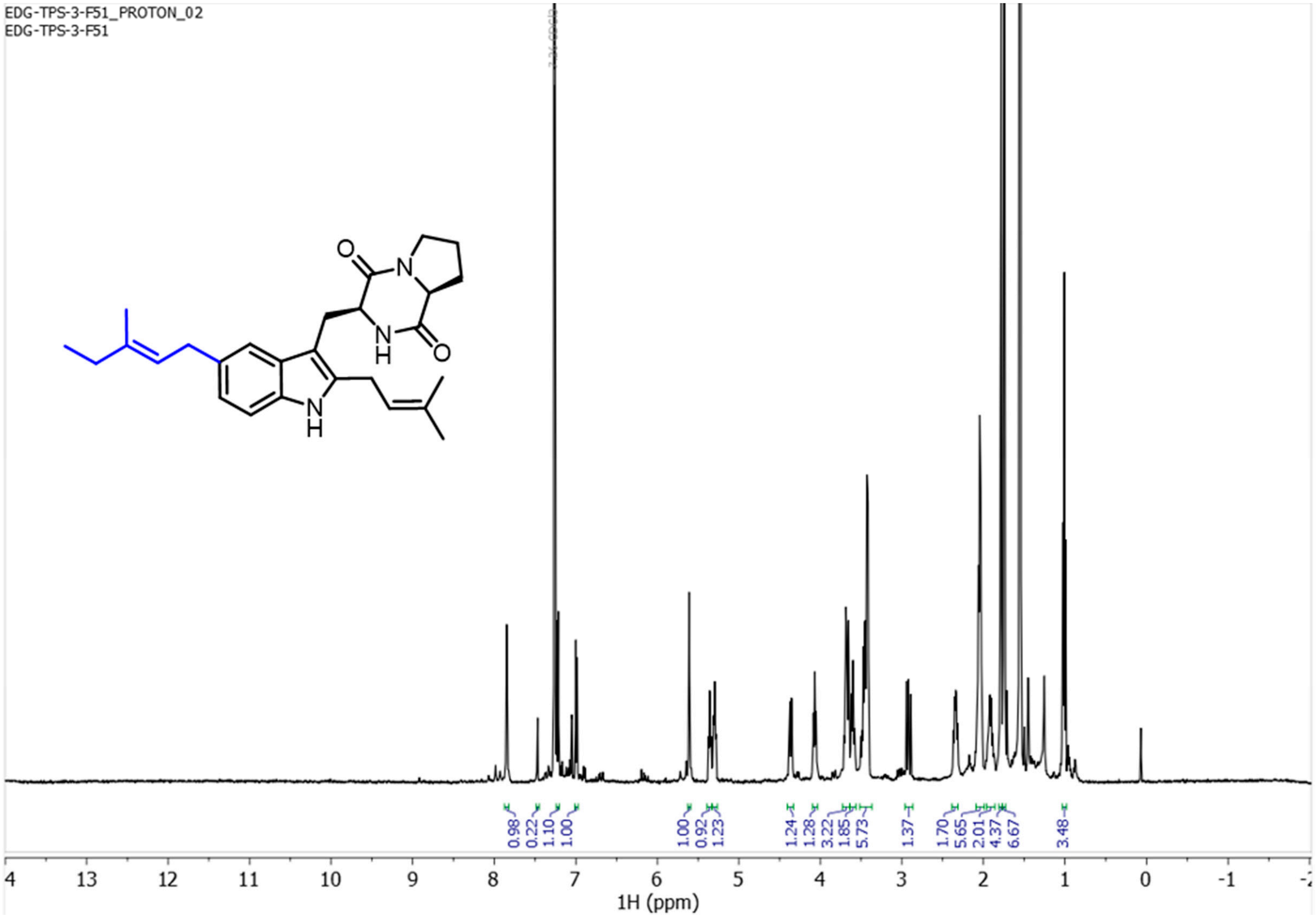

^{1}H NMR spectrum (500 MHz) of **TPS-7-C5** (minor product) in $CDCl_3$.

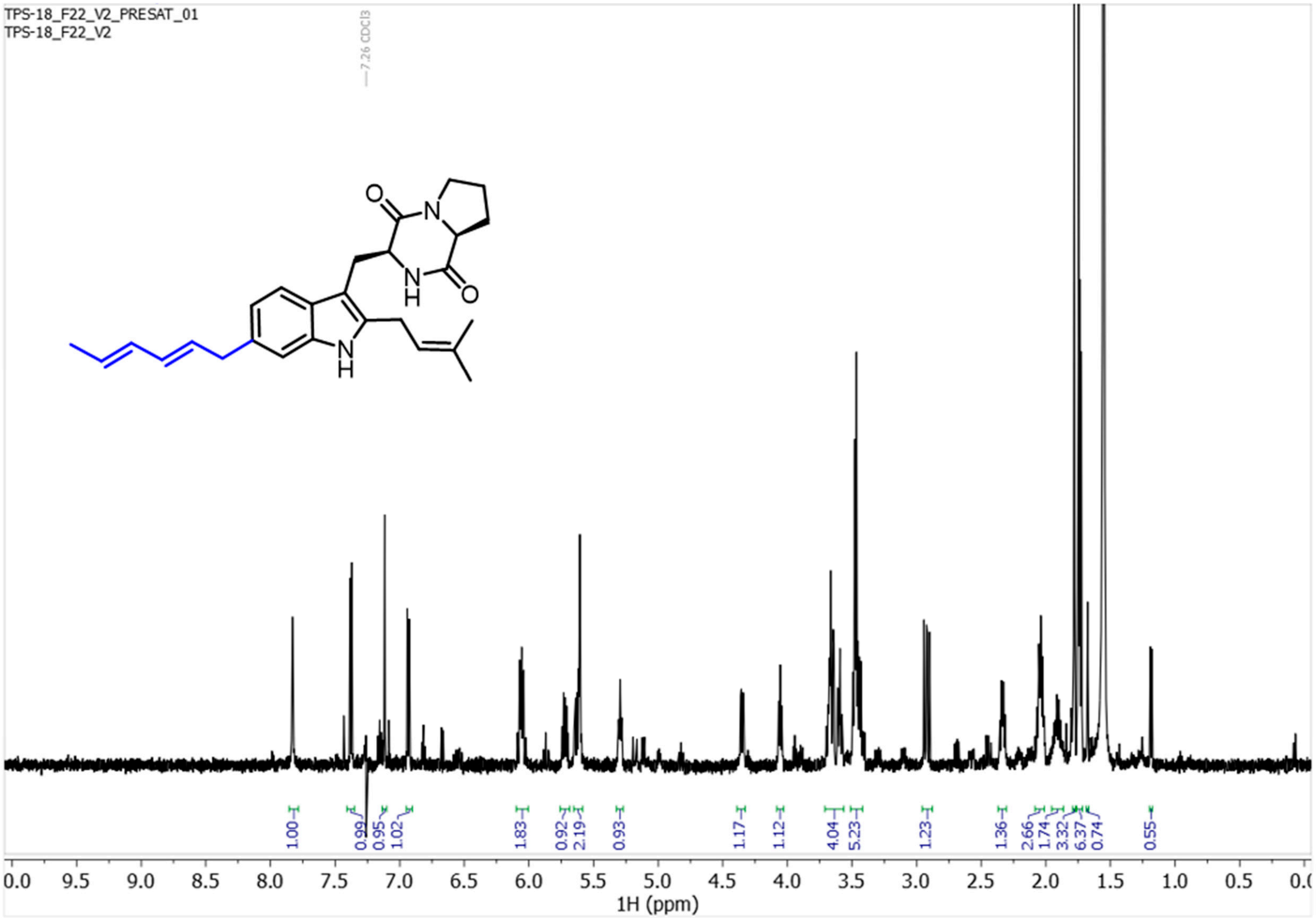

^{1}H PRESAT NMR spectrum (500 MHz) of **TPS-16** in $CDCl_3$

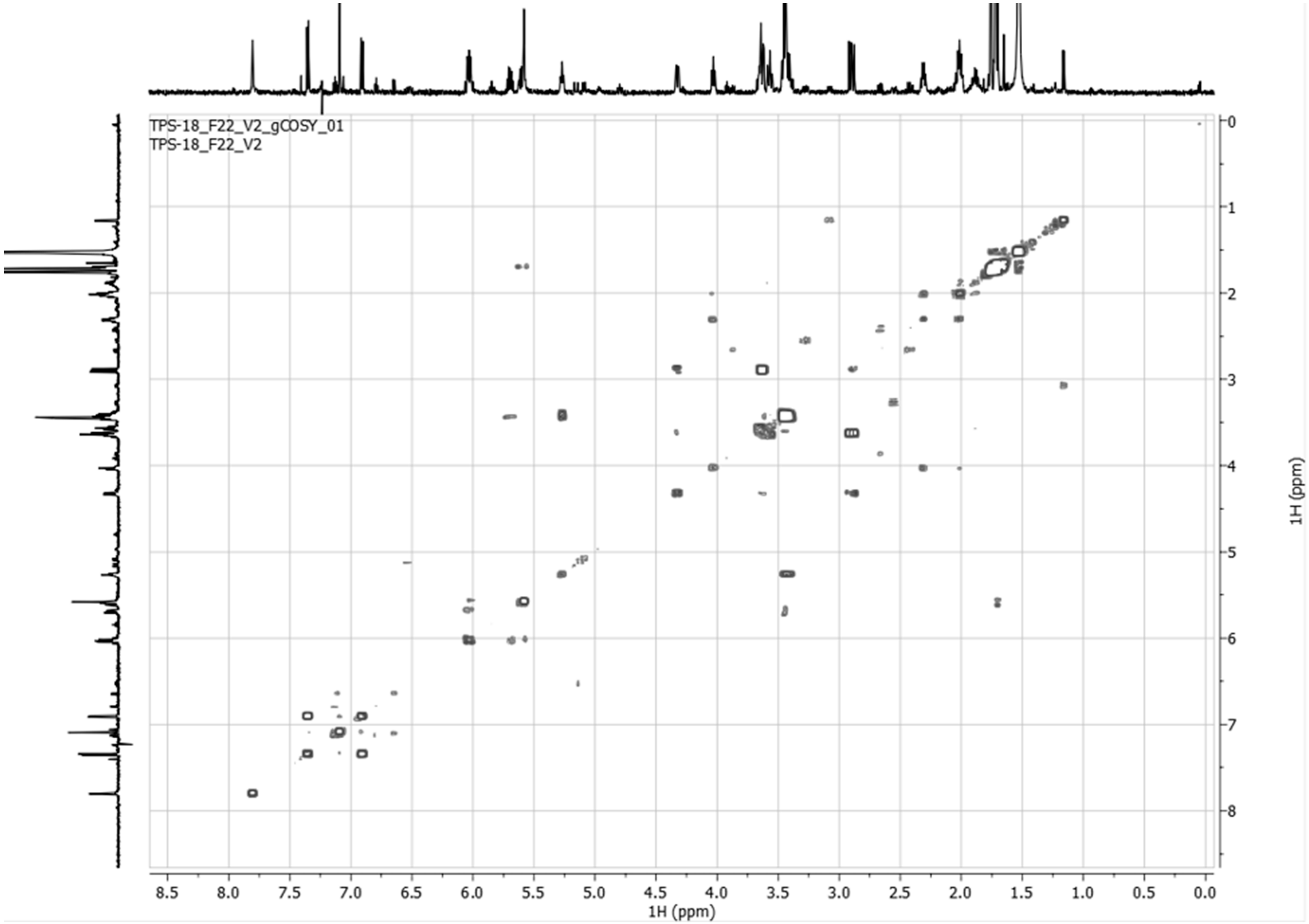

COSY NMR spectrum (500 MHz) of **TPS-16** in $CDCl_3$

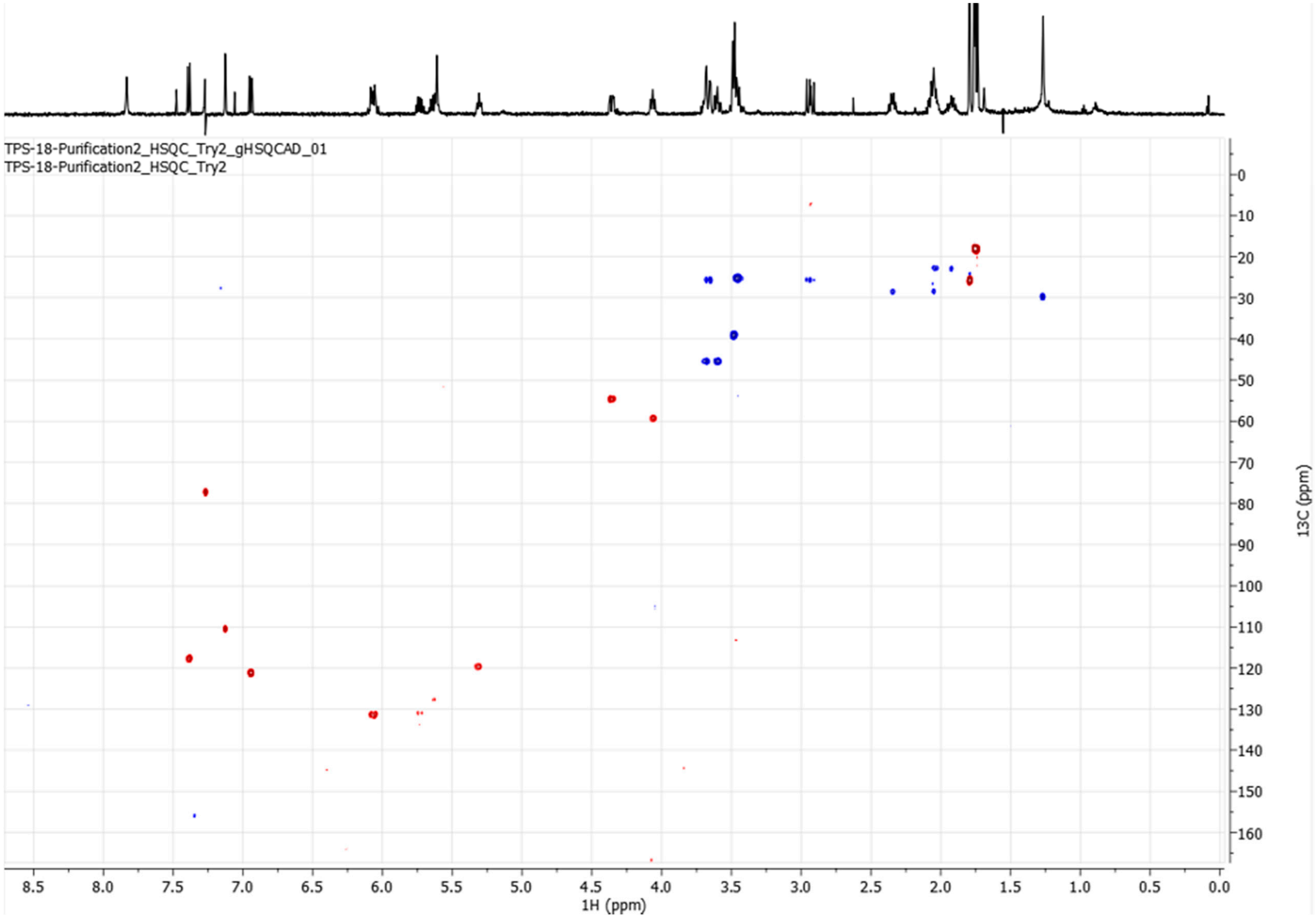

HSQC NMR spectrum (500 MHz) of **TPS-16** in $CDCl_3$

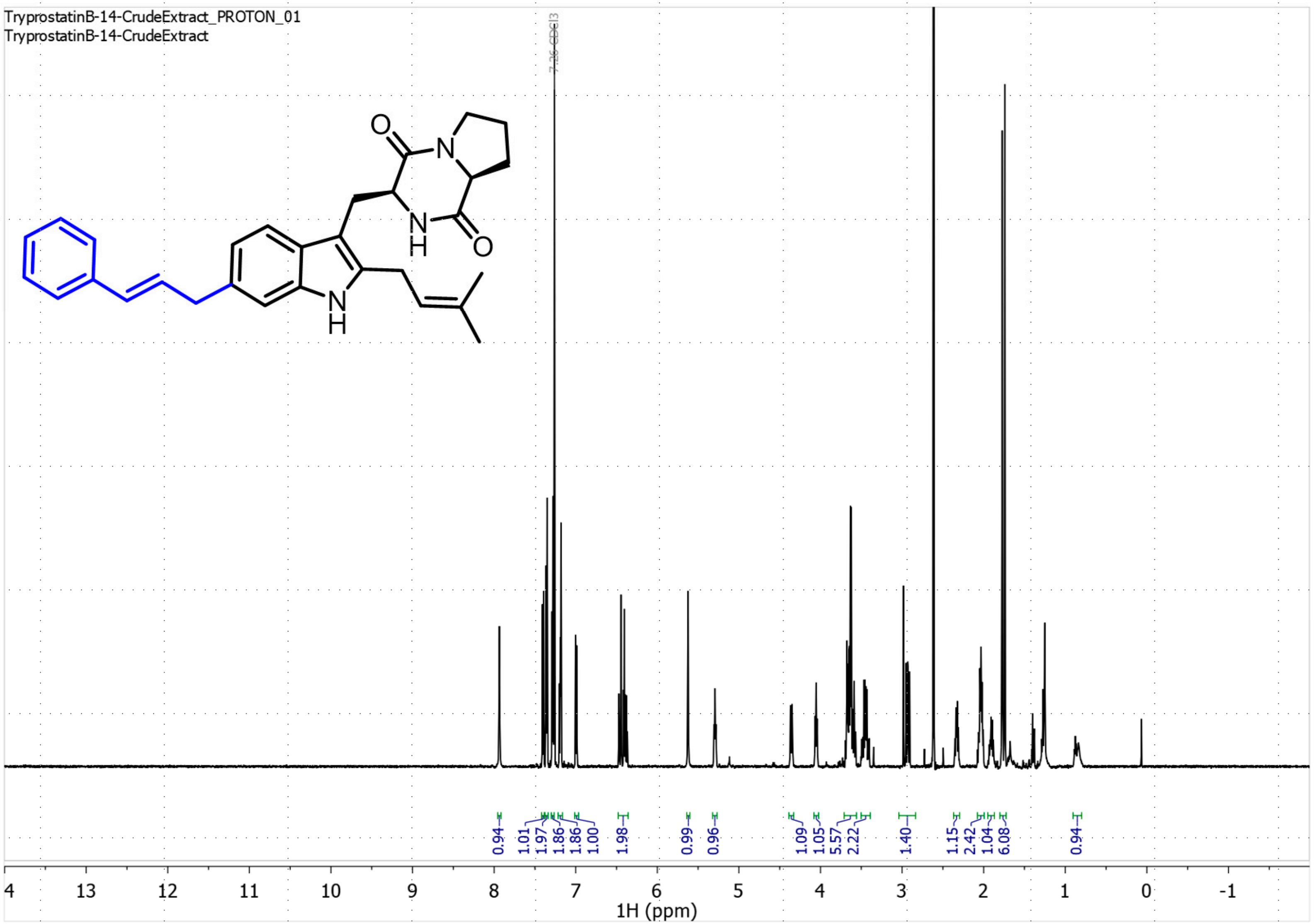

^{1}H NMR spectrum (600 MHz) of **TPS-24** in $CDCl_3$

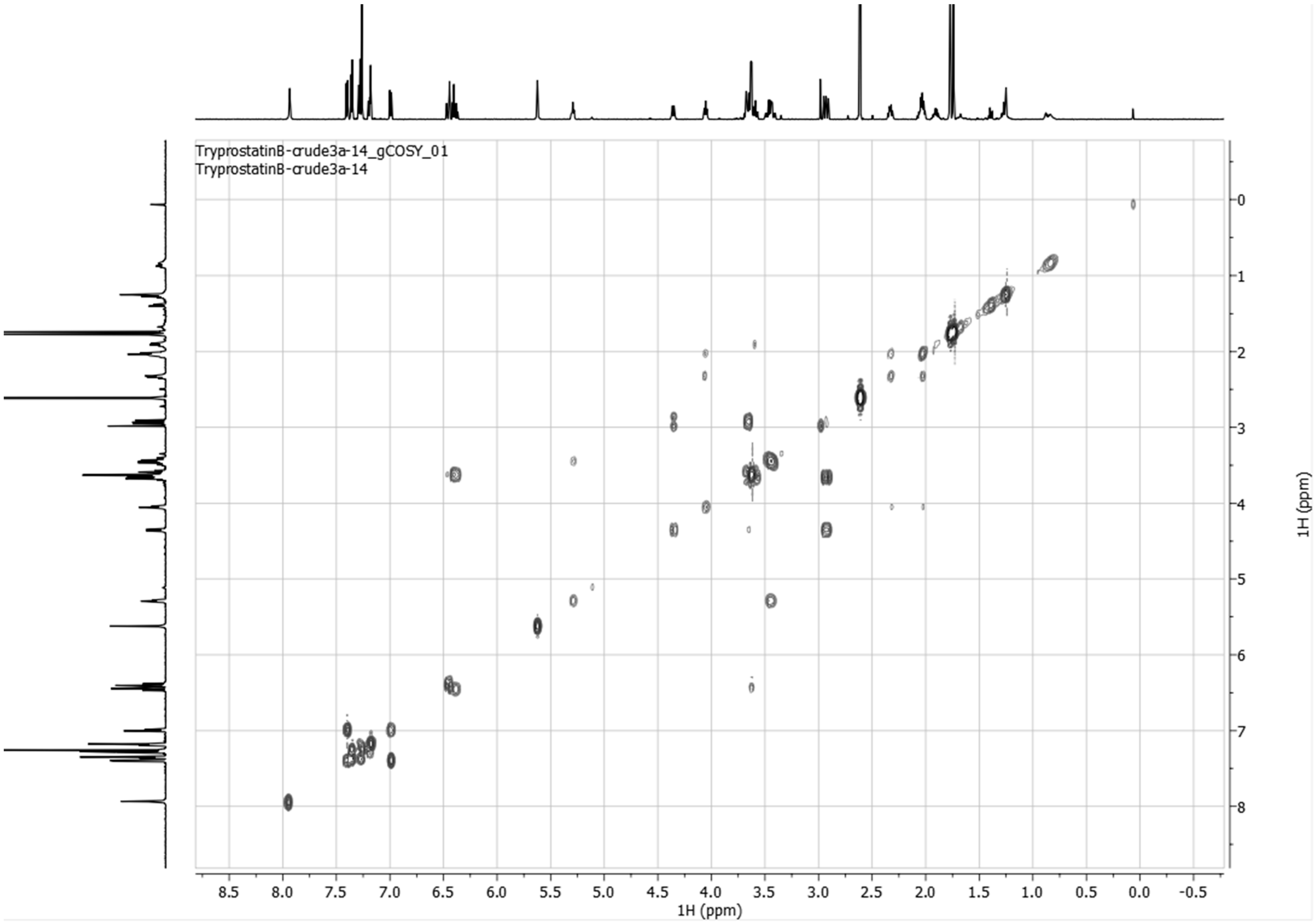

COSY NMR spectrum (600 MHz) of **TPS-24** in $CDCl_3$

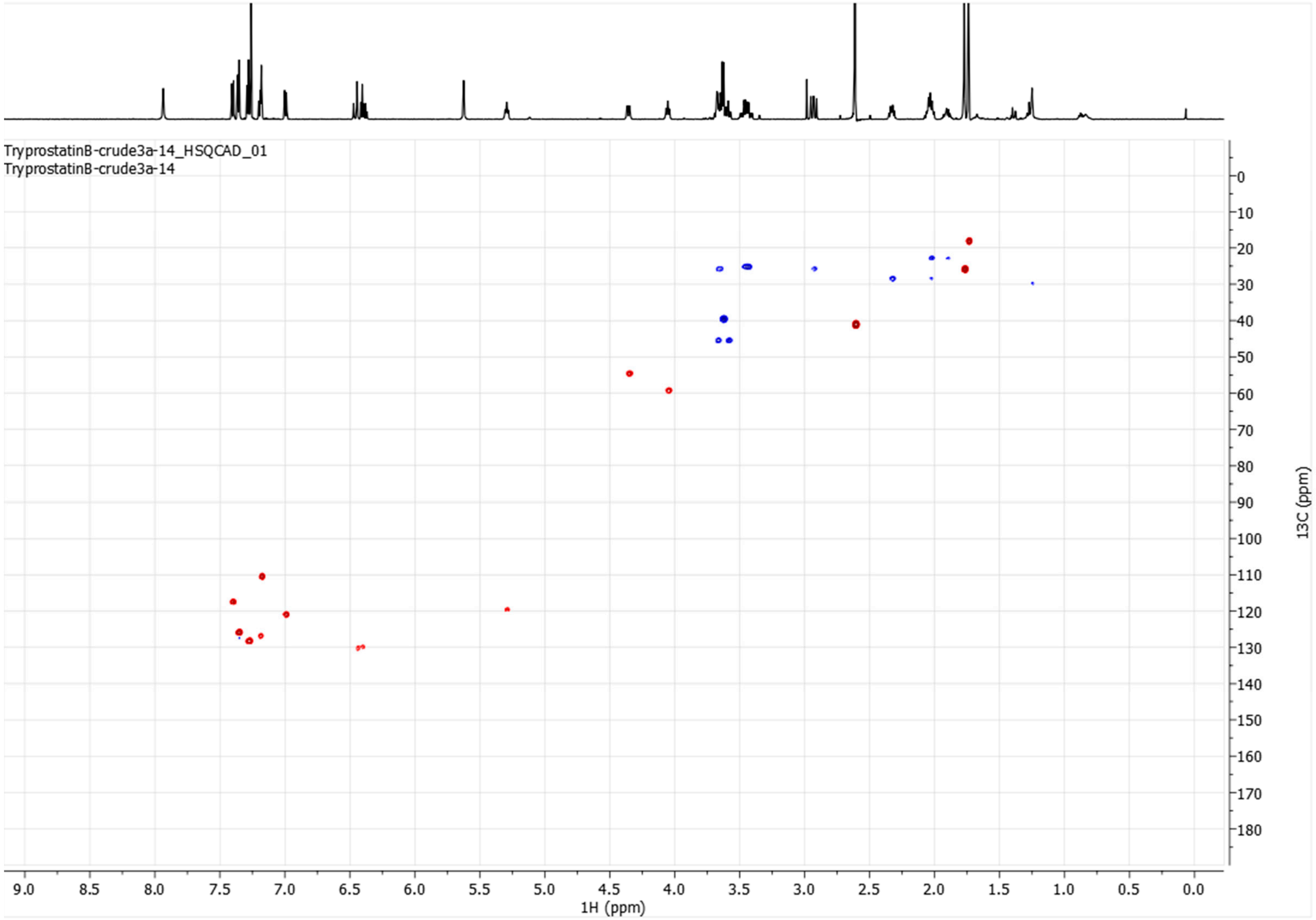

HSQC NMR spectrum (600 MHz) of **TPS-24** in $CDCl_3$

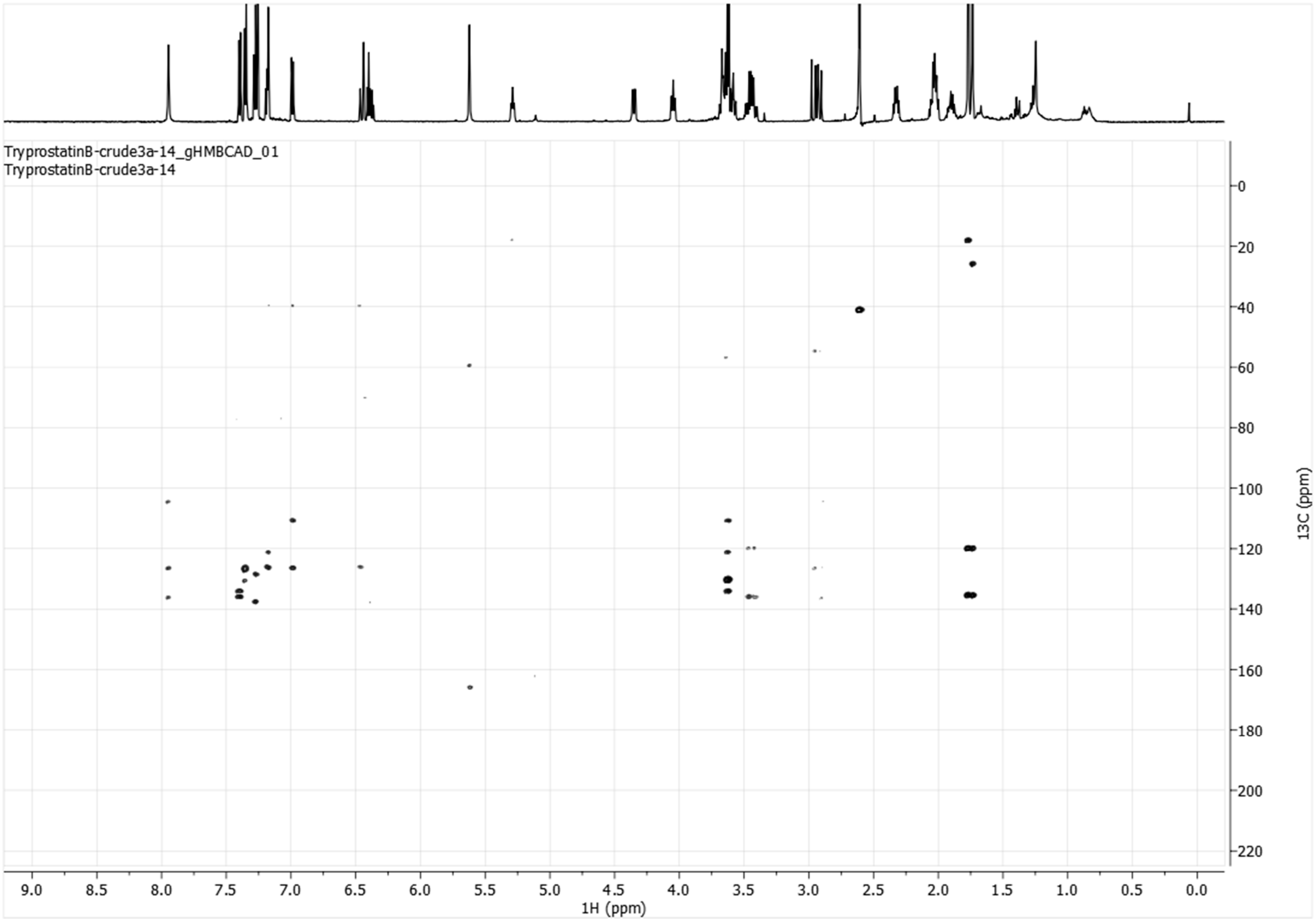

HMBC NMR spectrum (600 MHz) of **TPS-24** in $CDCl_3$

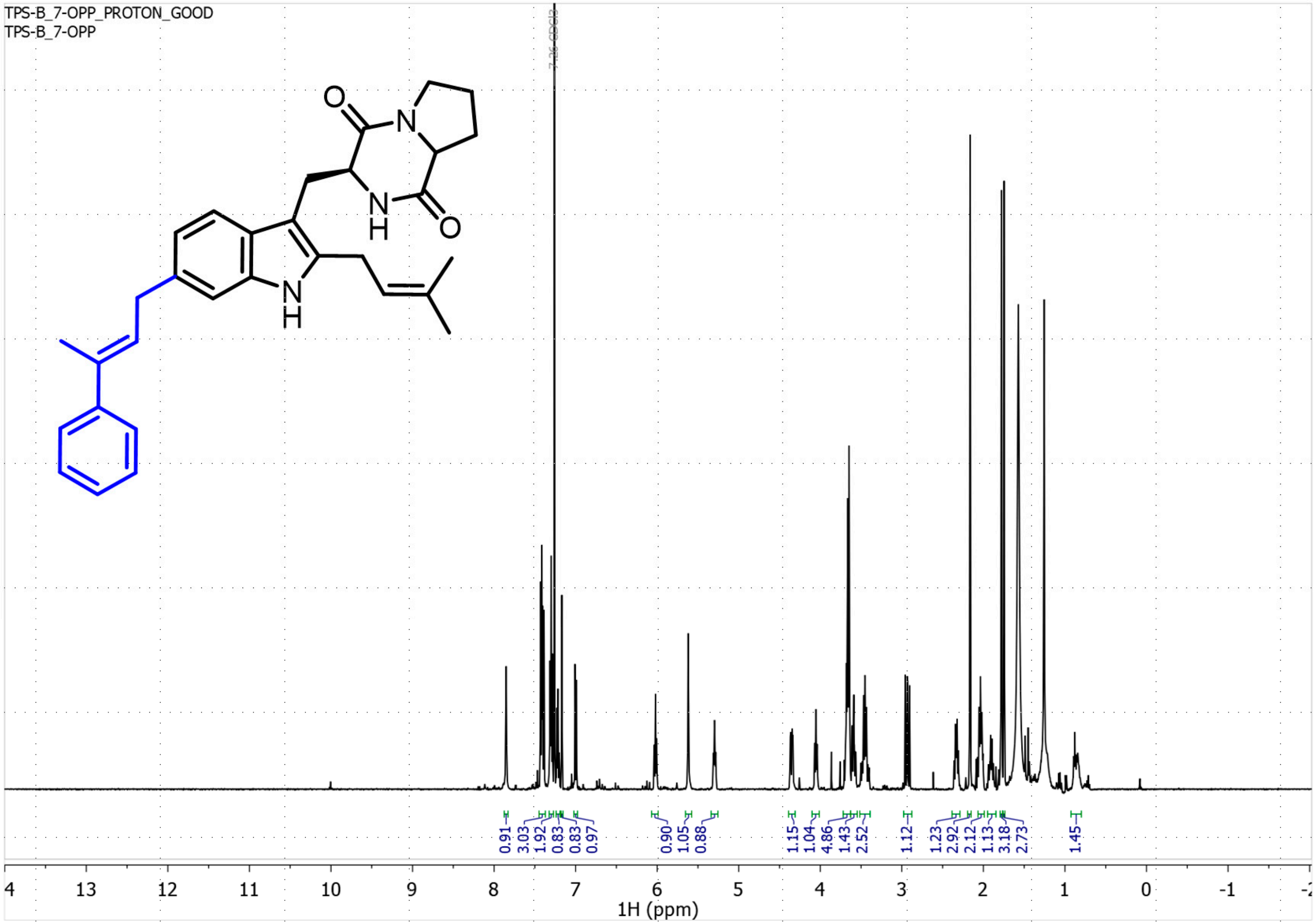

^{1}H NMR spectrum (500 MHz) of **TPS-25** in $CDCl_3$

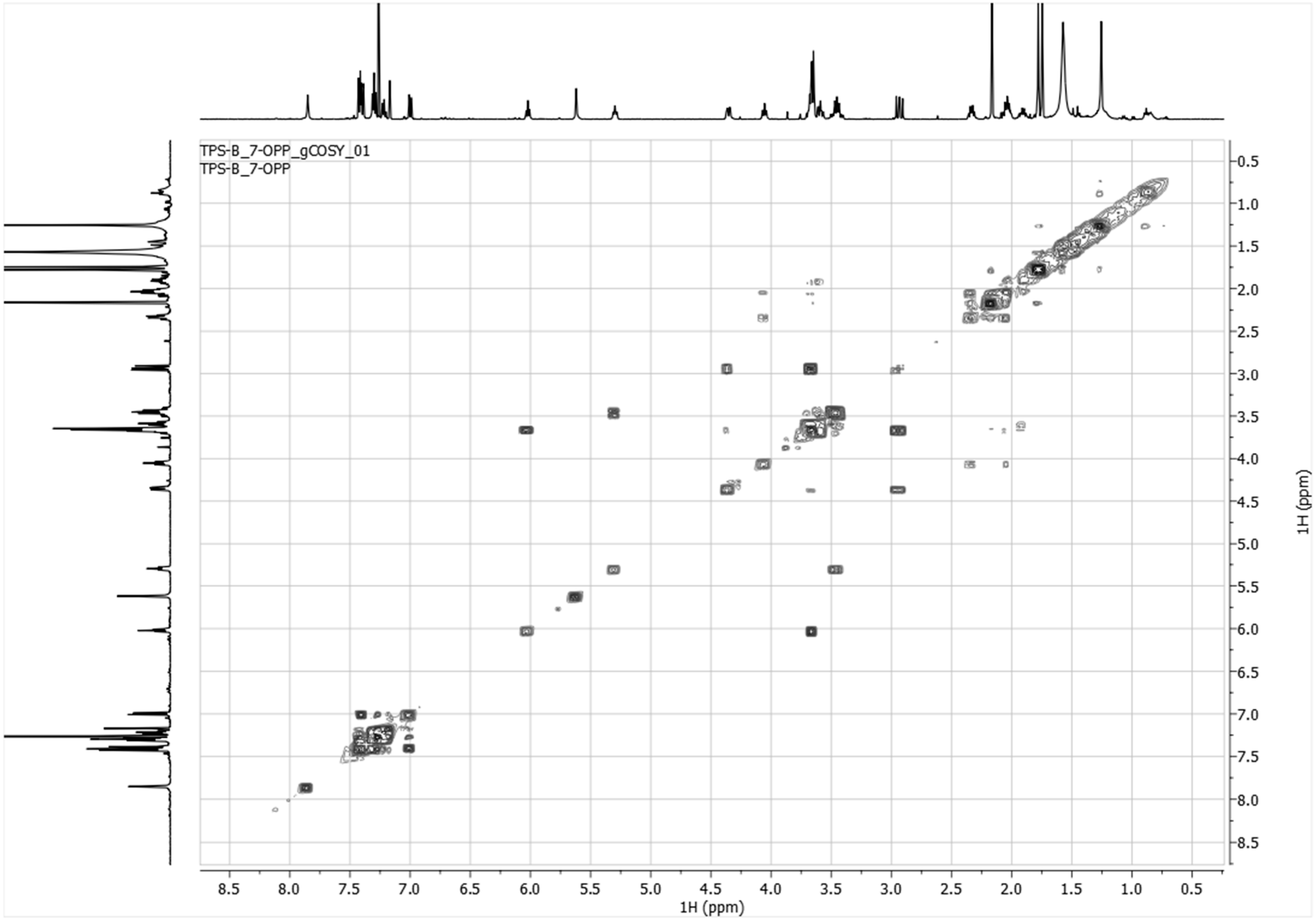

COSY NMR spectrum (500 MHz) of **TPS-25** in $CDCl_3$

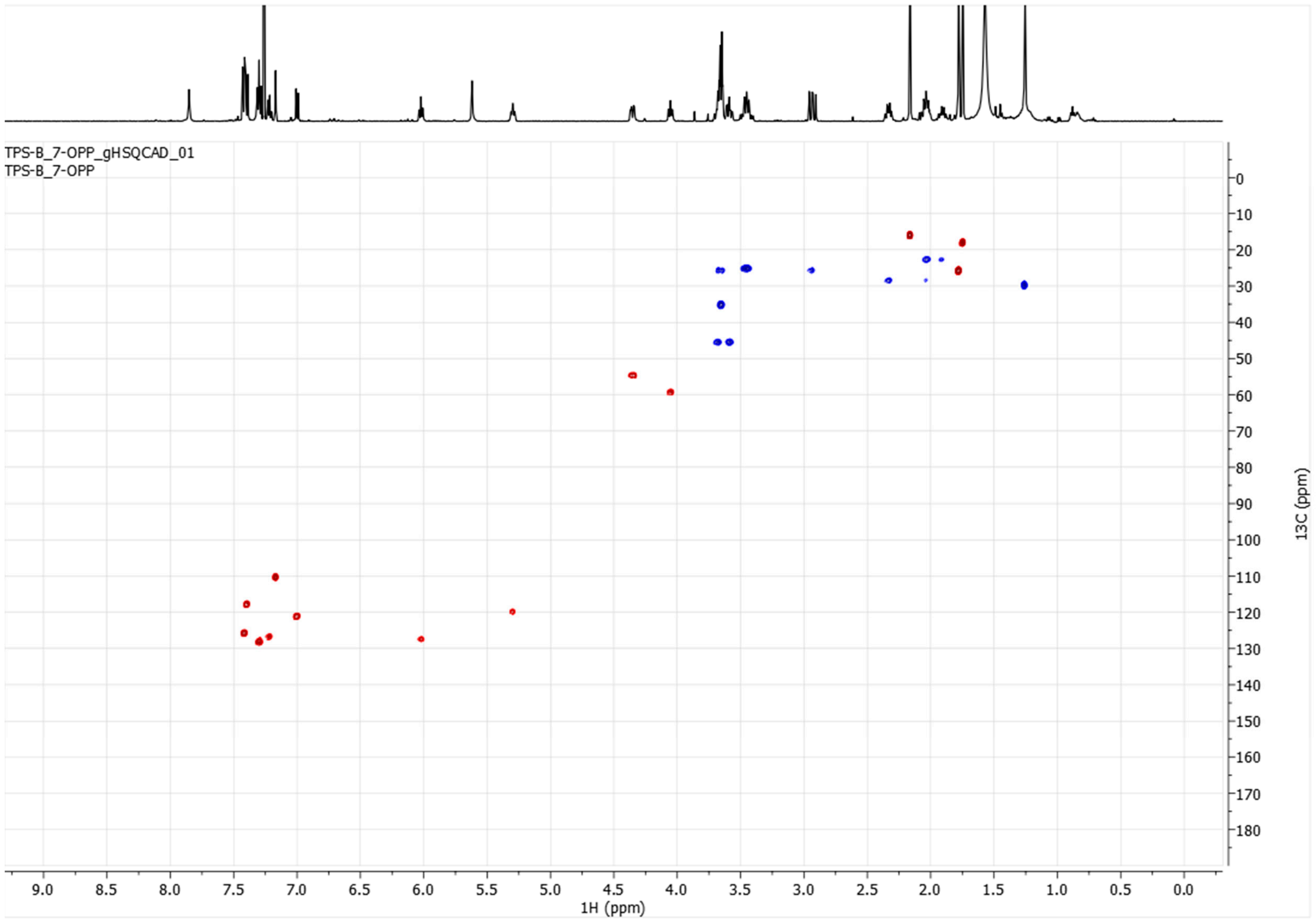

HSQC NMR spectrum (500 MHz) of **TPS-25** in $CDCl_3$

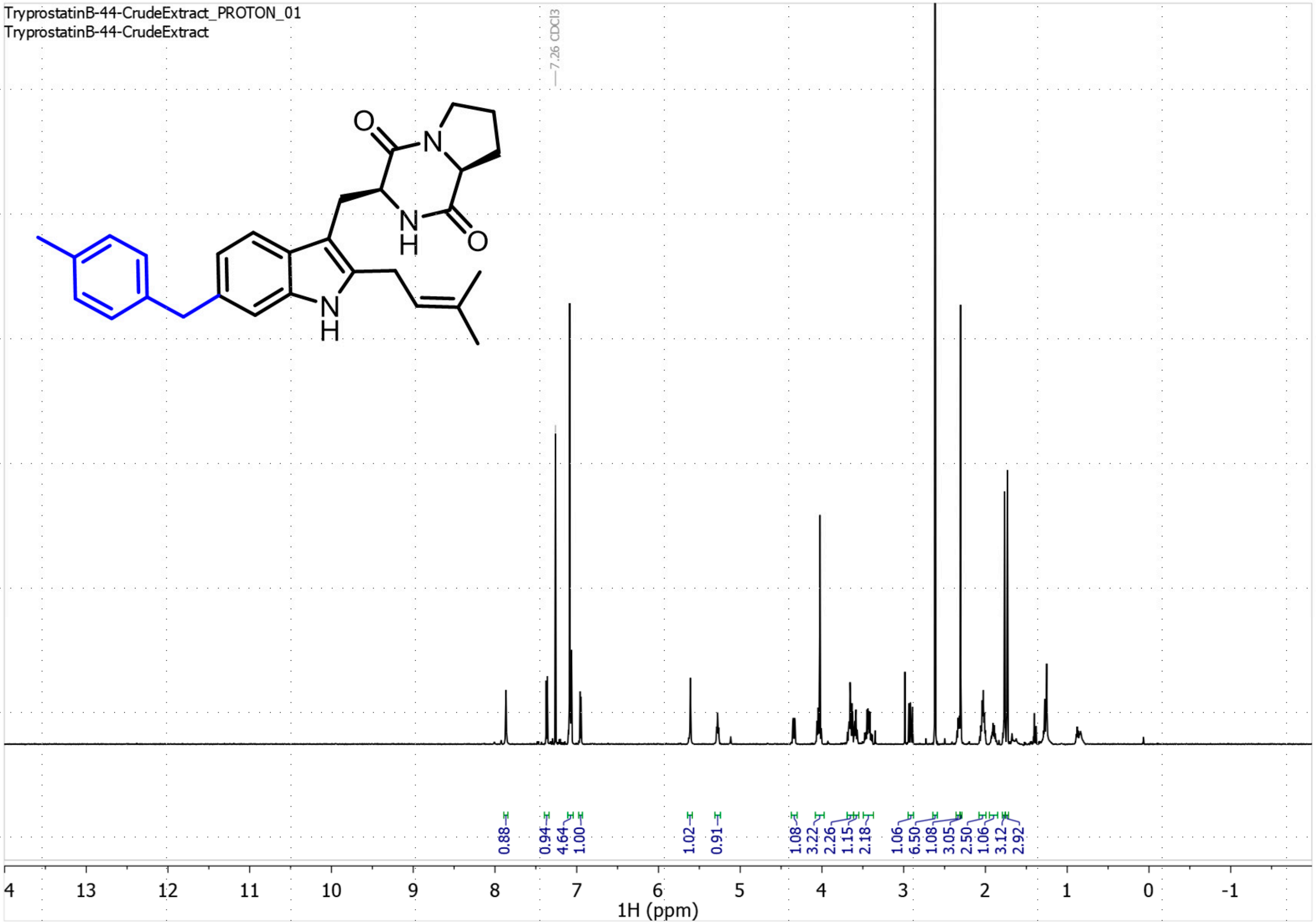

^{1}H NMR spectrum (600 MHz) of **TPS-46** in $CDCl_3$

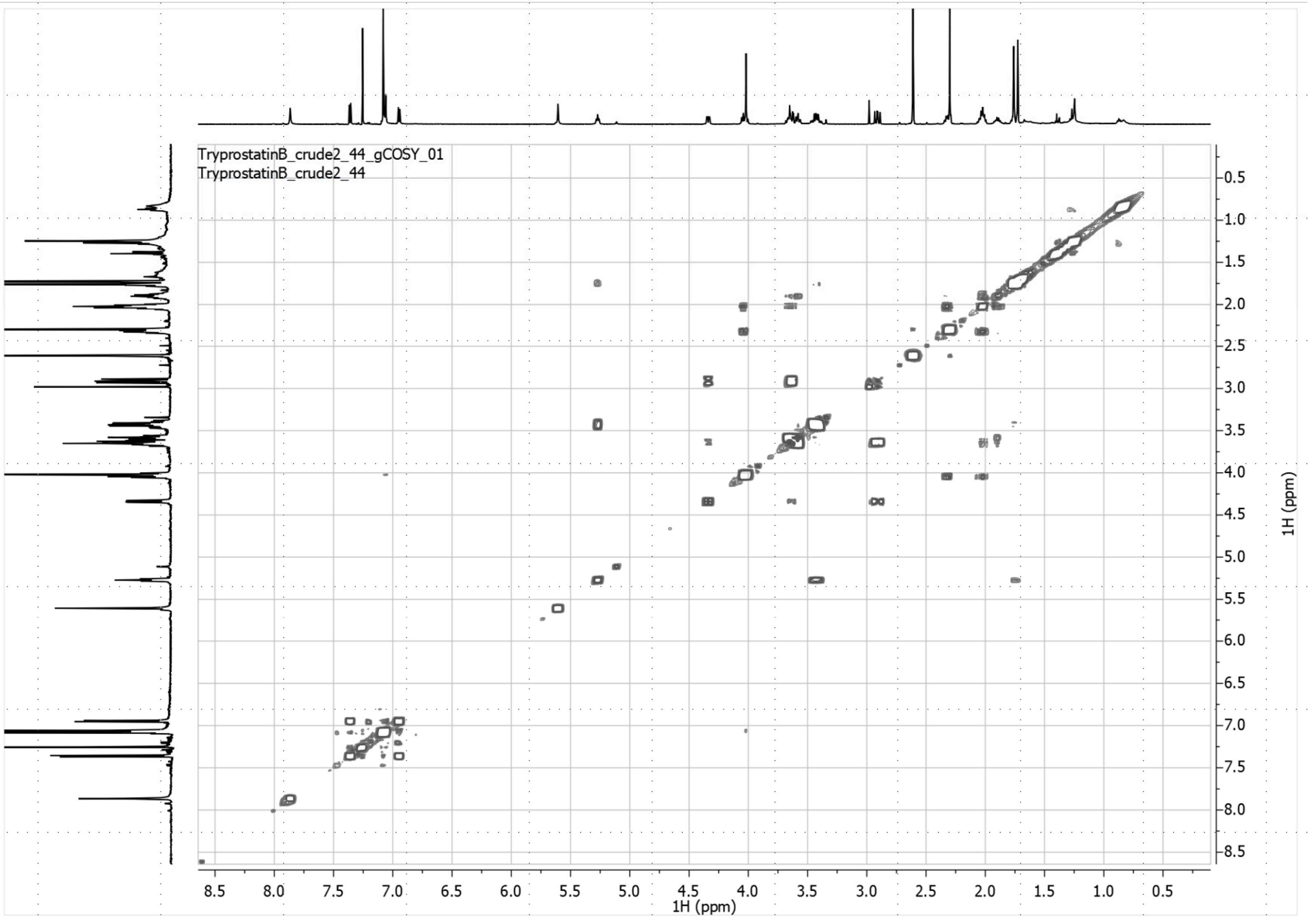

COSY NMR spectrum (600 MHz) of **TPS-46** in $CDCl_3$

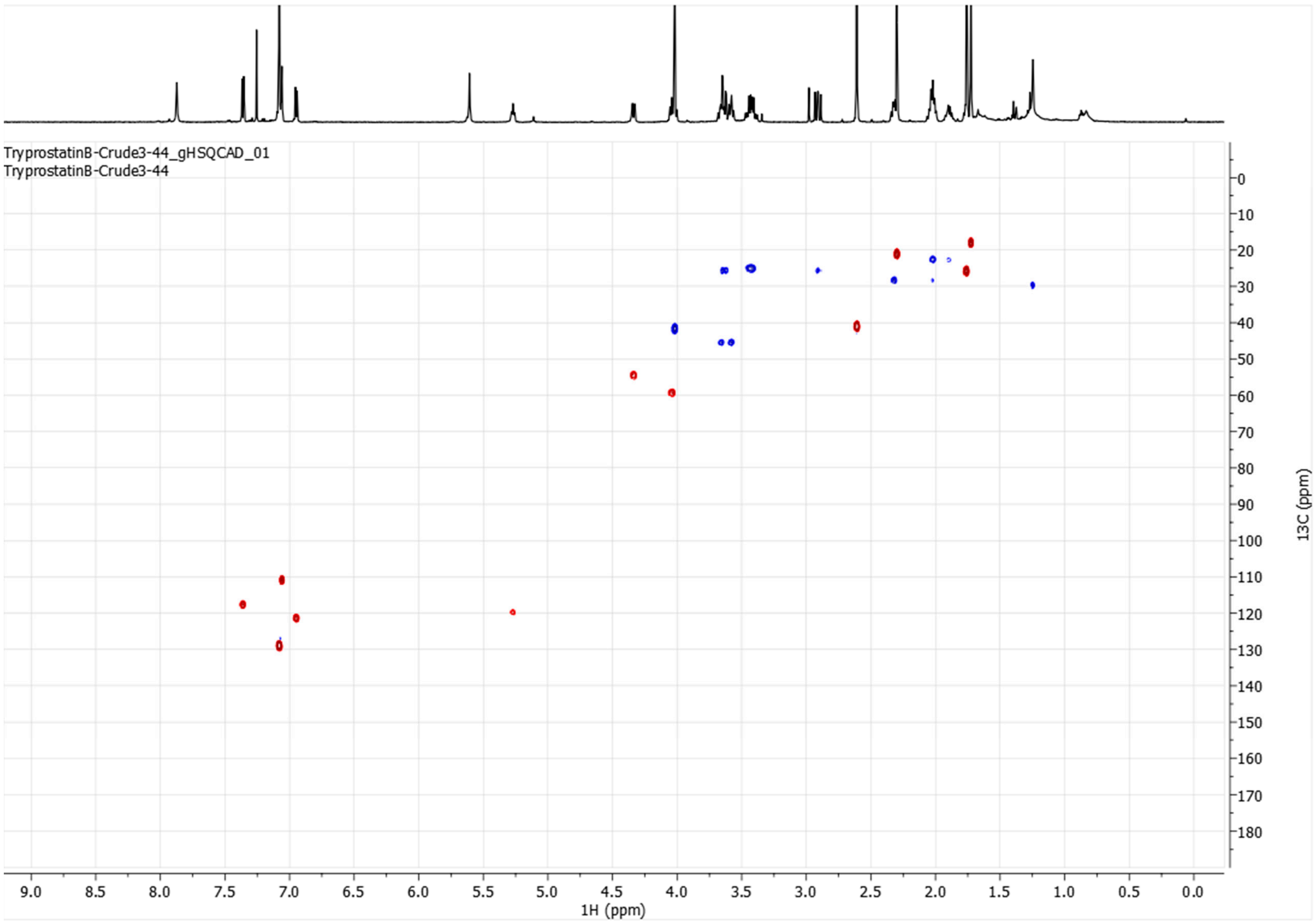

HSQC NMR spectrum (600 MHz) of **TPS-46** in $CDCl_3$

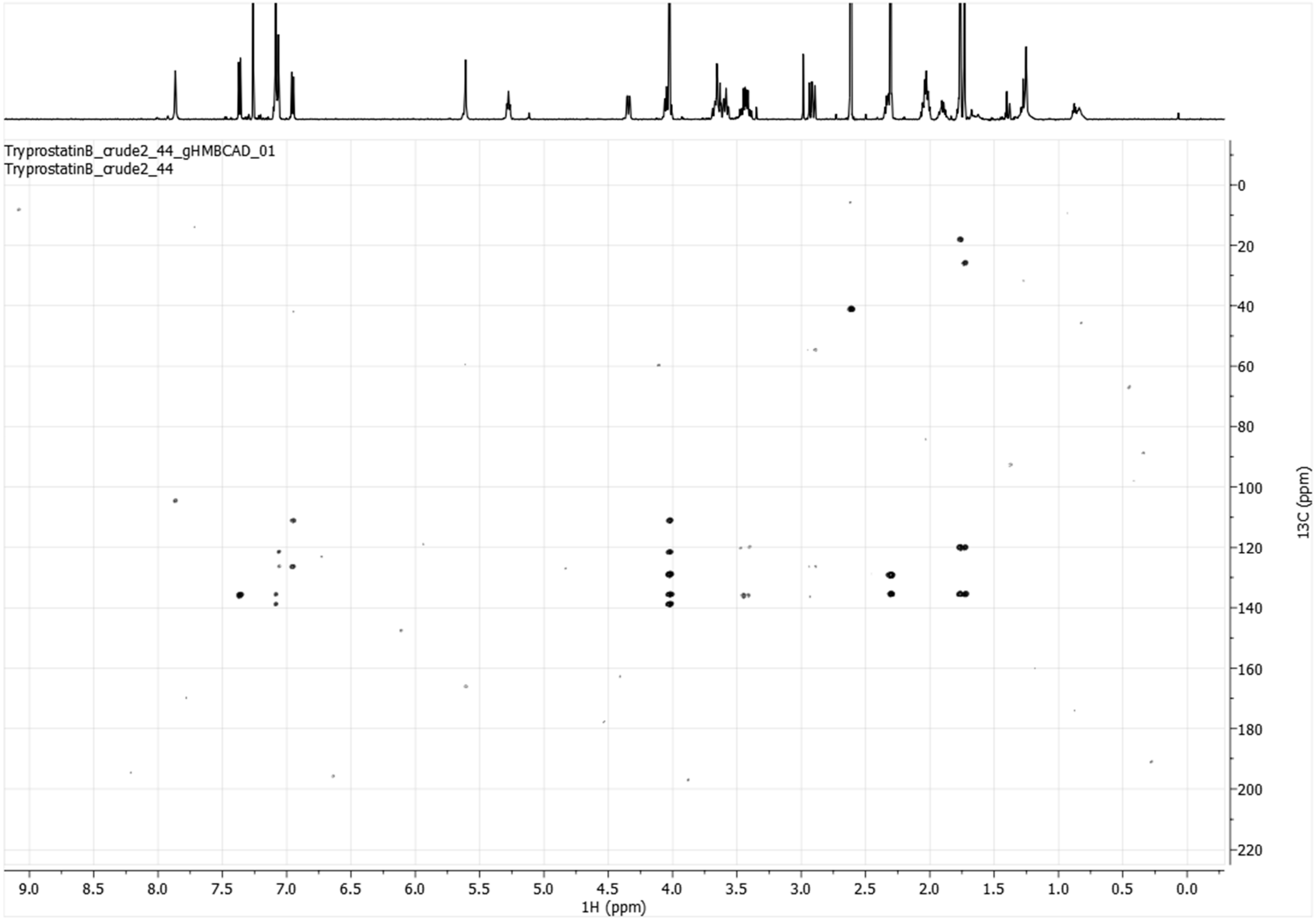

HMBC NMR spectrum (600 MHz) of **TPS-46** in $CDCl_3$

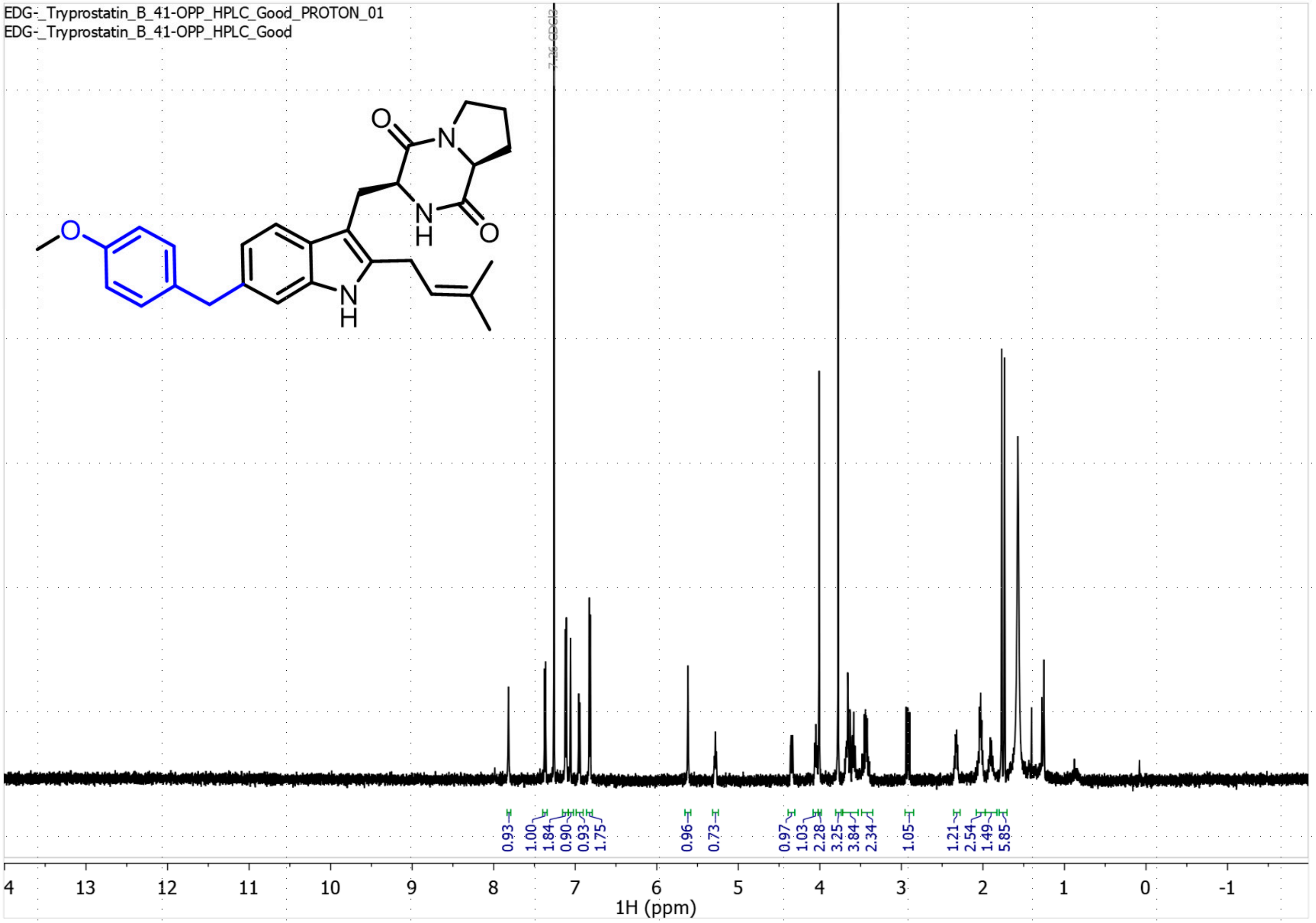

^{1}H NMR spectrum (600 MHz) of **TPS-52** in $CDCl_3$

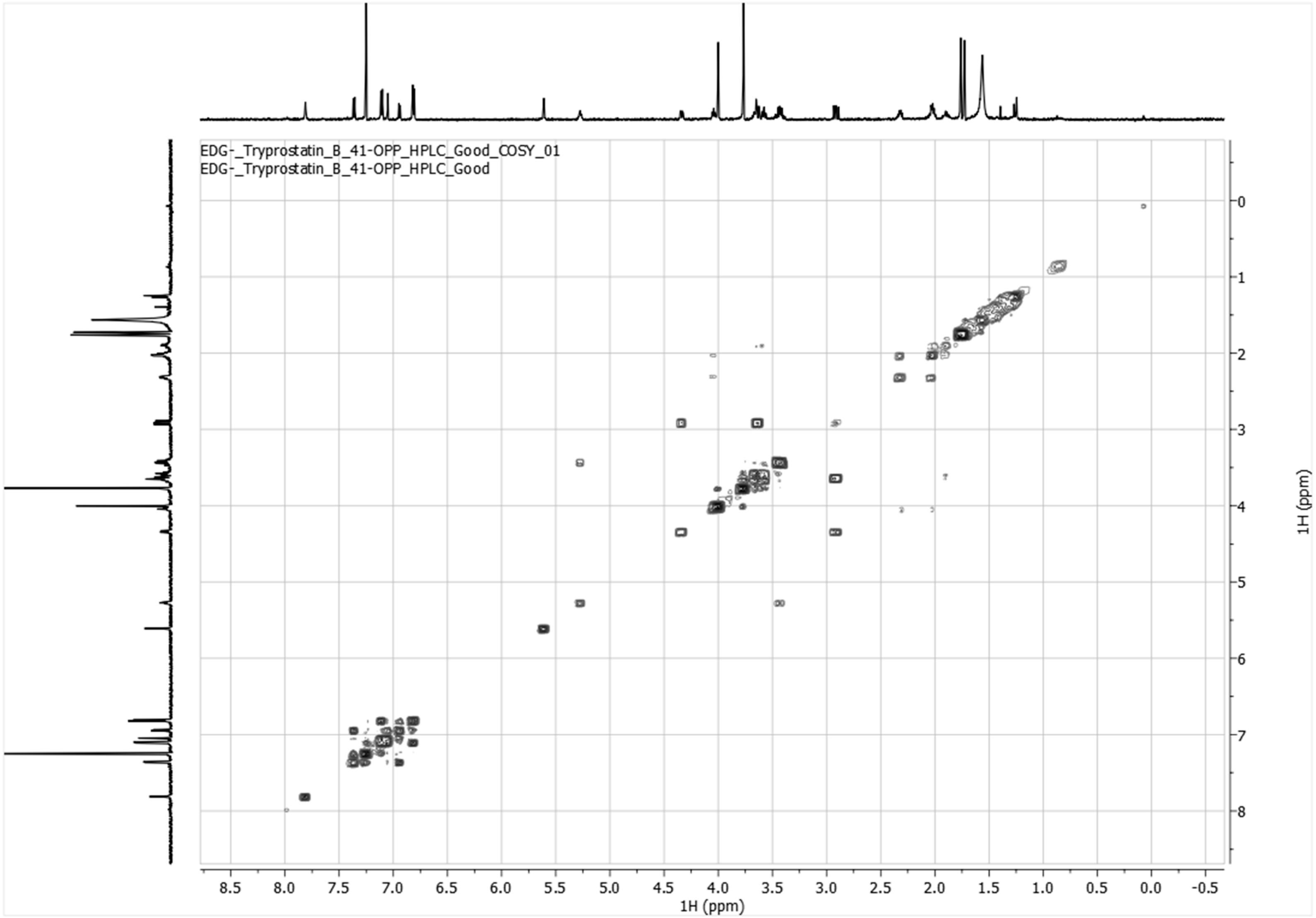

COSY NMR spectrum (600 MHz) of **TPS-52** in $CDCl_3$

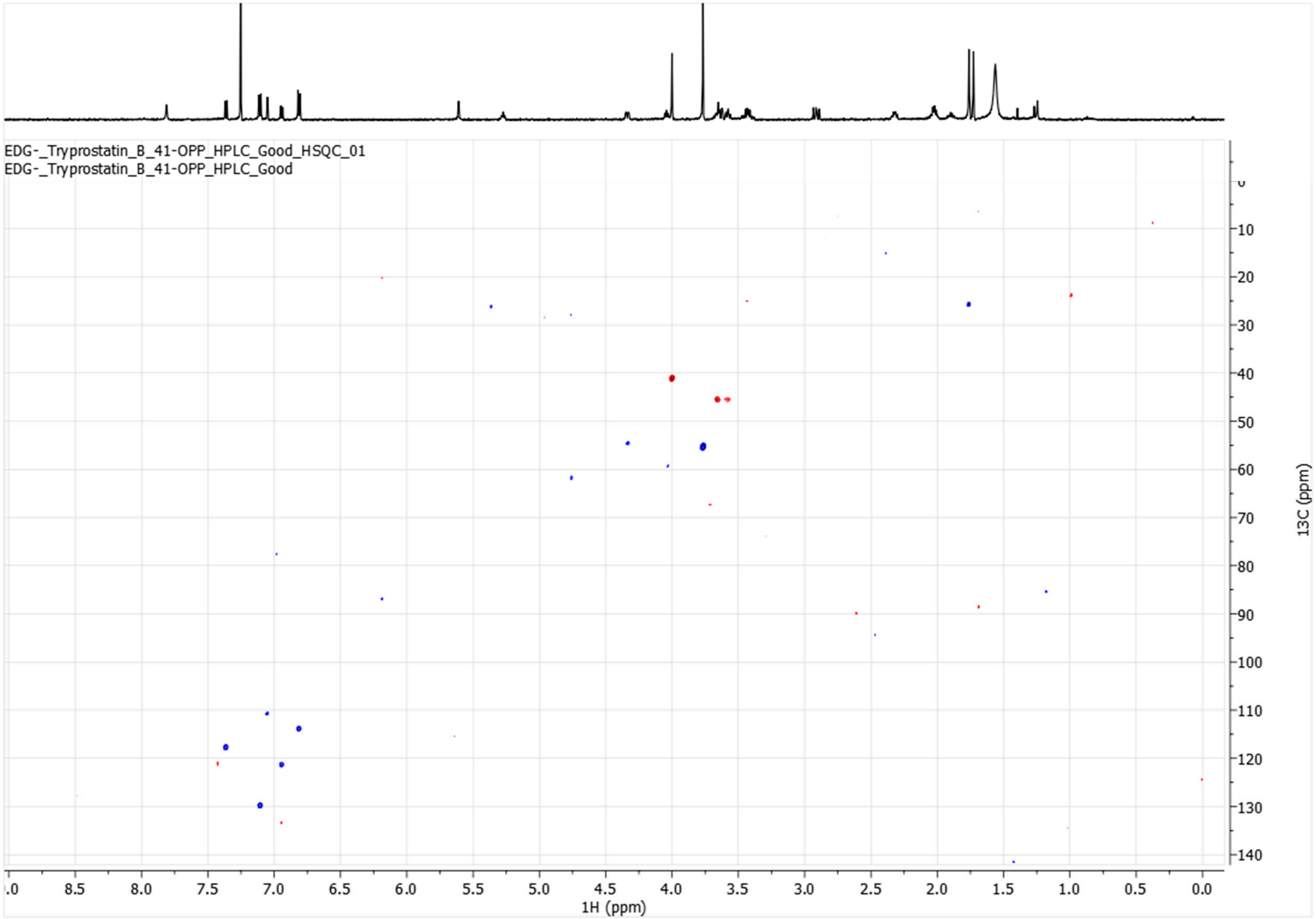

HSQC NMR spectrum (600 MHz) of **TPS-52** in $CDCl_3$

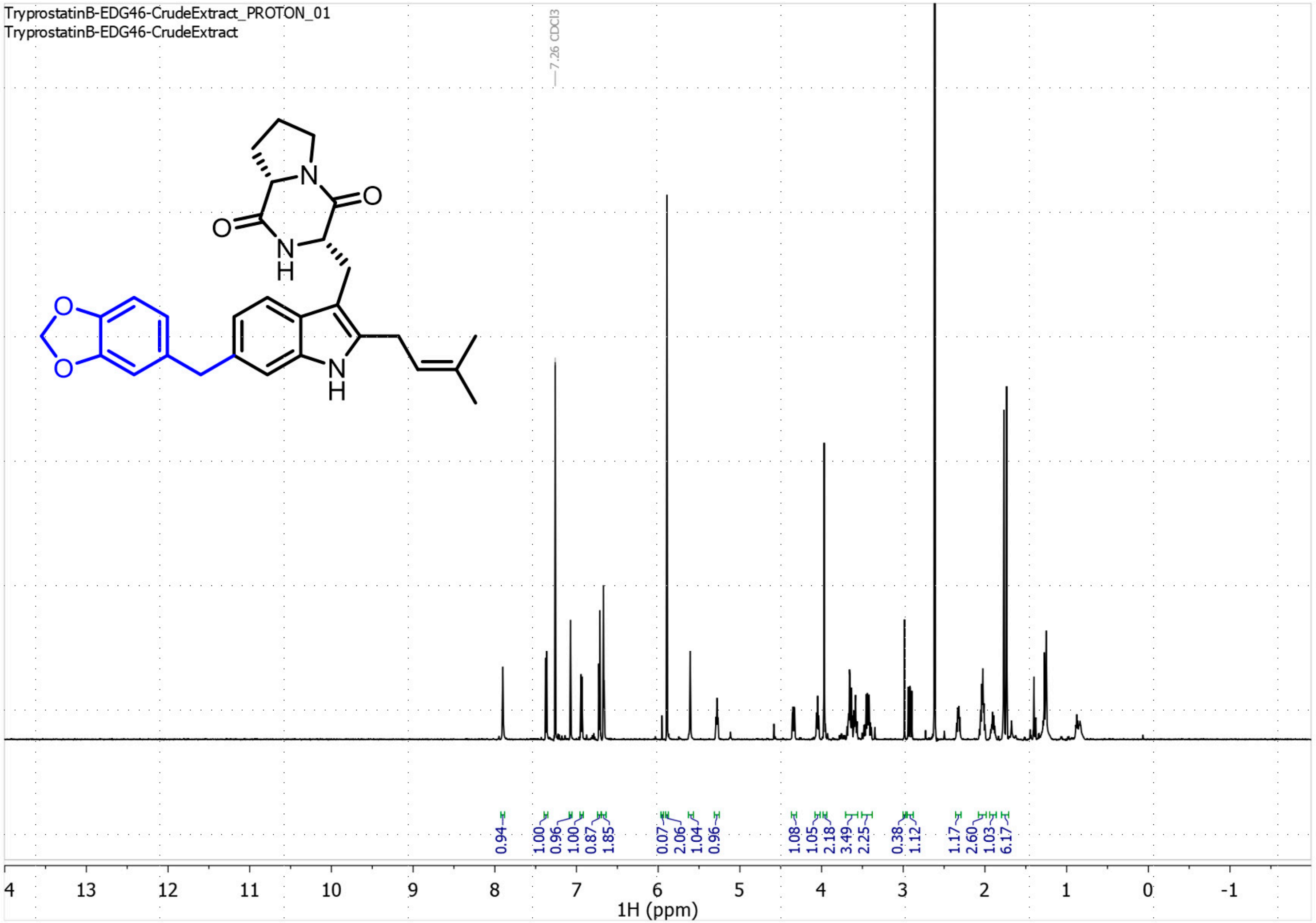

^{1}H NMR Spectrum (600 MHz) of **TPS-58** in $CDCl_3$

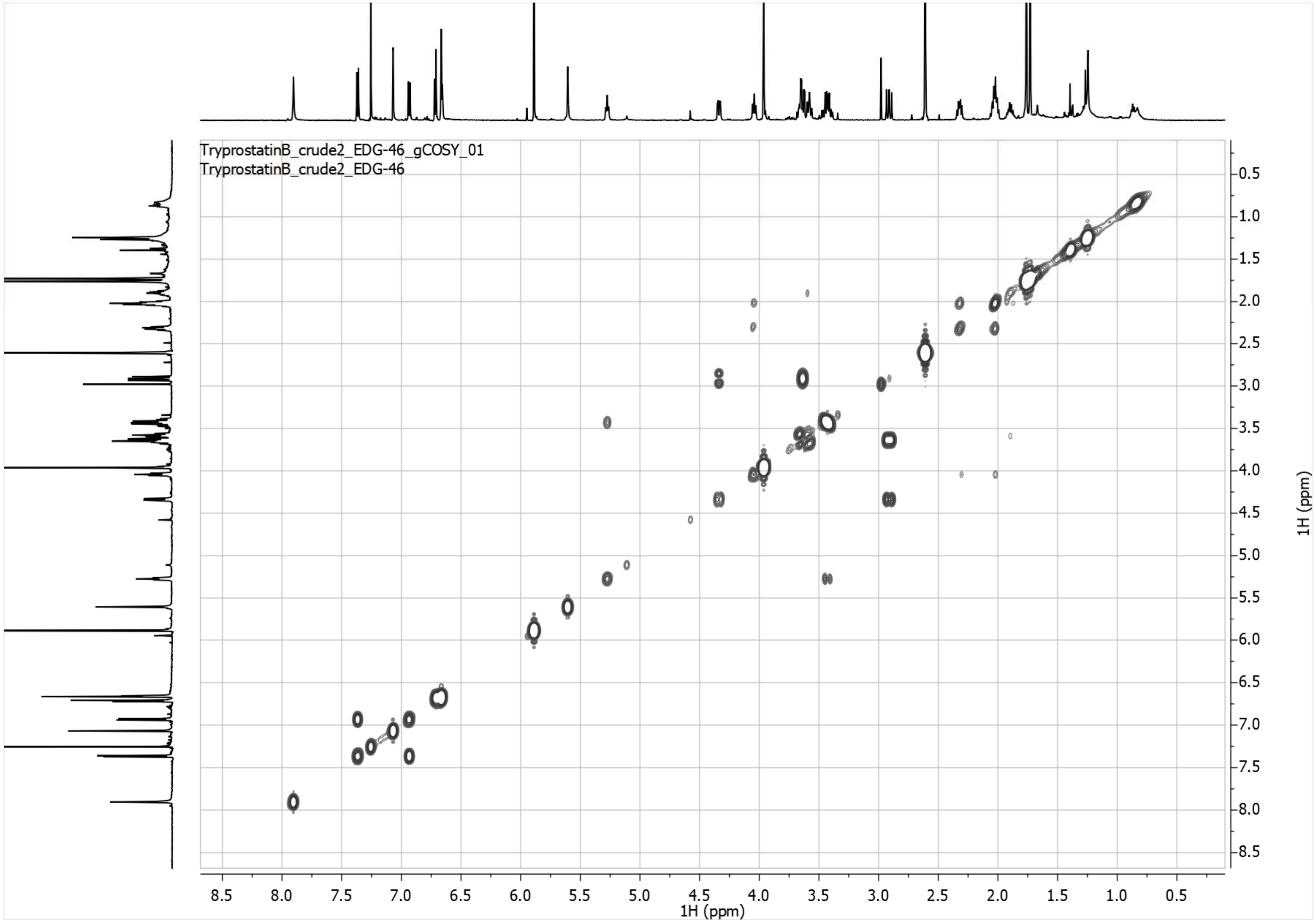

COSY NMR spectrum (600 MHz) of **TPS-58** in $CDCl_3$

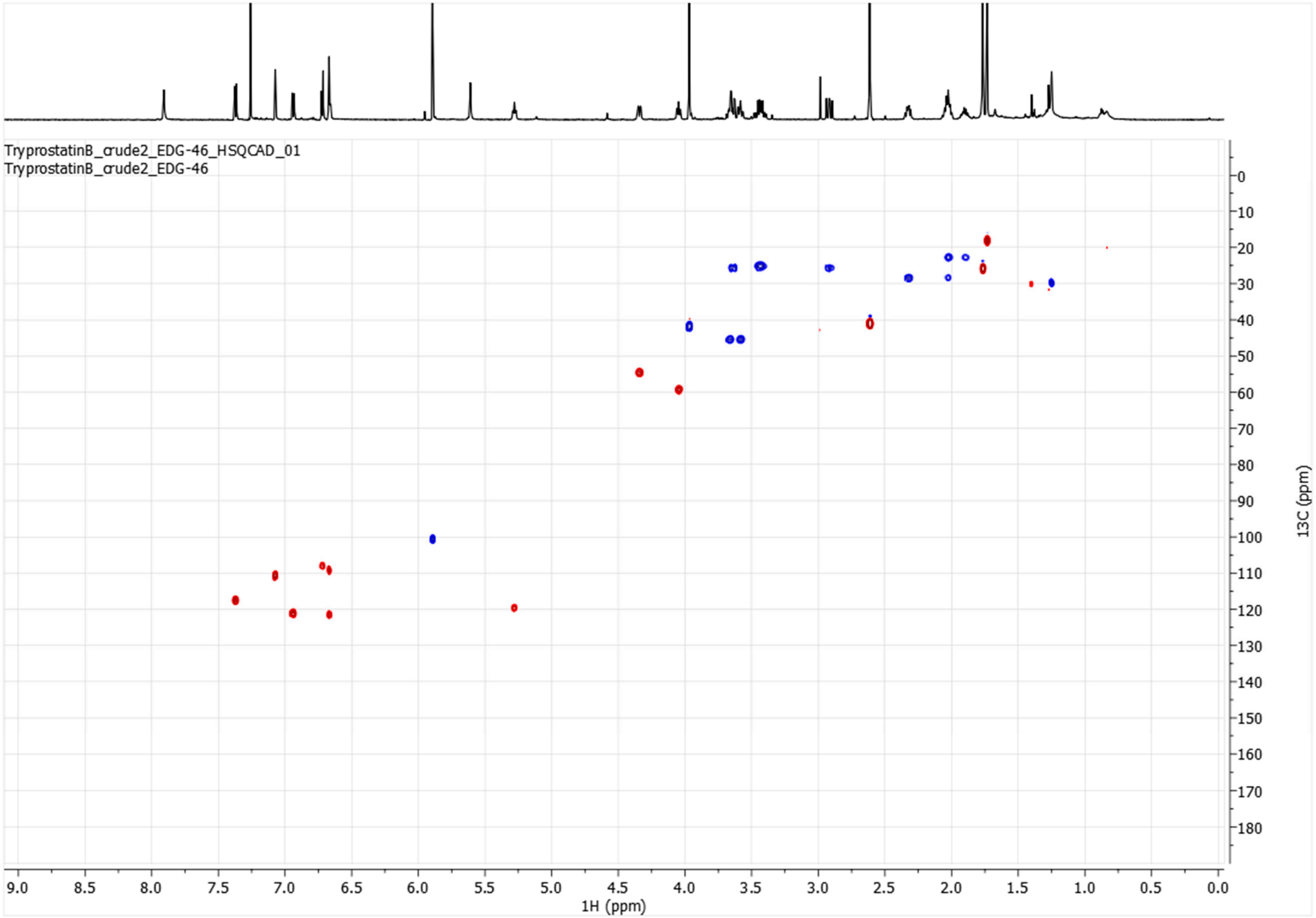

HSQC NMR spectrum (600 MHz) of **TPS-58** in $CDCl_3$

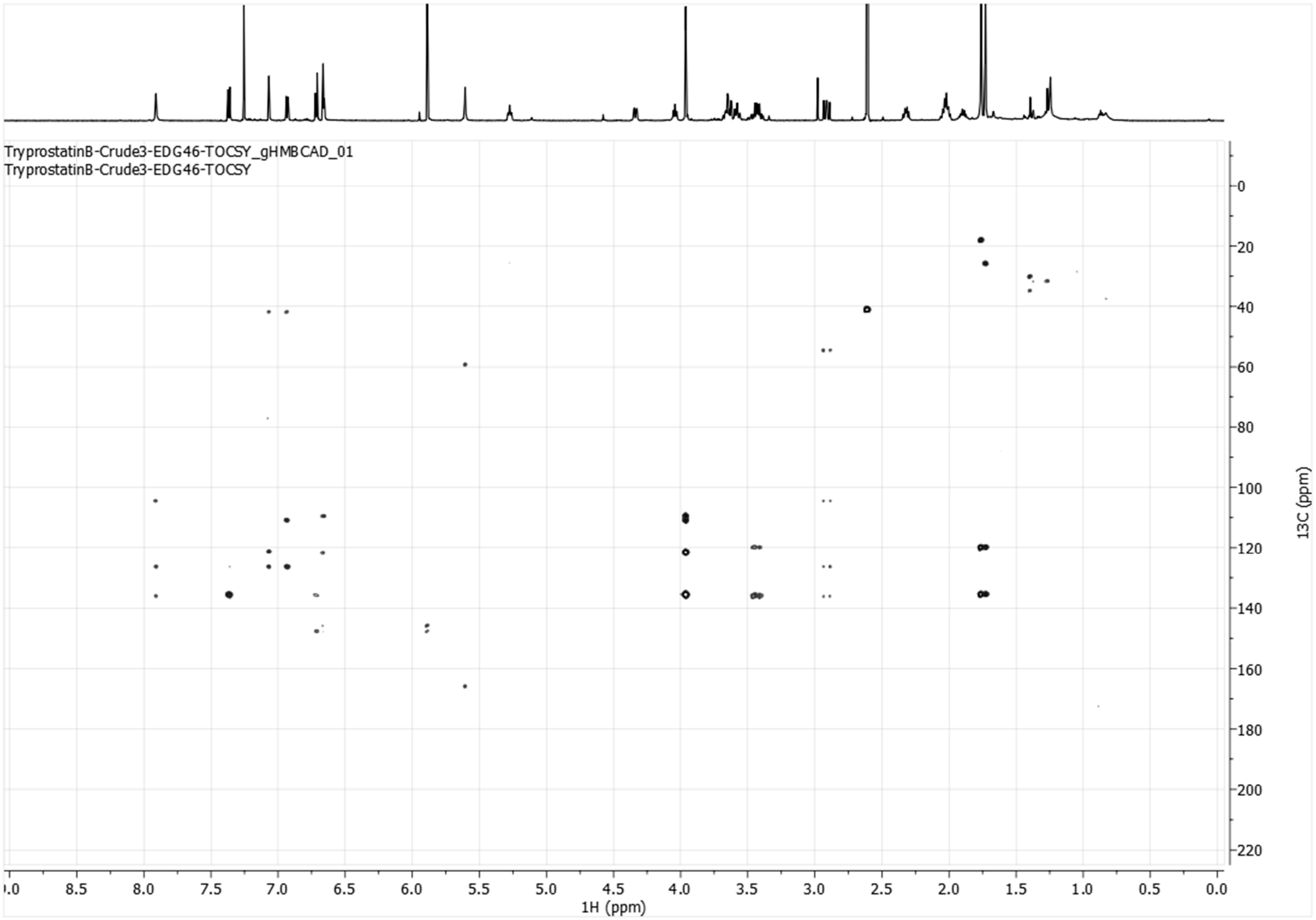

HMBC NMR spectrum (600 MHz) of **TPS-58** in $CDCl_3$

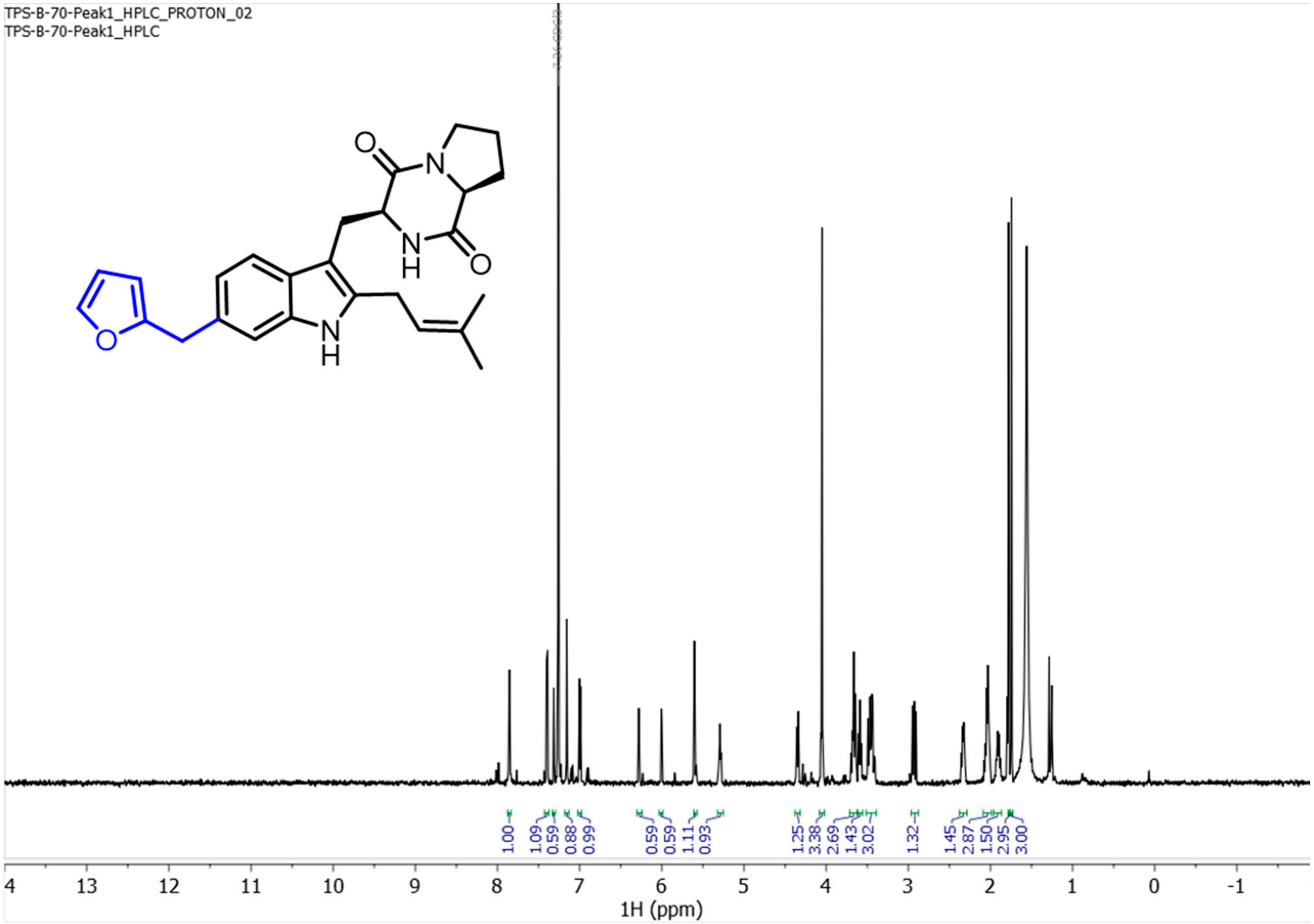

^{1}H NMR spectrum (500 MHz) of **TPS-61** in $CDCl_3$

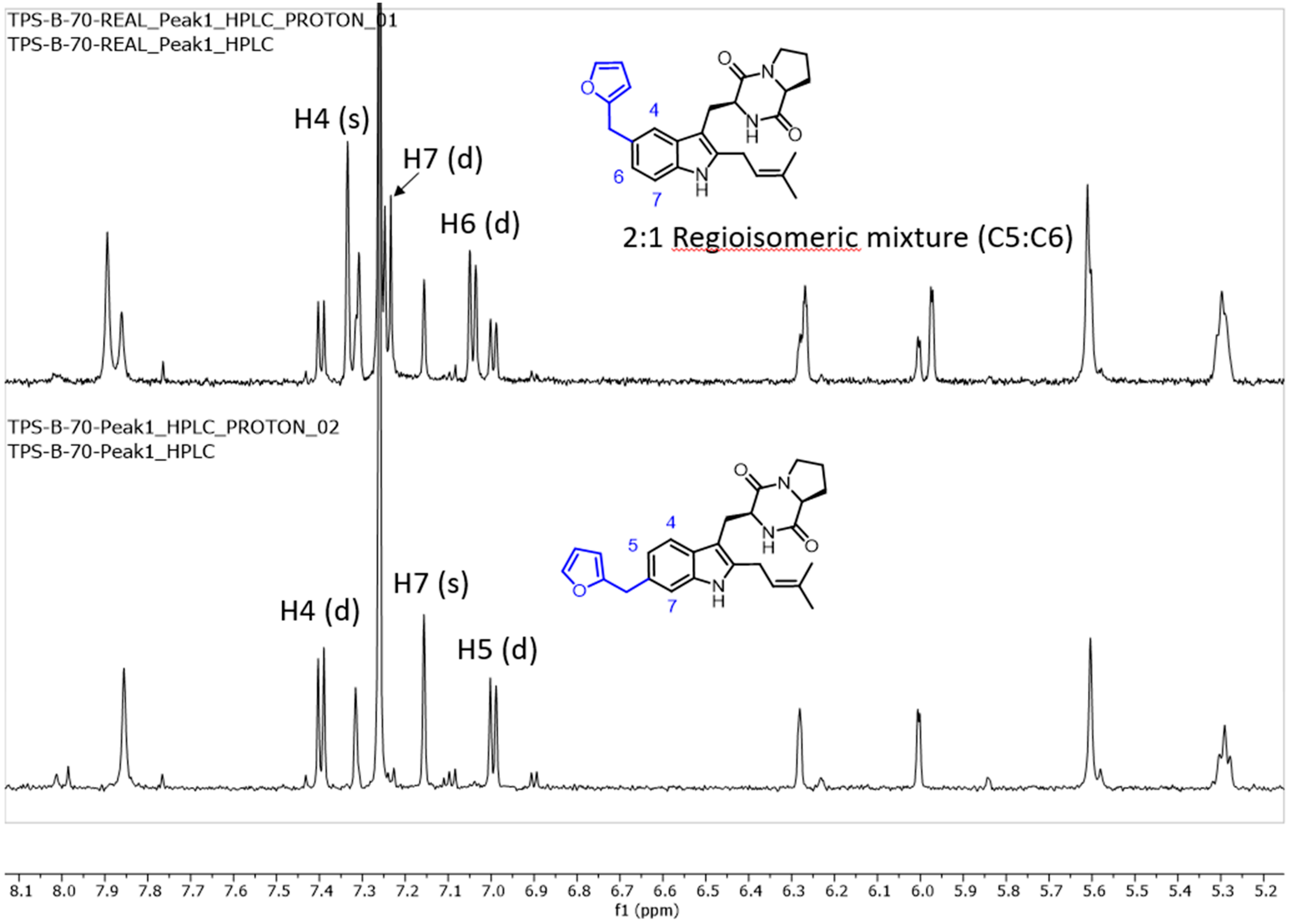

Stacked ^{1}H spectra of **TPS-61** fractions after partial purification. The top spectrum displays the H7 doublet and H4 singlet indicative of C-5 alkylation as a minor reaction product. The bottom spectrum is the purified C6 alkylated major product.

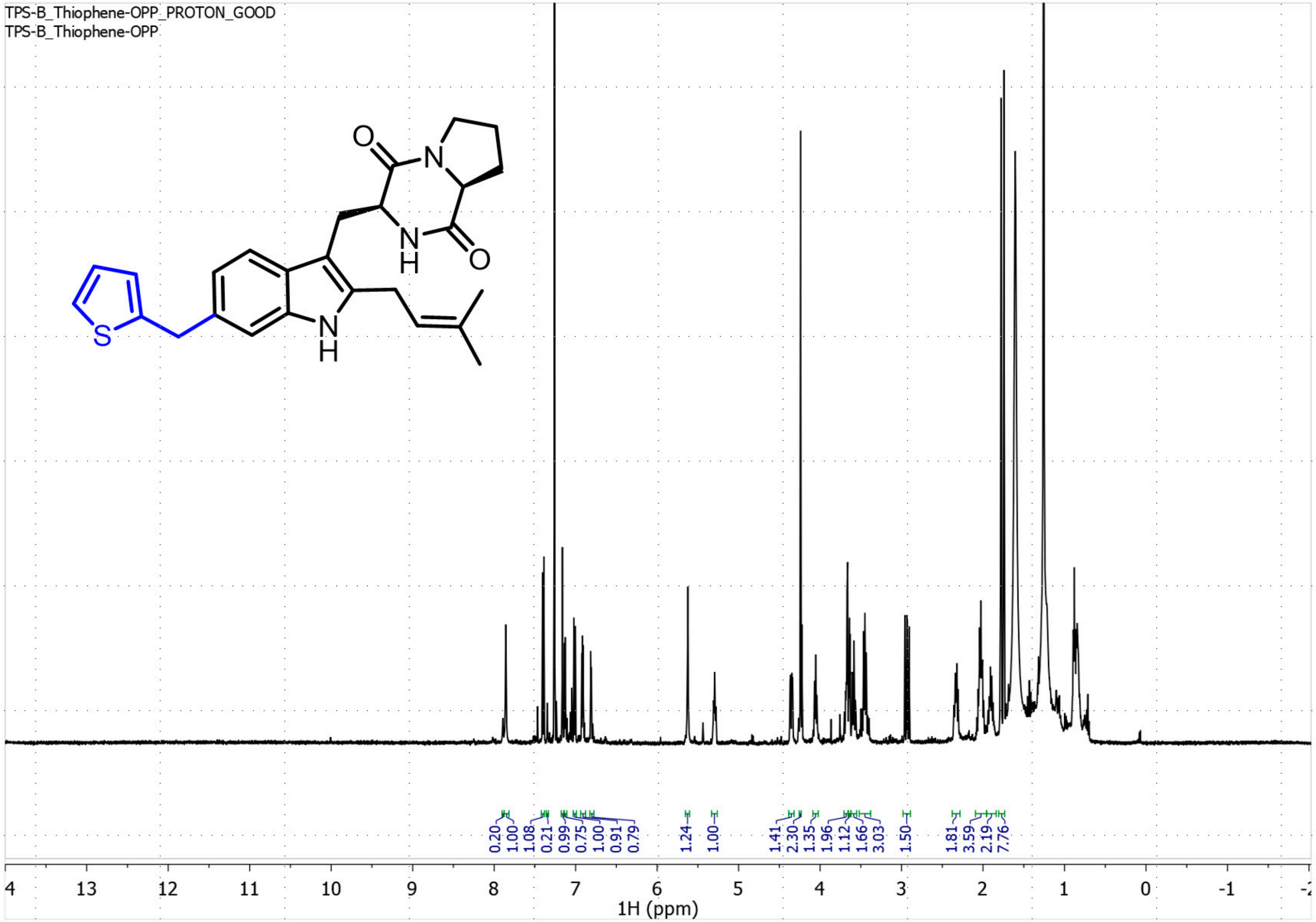

^{1}H NMR spectrum (500 MHz) of **TPS-62** in $CDCl_3$. The minor C5 alkylated product is 20% of the products based on integrations.

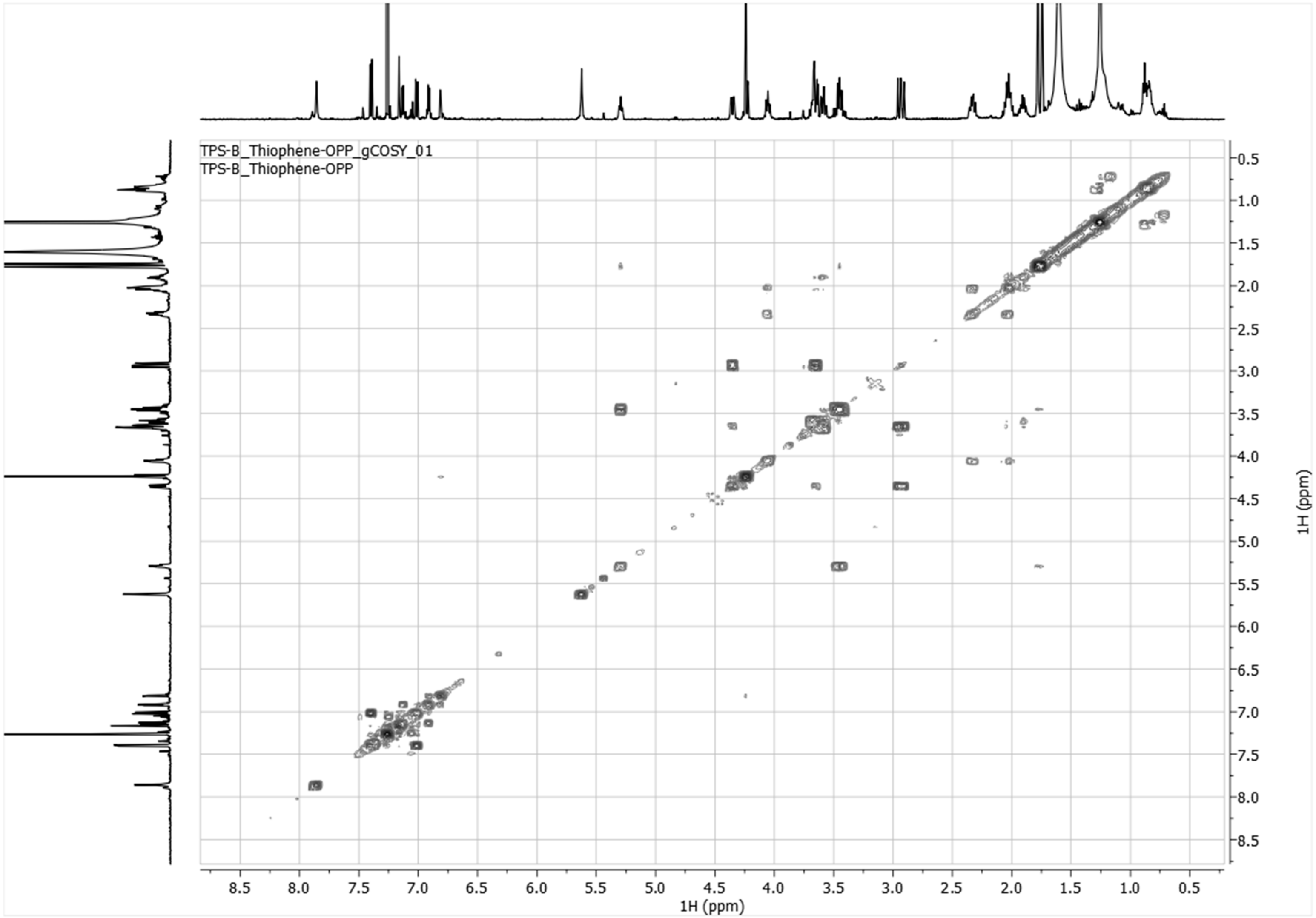

COSY NMR spectrum (500 MHz) of **TPS-62** in $CDCl_3$

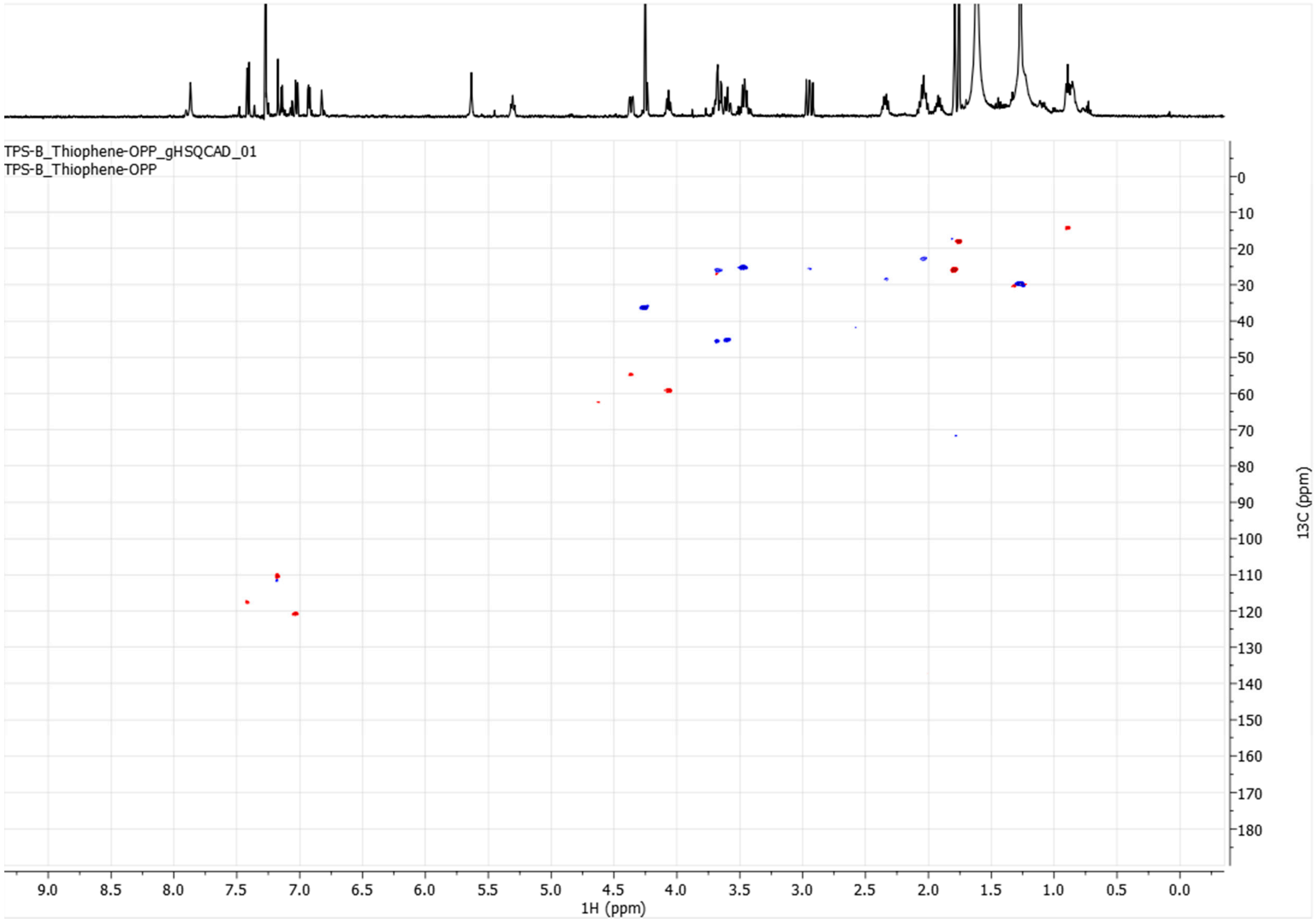

HSQC NMR spectrum (500 MHz) of **TPS-62** in $CDCl_3$

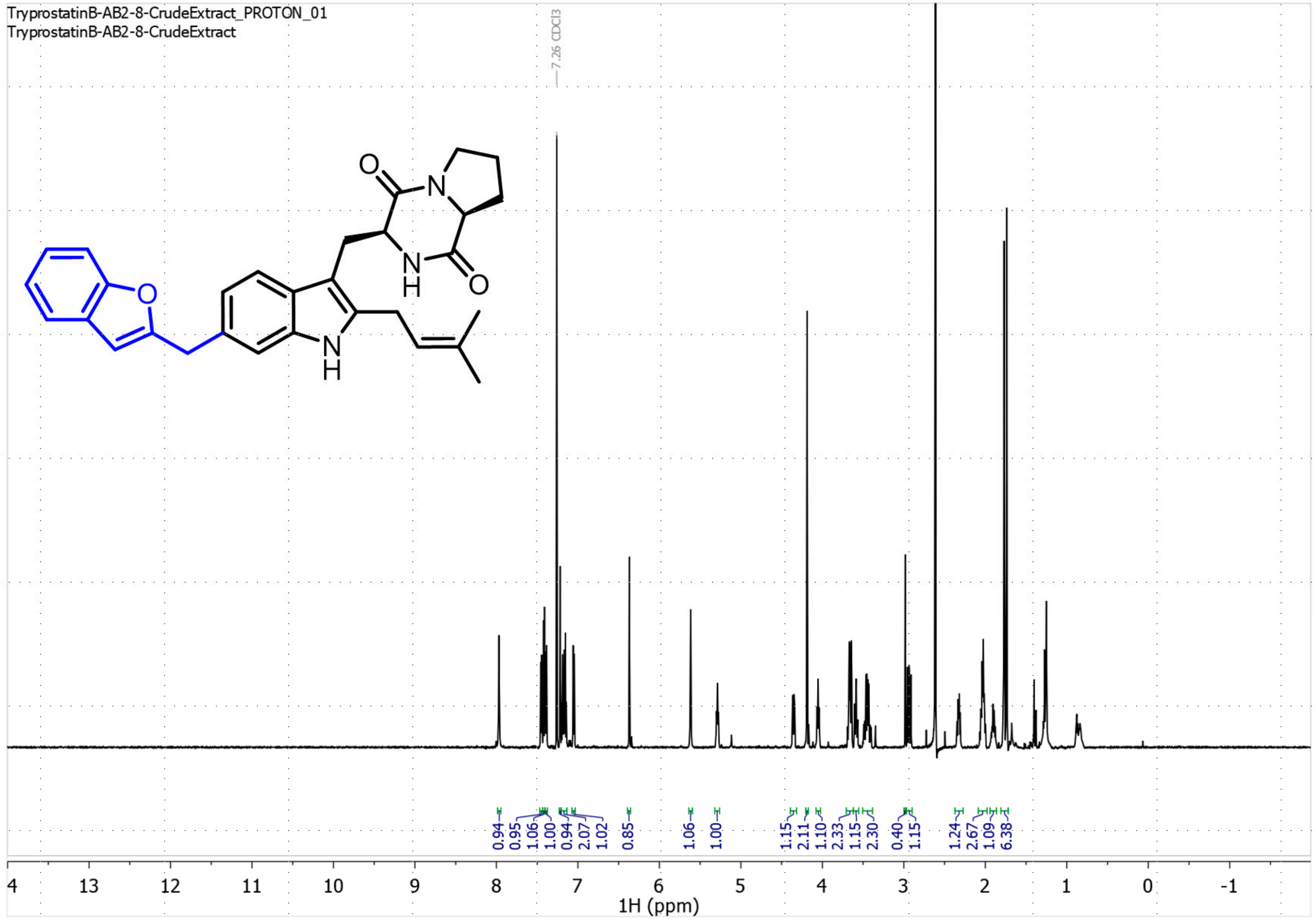

^{1}H NMR spectrum (600 MHz) of **TPS-65** in $CDCl_3$

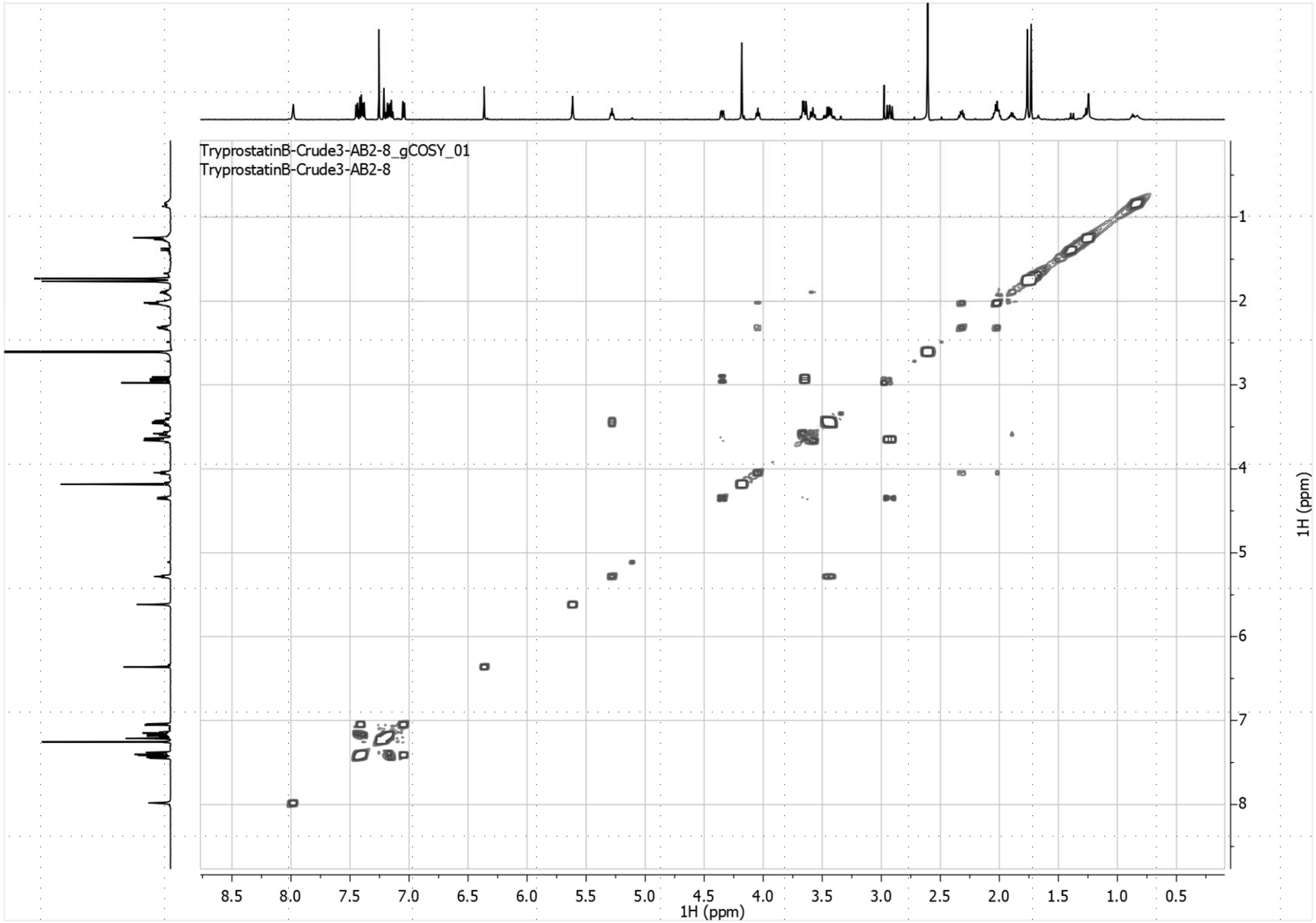

COSY NMR spectrum (600 MHz) of **TPS-65** in $CDCl_3$

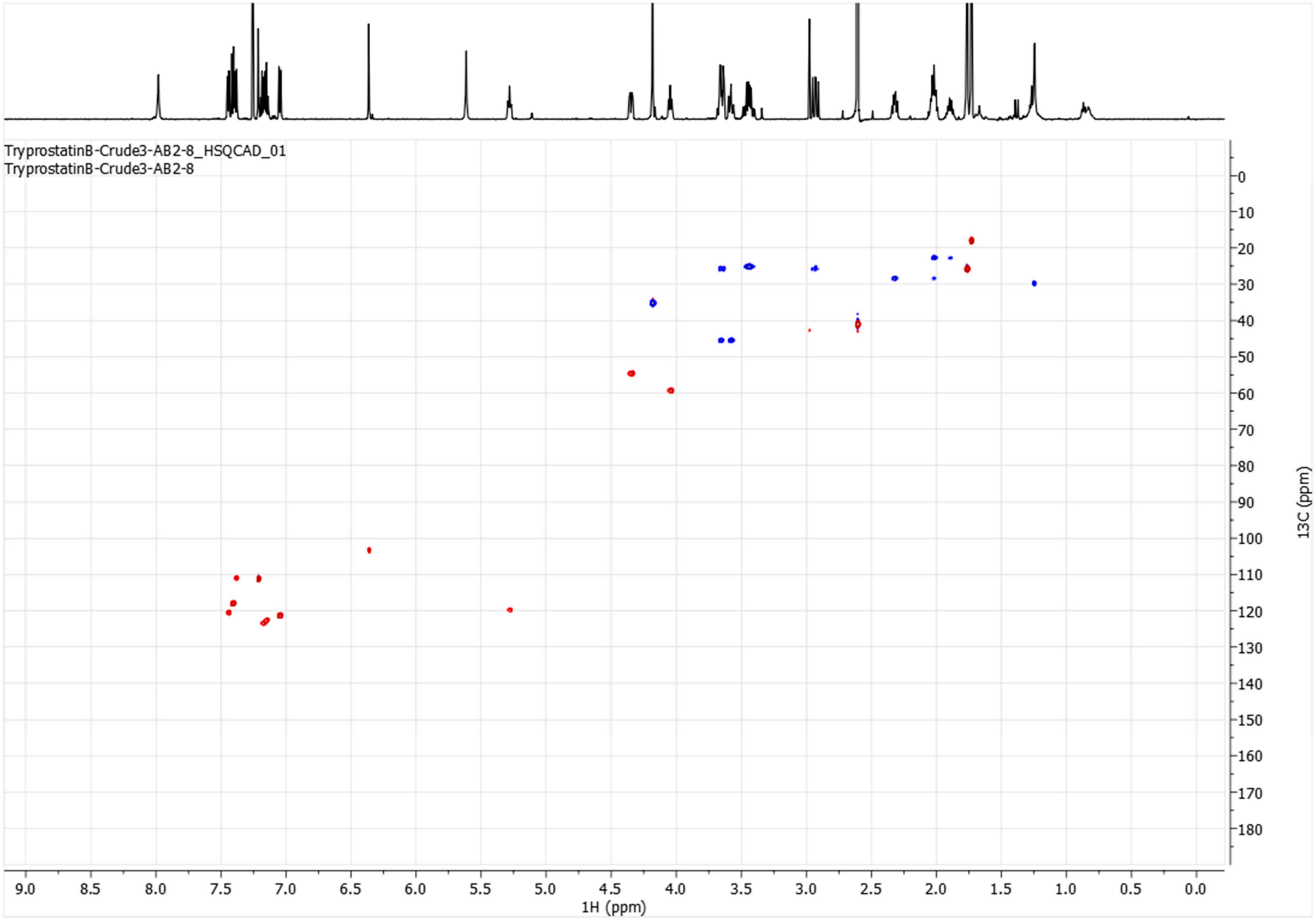

HSQC NMR spectrum (600 MHz) of **TPS-65** in $CDCl_3$

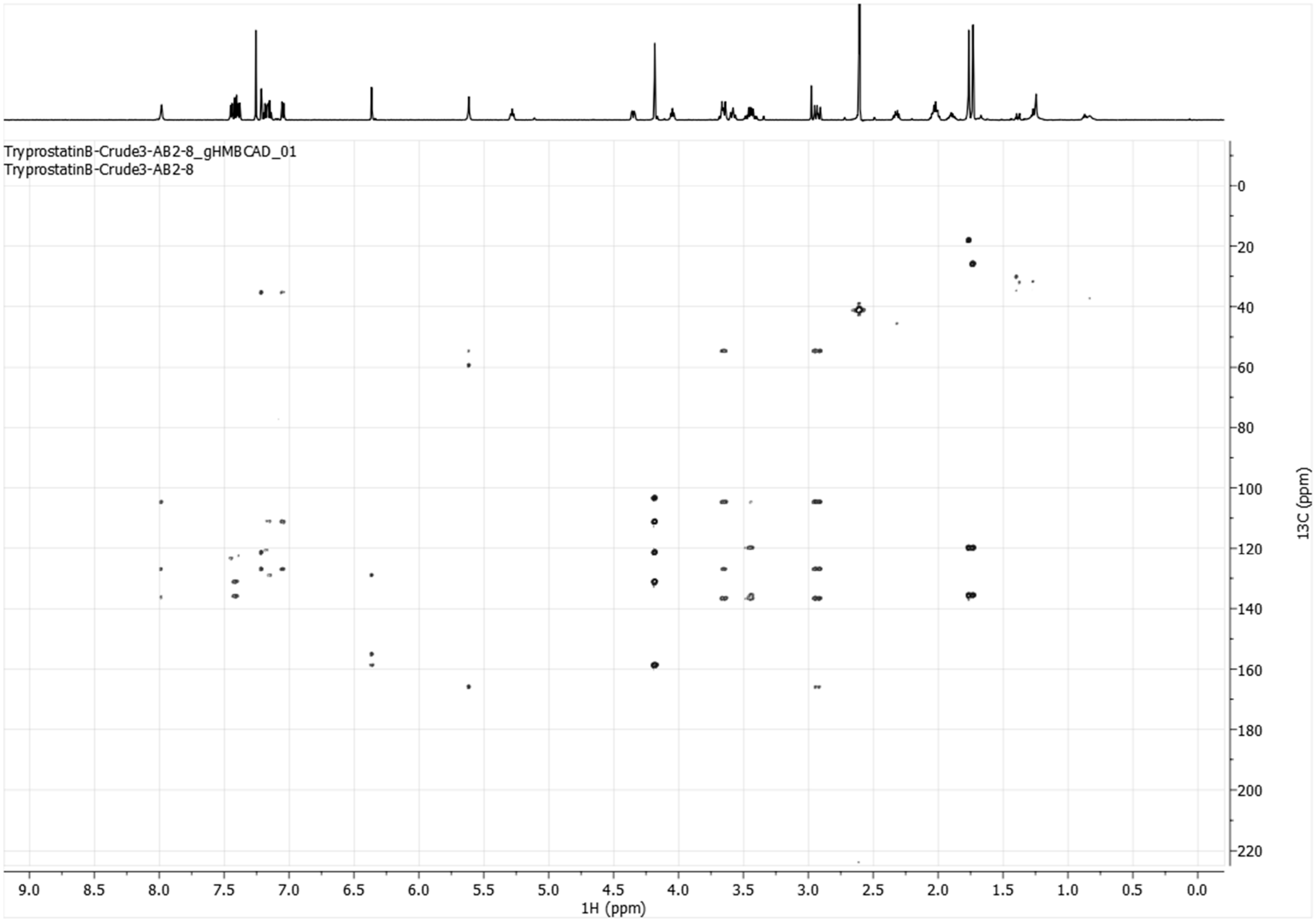

HMBC NMR spectrum (600 MHz) of **TPS-65** in $CDCl_3$

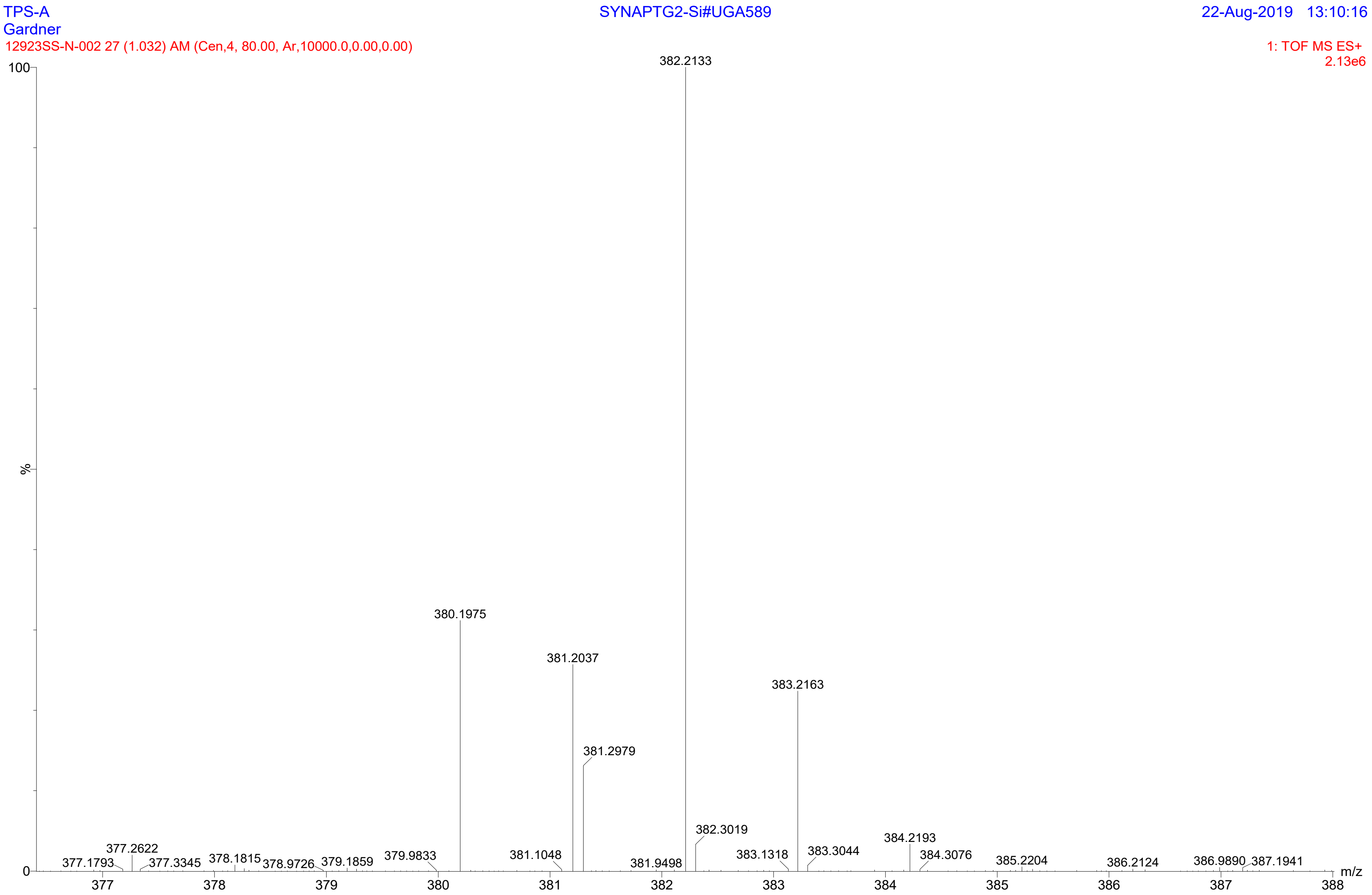
TPS-A
Gardner
SYNAPTG2-Si#UGA589
22-Aug-2019 13:10:16
12923SS-N-002 27 (1.032) AM (Cen,4, 80.00, Ar,10000.0,0.00,0.00)
1: TOF MS ES+
2.13e6
100
%
0
382.2133
380.1975
381.2037
383.2163
381.2979
382.3019
384.2193
377.2622
377.1793
377.3345
378.1815
378.9726
379.1859
379.9833
381.1048
381.9498
383.1318
383.3044
384.3076
385.2204
386.2124
386.9890
387.1941
377
378
379
380
381
382
383
384
385
386
387
388
m/z

HRMS of **TPS-A**

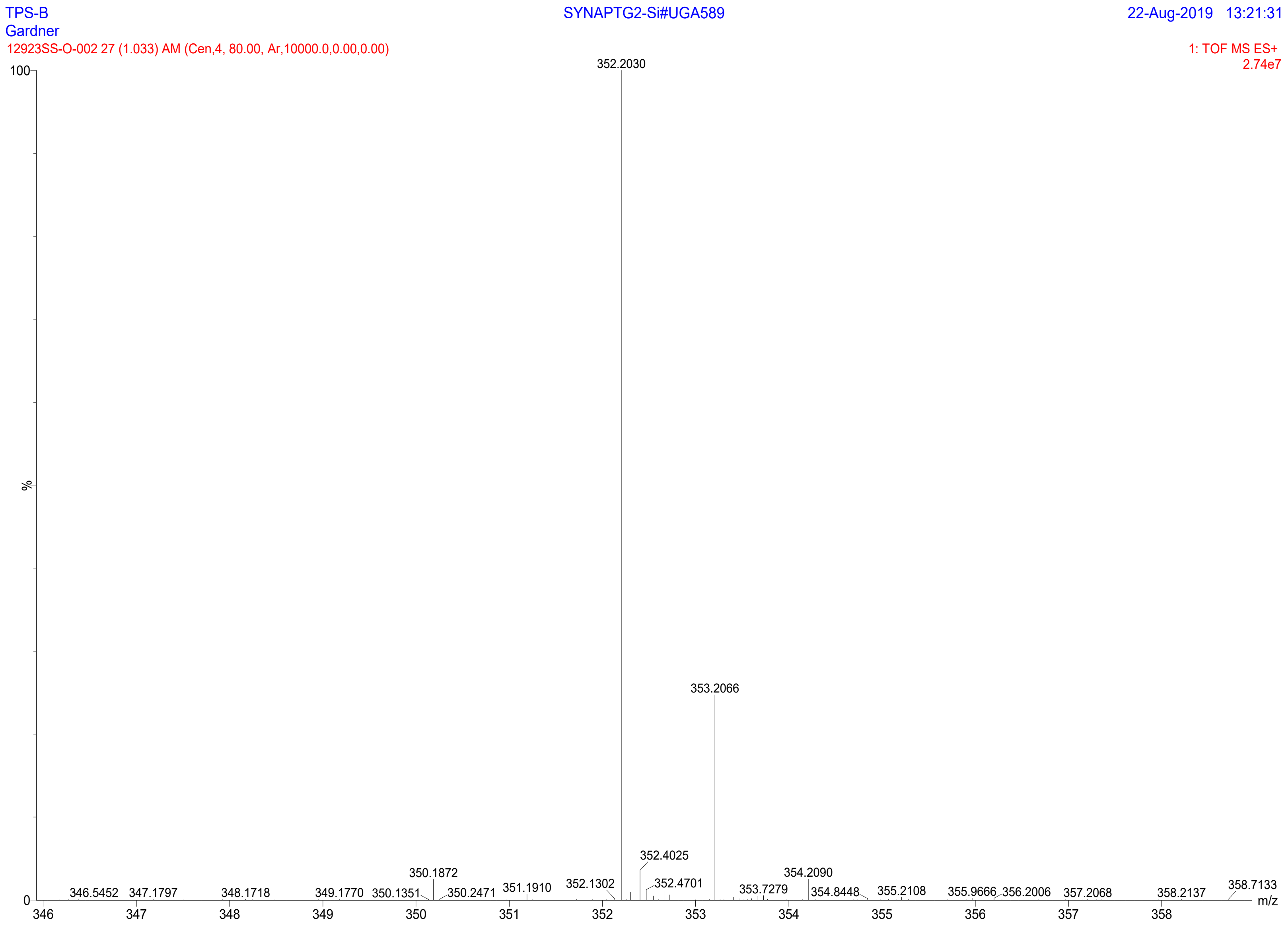
TPS-B
Gardner
12923SS-O-002 27 (1.033) AM (Cen,4, 80.00, Ar,10000.0,0.00,0.00)
SYNAPTG2-Si#UGA589
22-Aug-2019 13:21:31
1: TOF MS ES+
2.74e7
100
%
0
352.2030
353.2066
352.4025
352.4701
350.1872
354.2090
346.5452
347.1797
348.1718
349.1770
350.1351
350.2471
351.1910
352.1302
353.7279
354.8448
355.2108
355.9666
356.2006
357.2068
358.2137
358.7133
346
347
348
349
350
351
352
353
354
355
356
357
358
m/z

HRMS of **TPS-B**

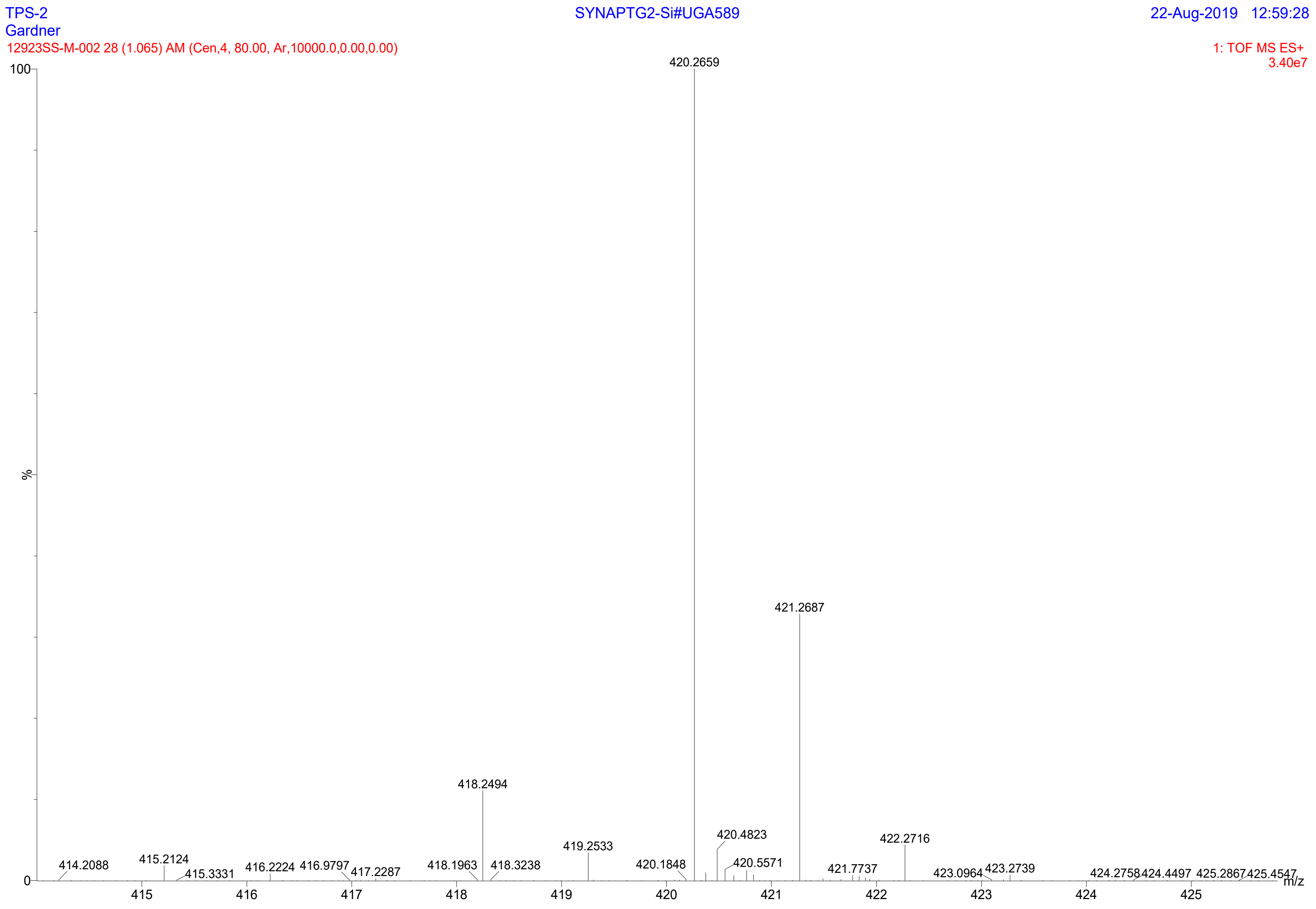
TPS-2
Gardner
SYNAPTG2-Si#UGA589
22-Aug-2019 12:59:28
12923SS-M-002 28 (1.065) AM (Cen,4, 80.00, Ar,10000.0,0.00,0.00)
1: TOF MS ES+
3.40e7
420.2659
421.2687
418.2494
420.4823
422.2716
419.2533
415.2124
414.2088
418.1963
418.3238
420.1848
420.5571
416.2224
416.9797
417.2287
415.3331
421.7737
423.0964
423.2739
424.2758
424.4497
425.2867
425.4547
%
m/z

HRMS of **TPS-2**

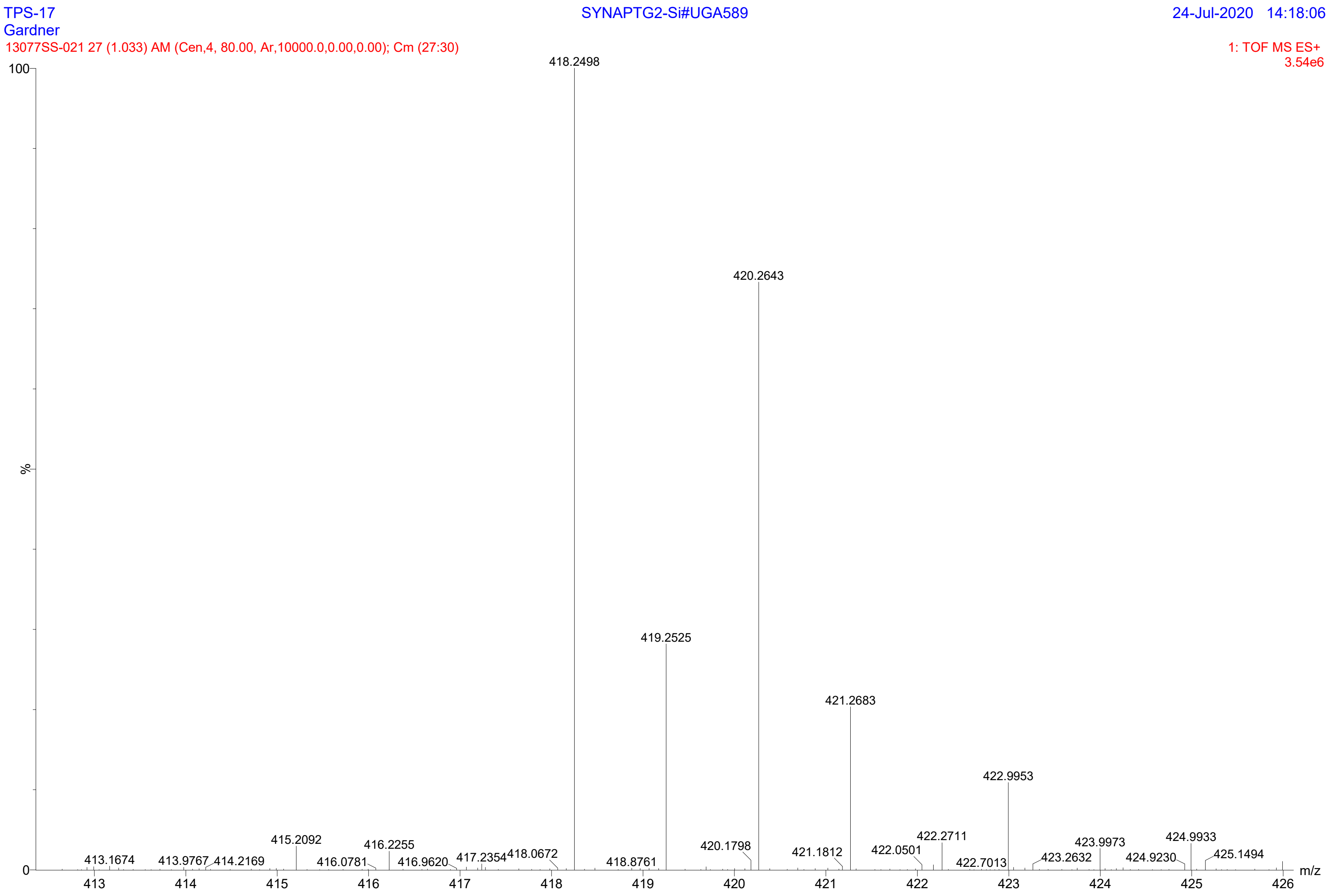
TPS-17
Gardner
SYNAPTG2-Si#UGA589
24-Jul-2020 14:18:06
13077SS-021 27 (1.033) AM (Cen,4, 80.00, Ar,10000.0,0.00,0.00); Cm (27:30)
1: TOF MS ES+
3.54e6
100
%
0
418.2498
420.2643
419.2525
421.2683
422.9953
413.1674
413.9767
414.2169
415.2092
416.0781
416.2255
416.9620
417.2354
418.0672
418.8761
420.1798
421.1812
422.0501
422.2711
422.7013
423.2632
423.9973
424.9230
424.9933
425.1494
m/z
413
414
415
416
417
418
419
420
421
422
423
424
425
426

HRMS of **TPS-6**

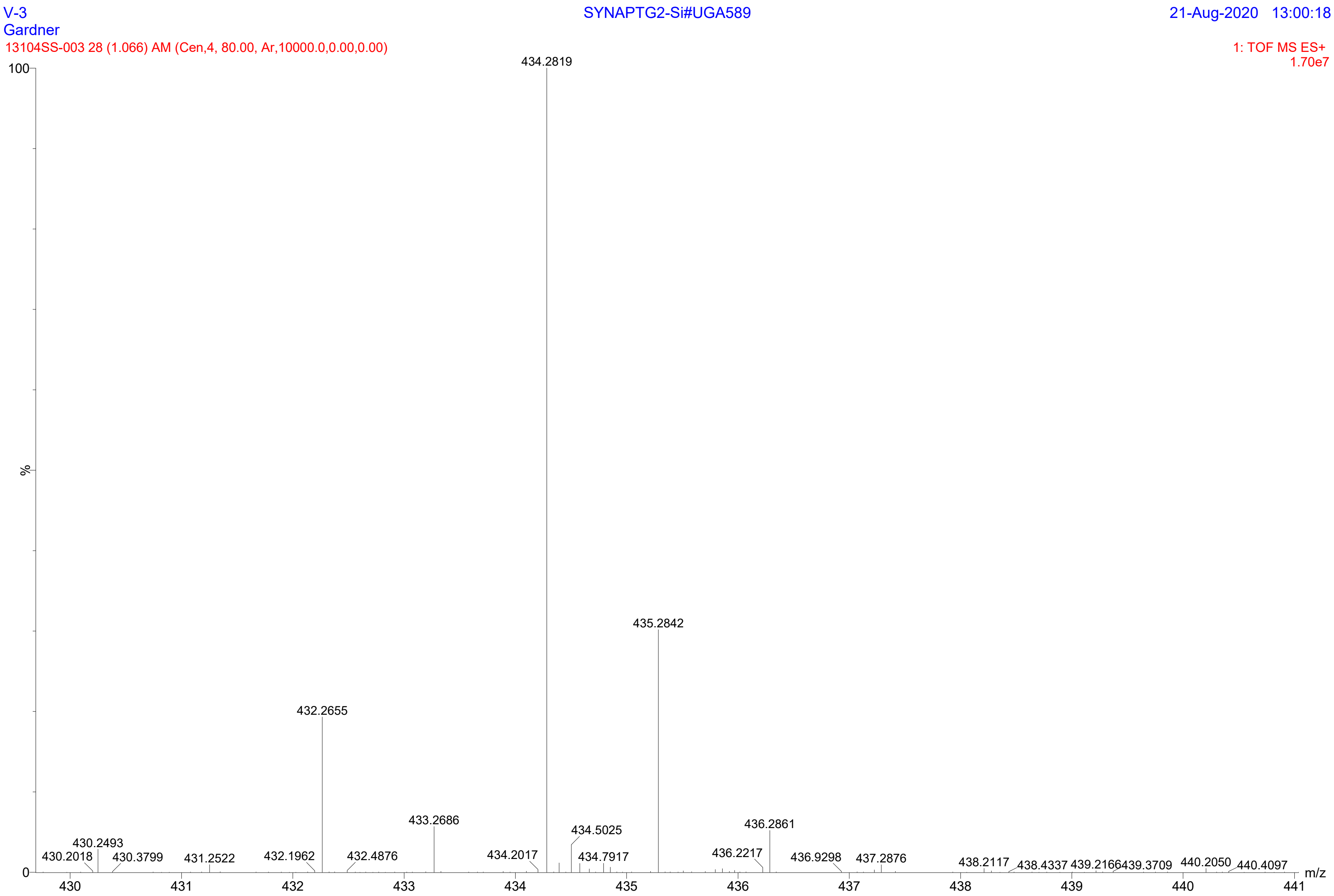

V-3
Gardner
SYNAPTG2-Si#UGA589
21-Aug-2020 13:00:18
13104SS-003 28 (1.066) AM (Cen,4, 80.00, Ar,10000.0,0.00,0.00)
1: TOF MS ES+
1.70e7
434.2819
435.2842
432.2655
433.2686
434.5025
436.2861
430.2493
430.2018
430.3799
431.2522
432.1962
432.4876
434.2017
434.7917
436.2217
436.9298
437.2876
438.2117
438.4337
439.2166
439.3709
440.2050
440.4097
100
%
0
m/z
430
431
432
433
434
435
436
437
438
439
440
441

HRMS of **TPS-7**

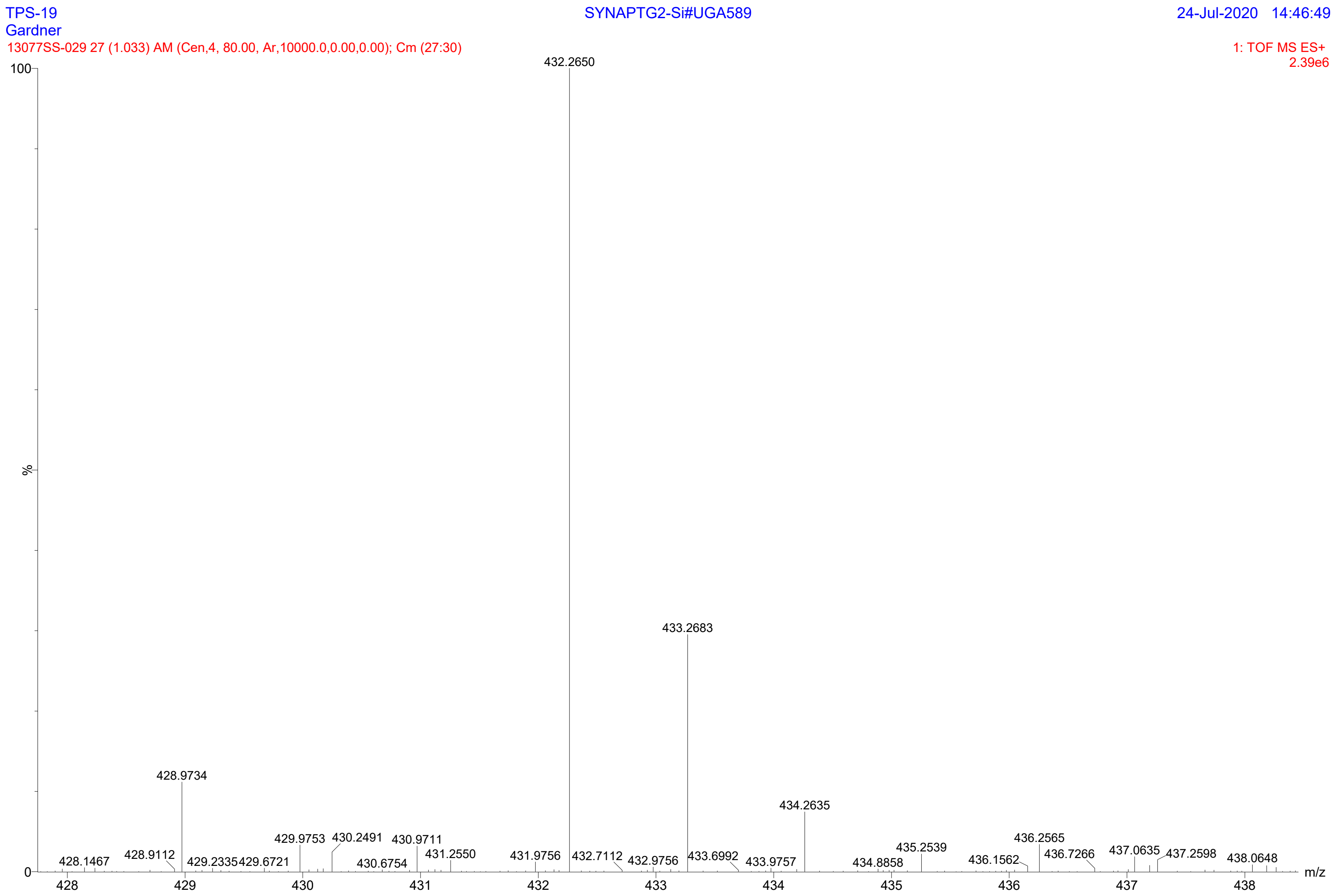
TPS-19
Gardner
SYNAPTG2-Si#UGA589
24-Jul-2020 14:46:49
13077SS-029 27 (1.033) AM (Cen,4, 80.00, Ar,10000.0,0.00,0.00); Cm (27:30)
1: TOF MS ES+
2.39e6
432.2650
433.2683
428.9734
434.2635
%
m/z

HRMS of **TPS-8**

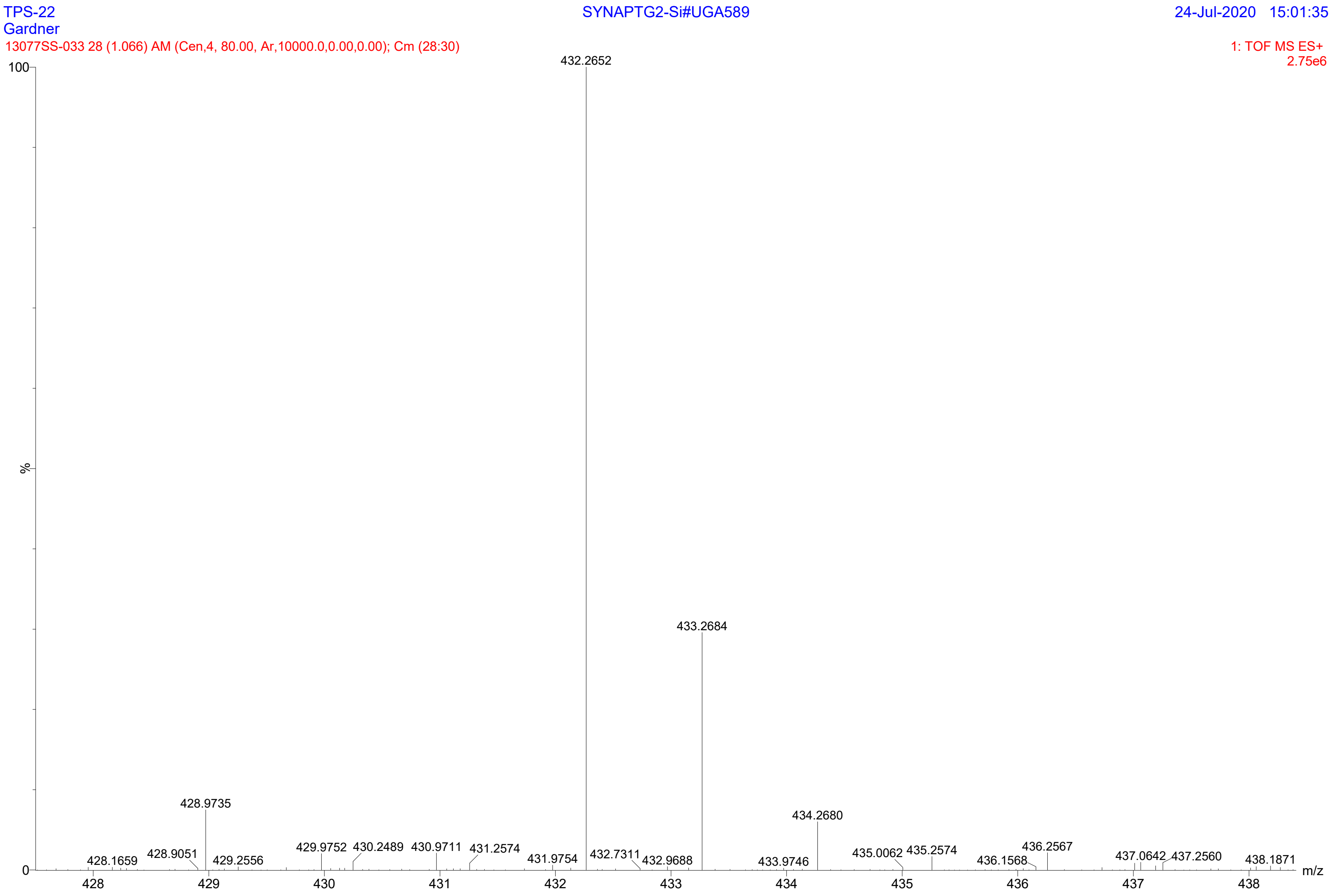
TPS-22
Gardner
SYNAPTG2-Si#UGA589
24-Jul-2020 15:01:35
13077SS-033 28 (1.066) AM (Cen,4, 80.00, Ar,10000.0,0.00,0.00); Cm (28:30)
1: TOF MS ES+
2.75e6
100
%
0
432.2652
433.2684
428.9735
434.2680
428.1659
428.9051
429.2556
429.9752
430.2489
430.9711
431.2574
431.9754
432.7311
432.9688
433.9746
435.0062
435.2574
436.1568
436.2567
437.0642
437.2560
438.1871
428
429
430
431
432
433
434
435
436
437
438
m/z

HRMS of **TPS-9**

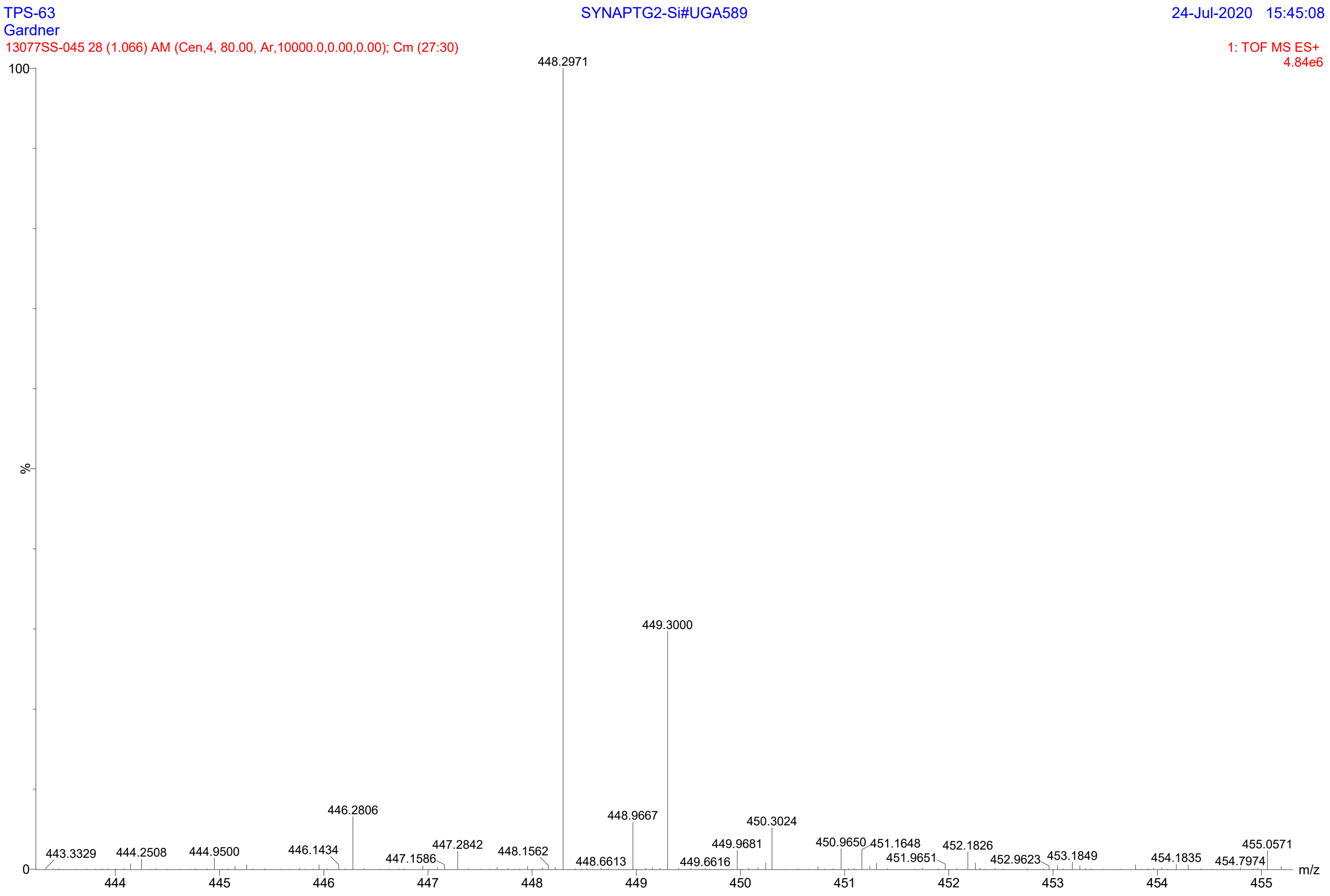

HRMS of **TPS-10**

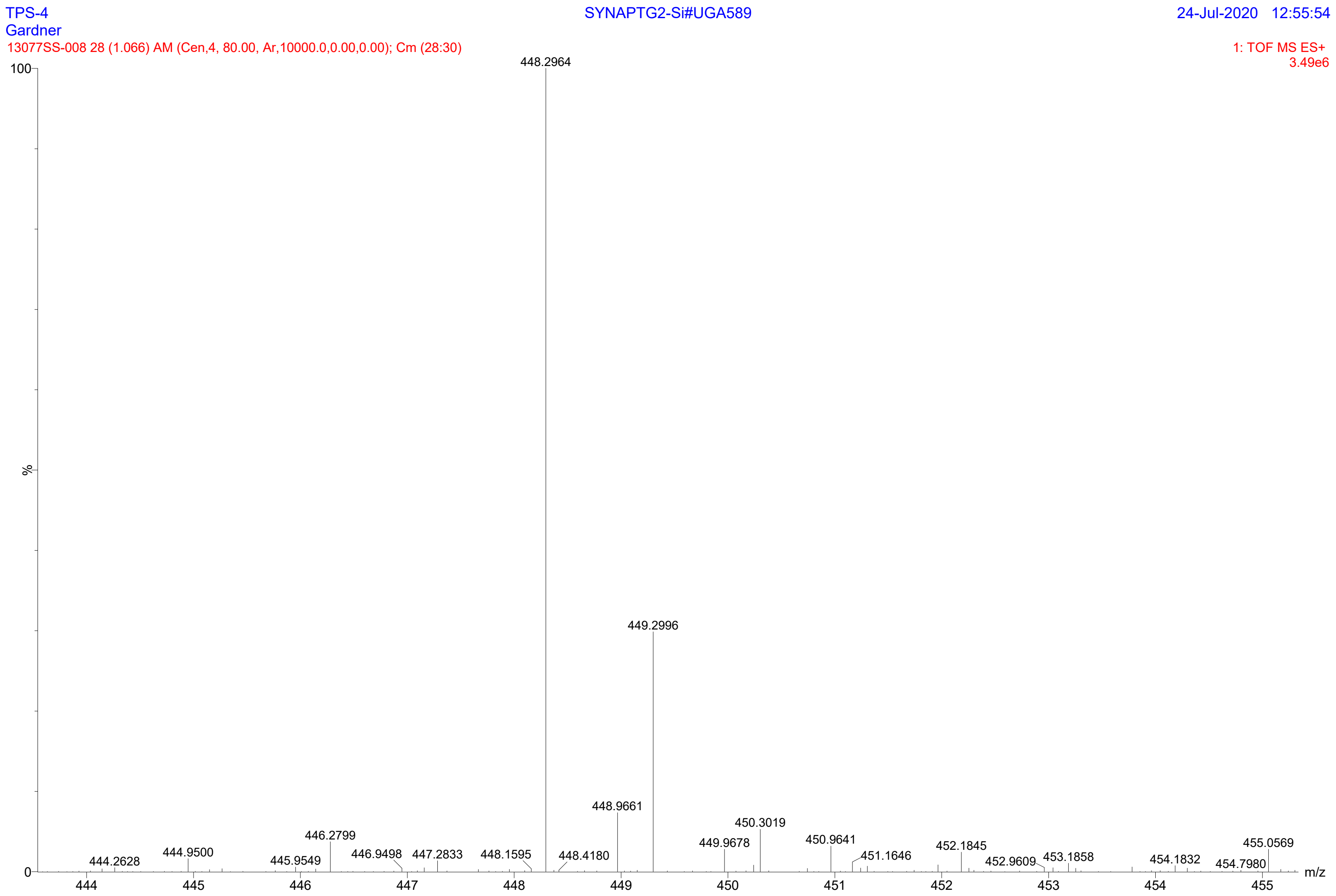

HRMS of **TPS-11**

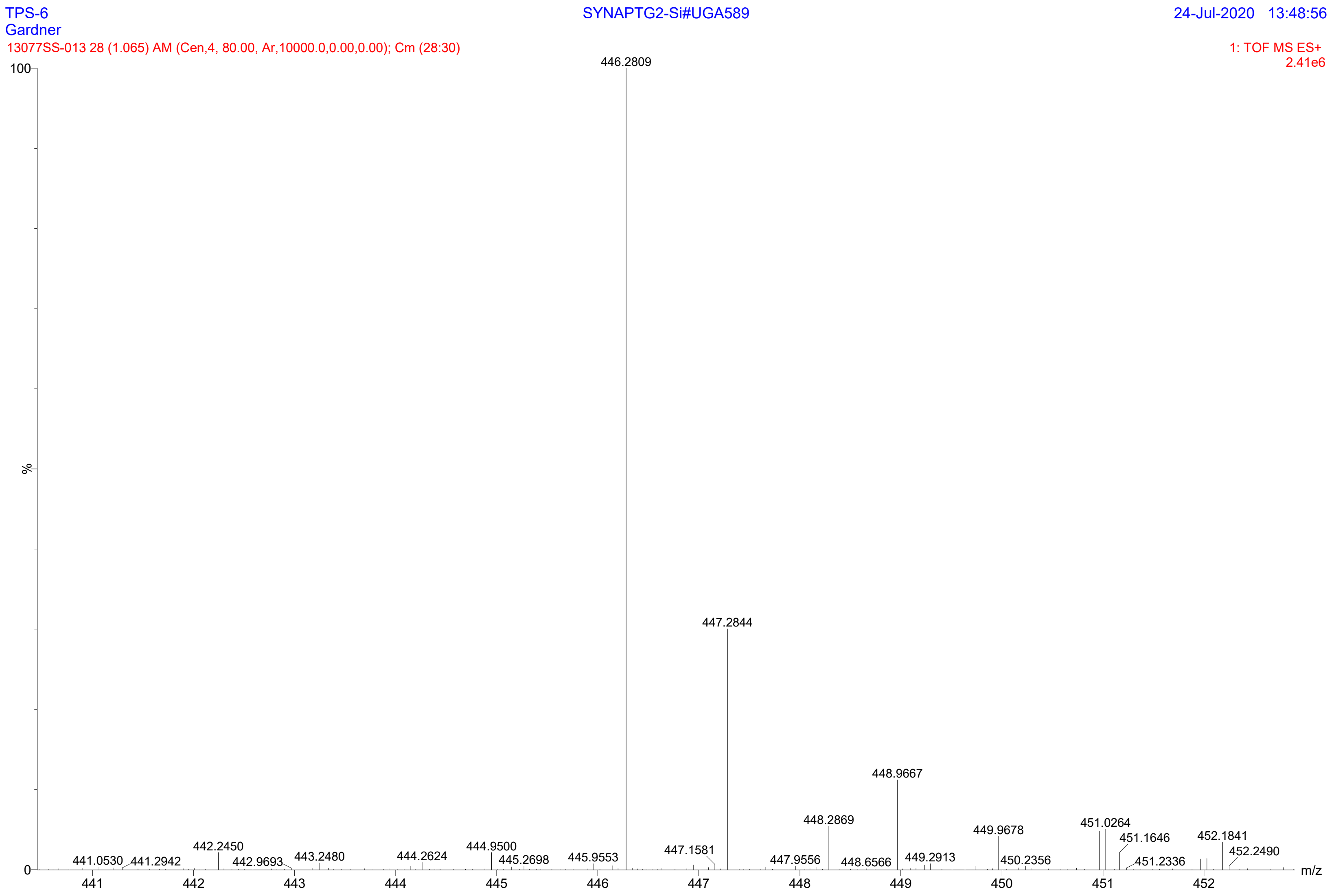

HRMS of **TPS-13**

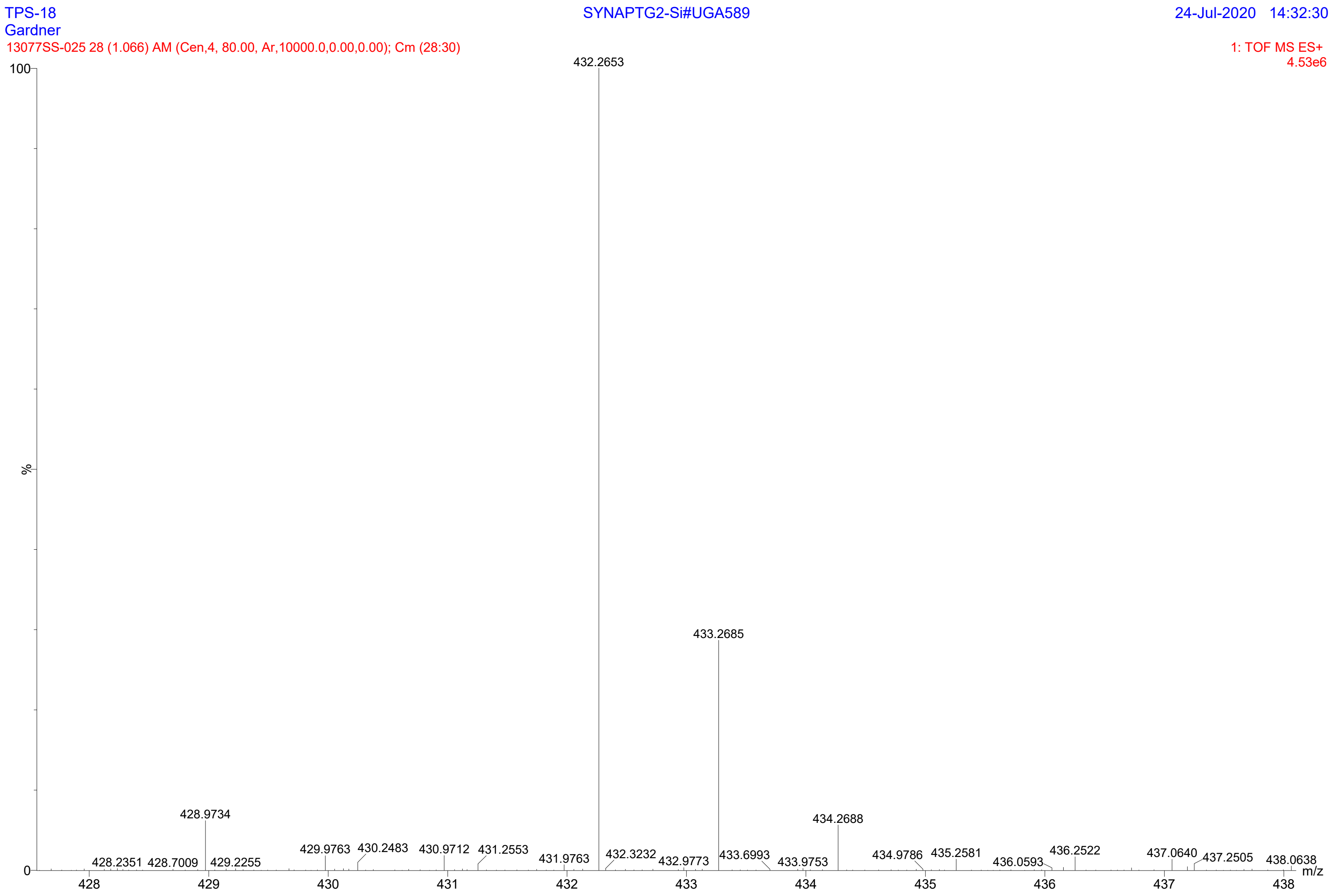

HRMS of **TPS-16**

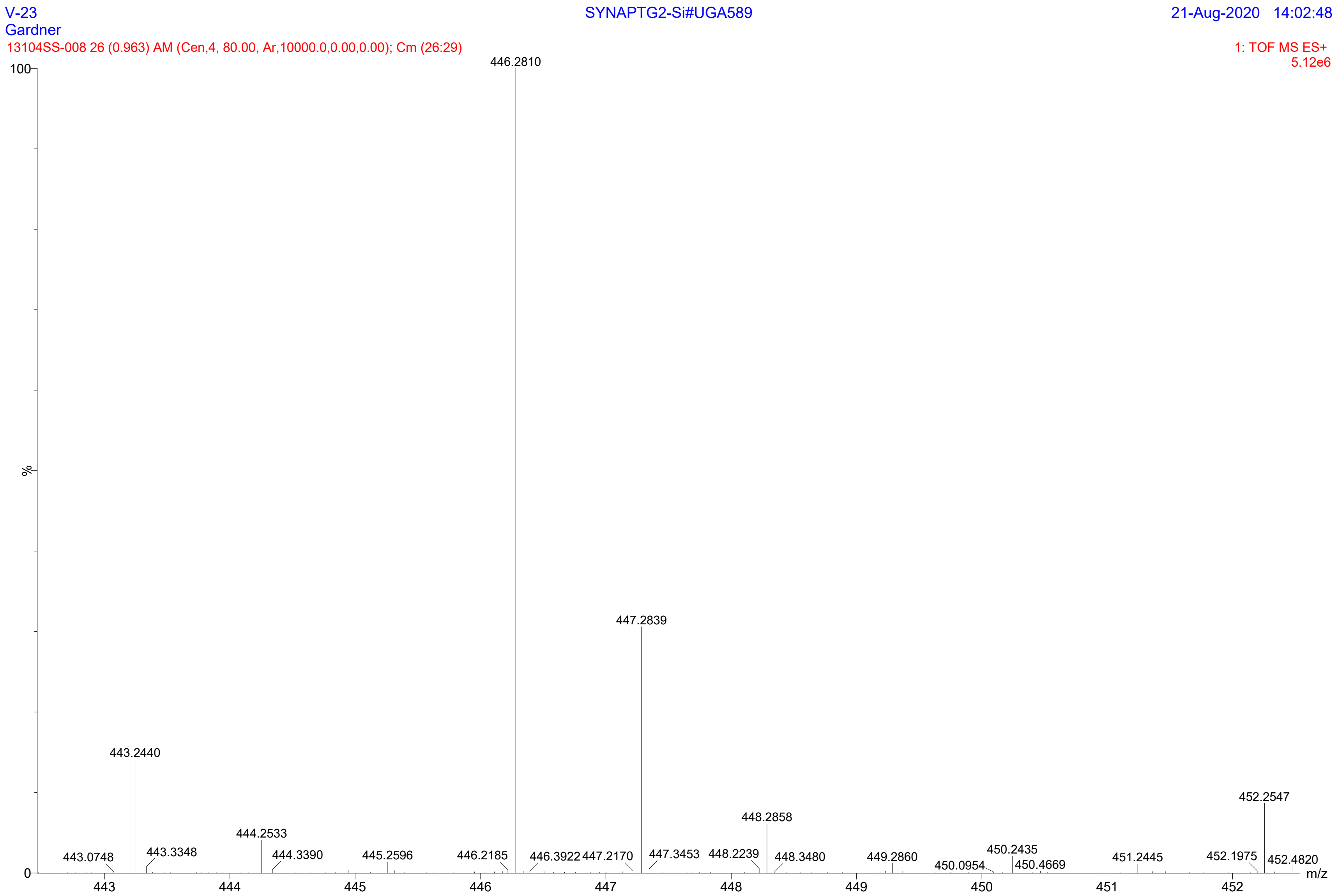

HRMS of **TPS-18**

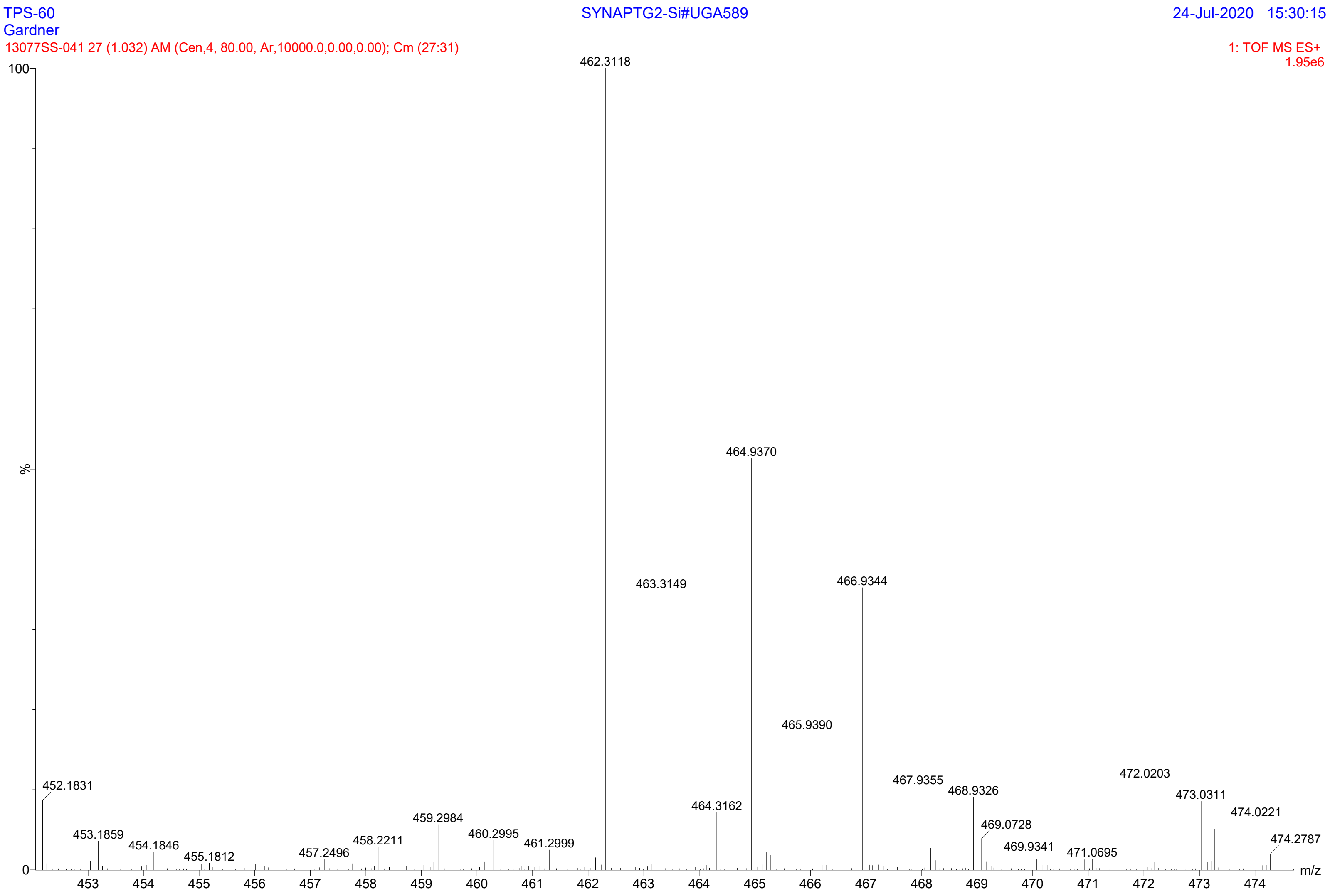

HRMS of **TPS-19**

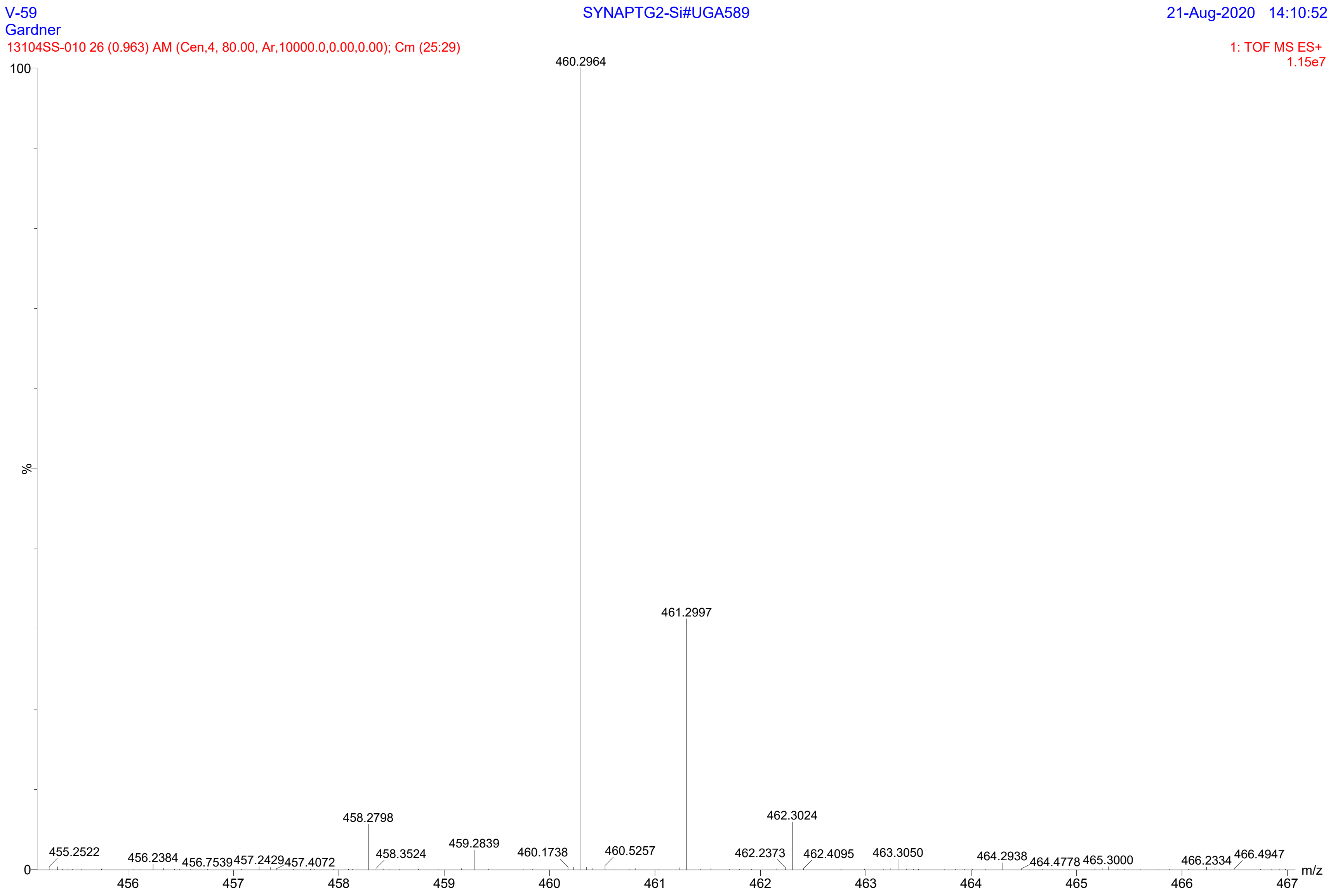

HRMS of **TPS-22**

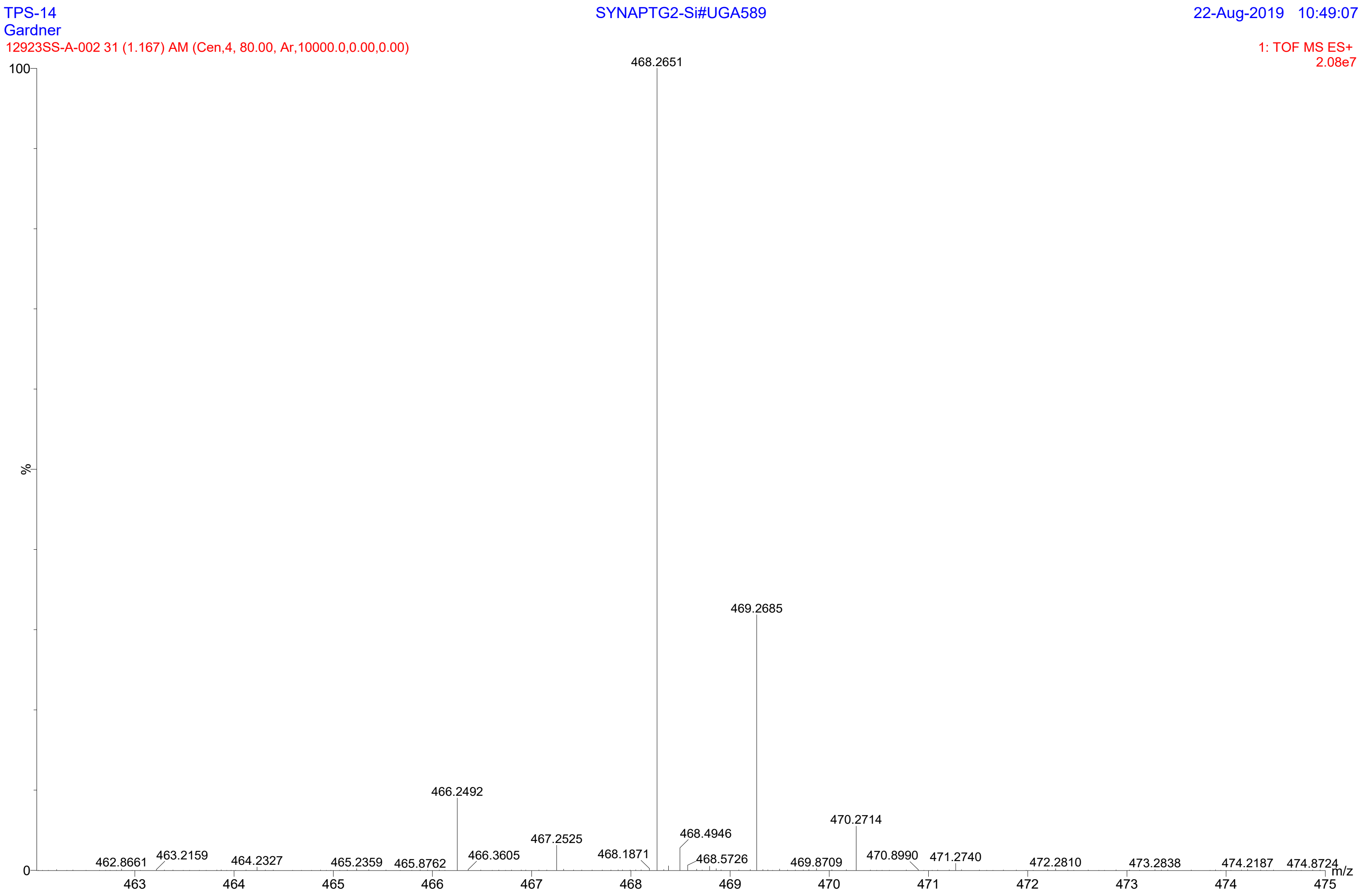

HRMS of **TPS-24**

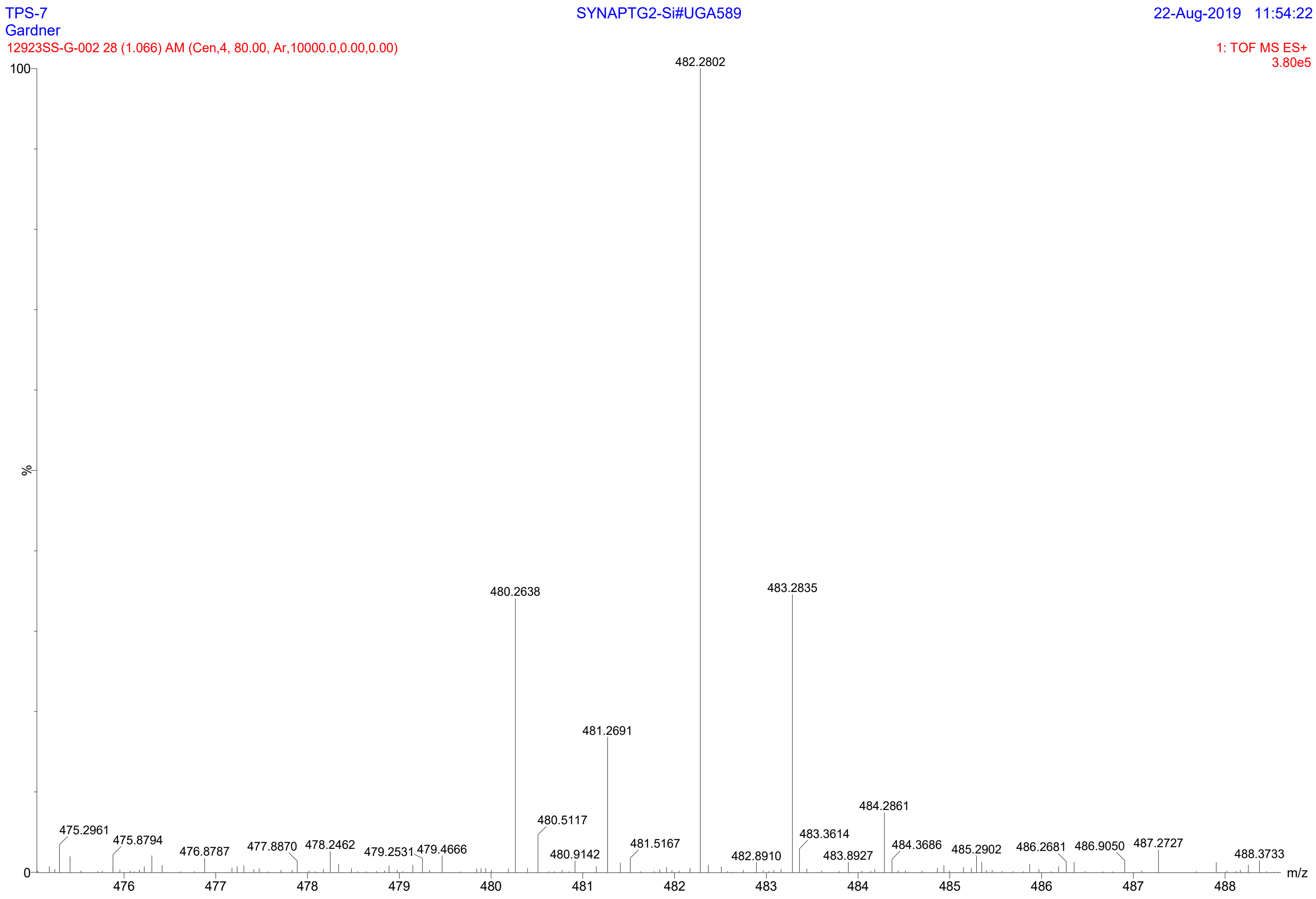

HRMS of **TPS-25**

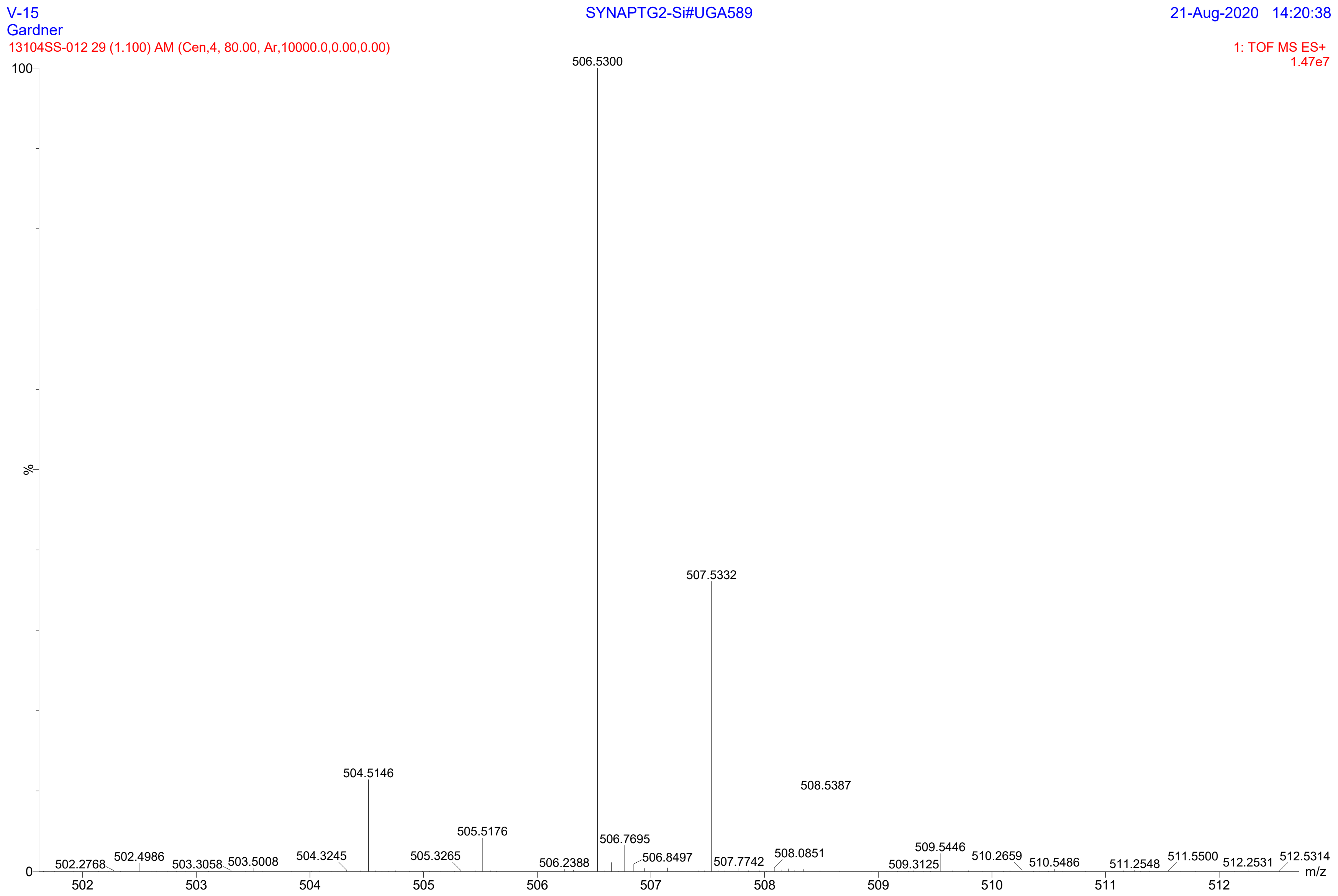

HRMS of **TPS-26**

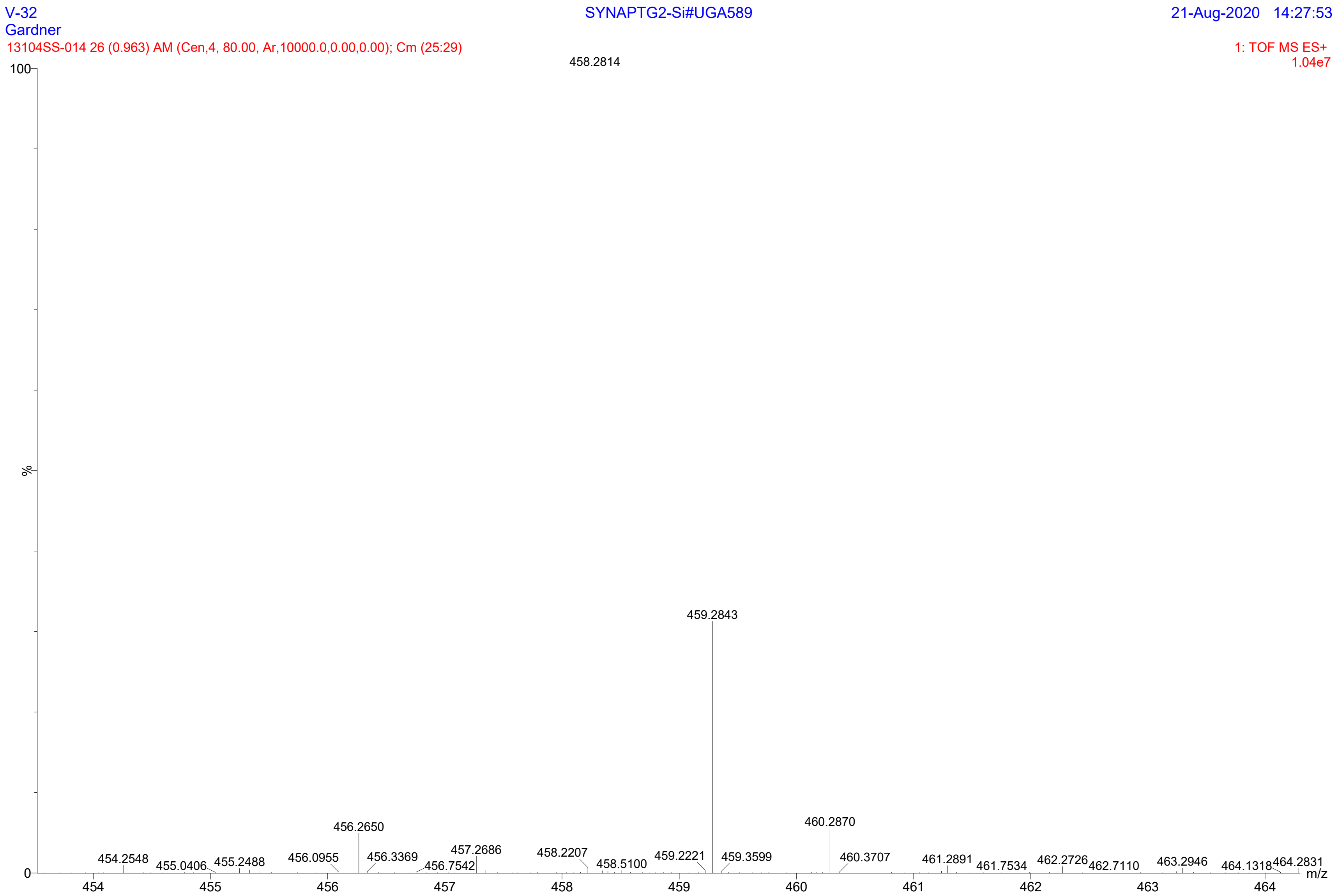

HRMS of **TPS-27**

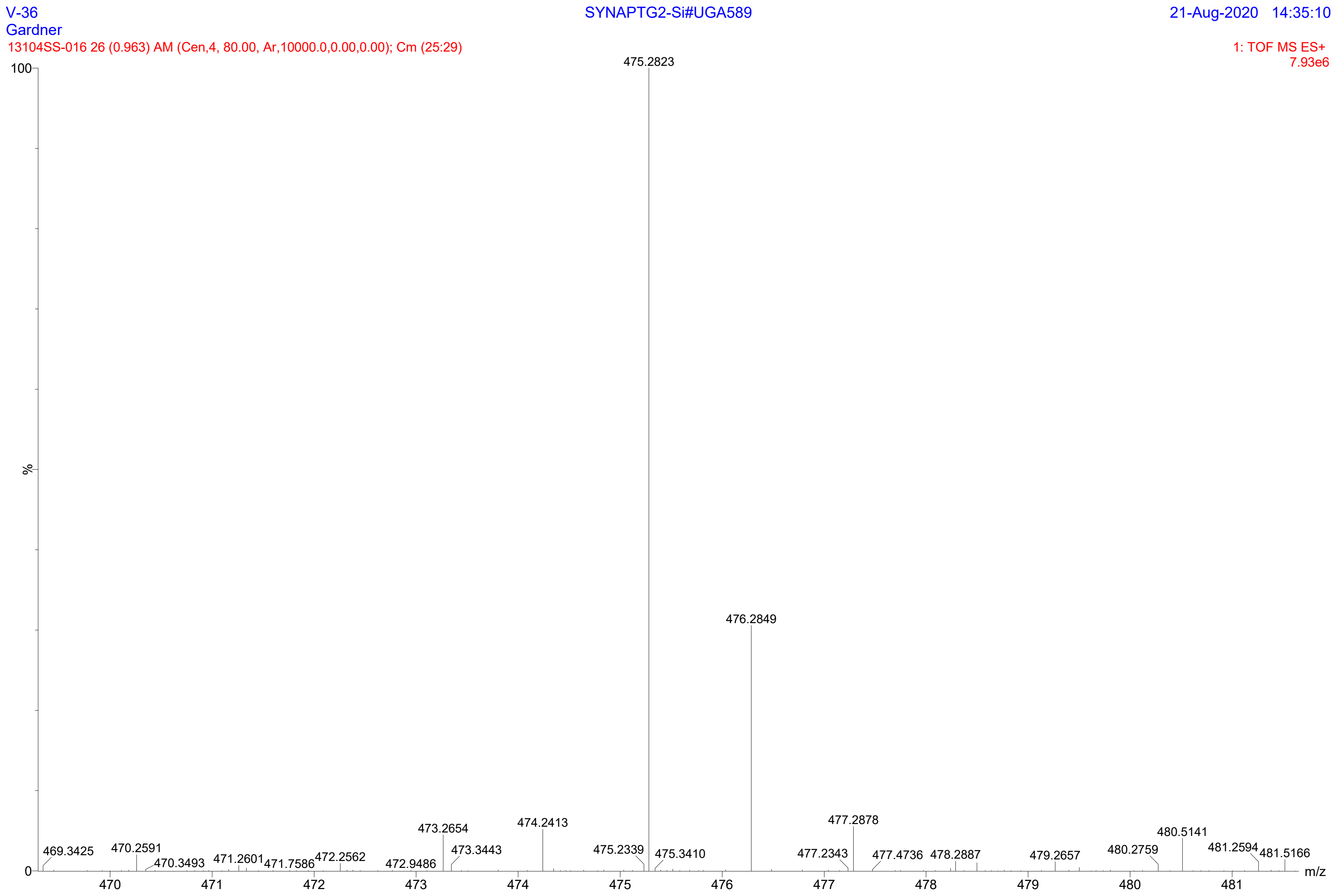

HRMS of **TPS-34**

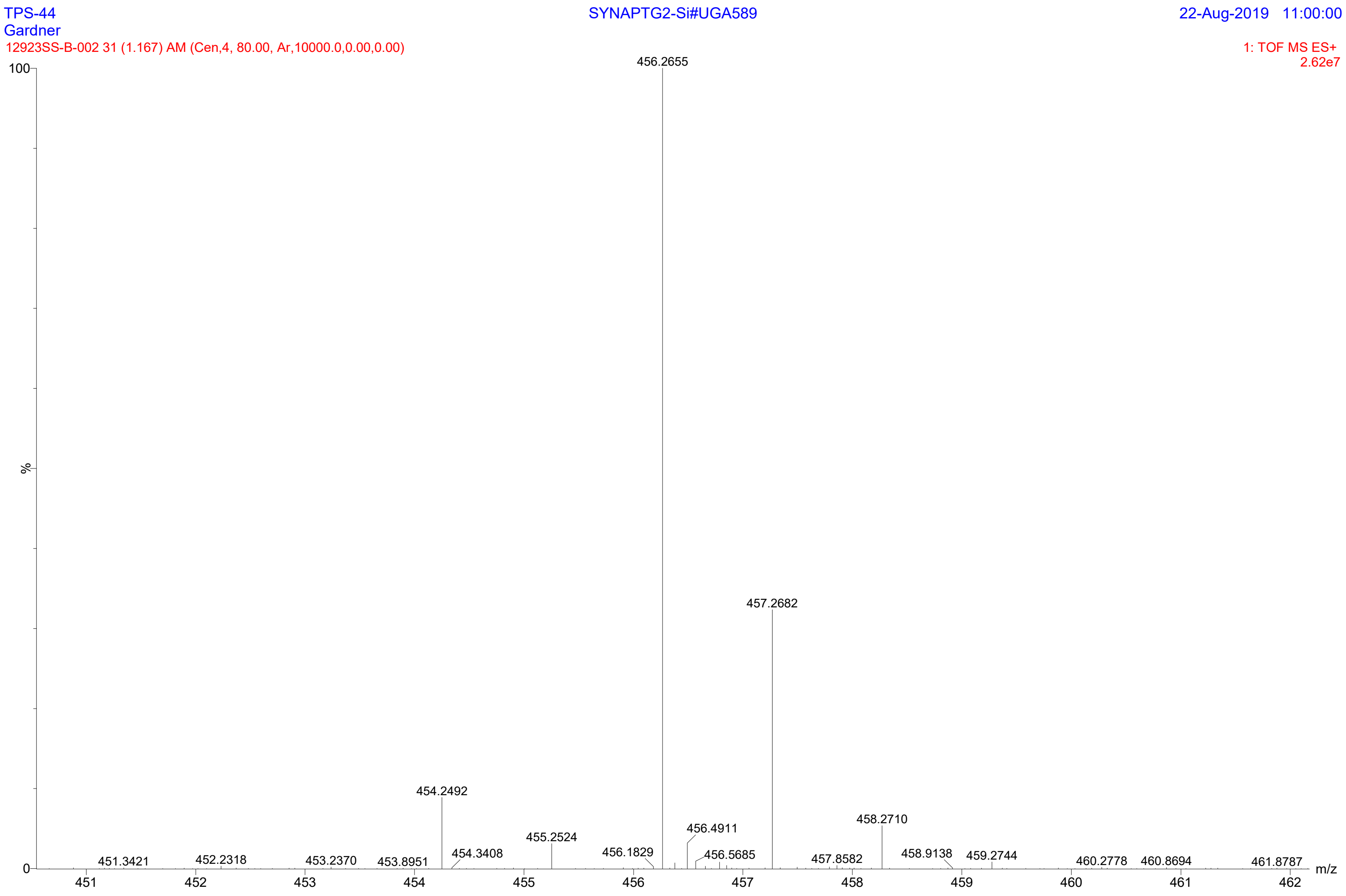

HRMS of **TPS-46**

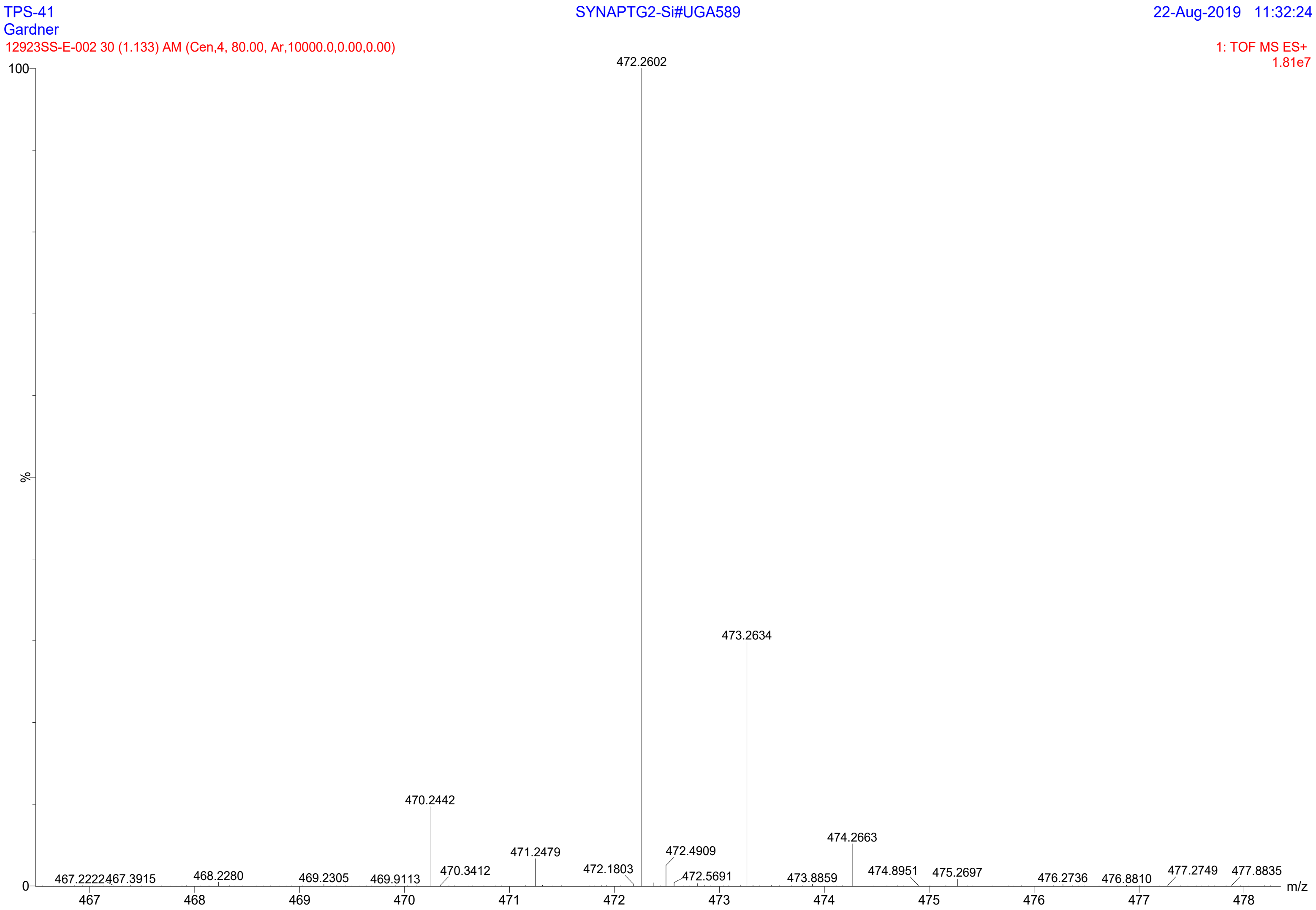

HRMS of **TPS-52**

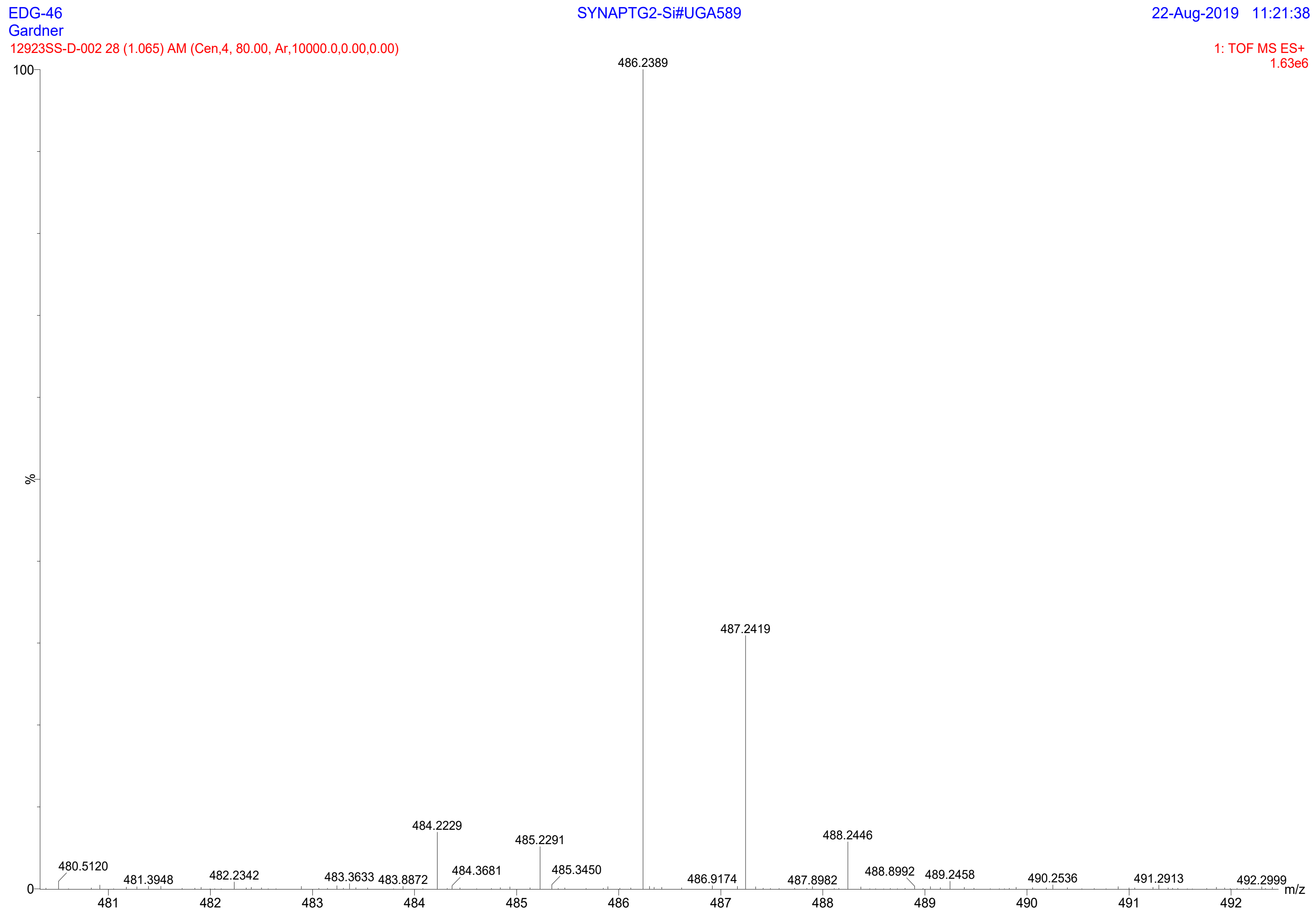

HRMS of **TPS-58**

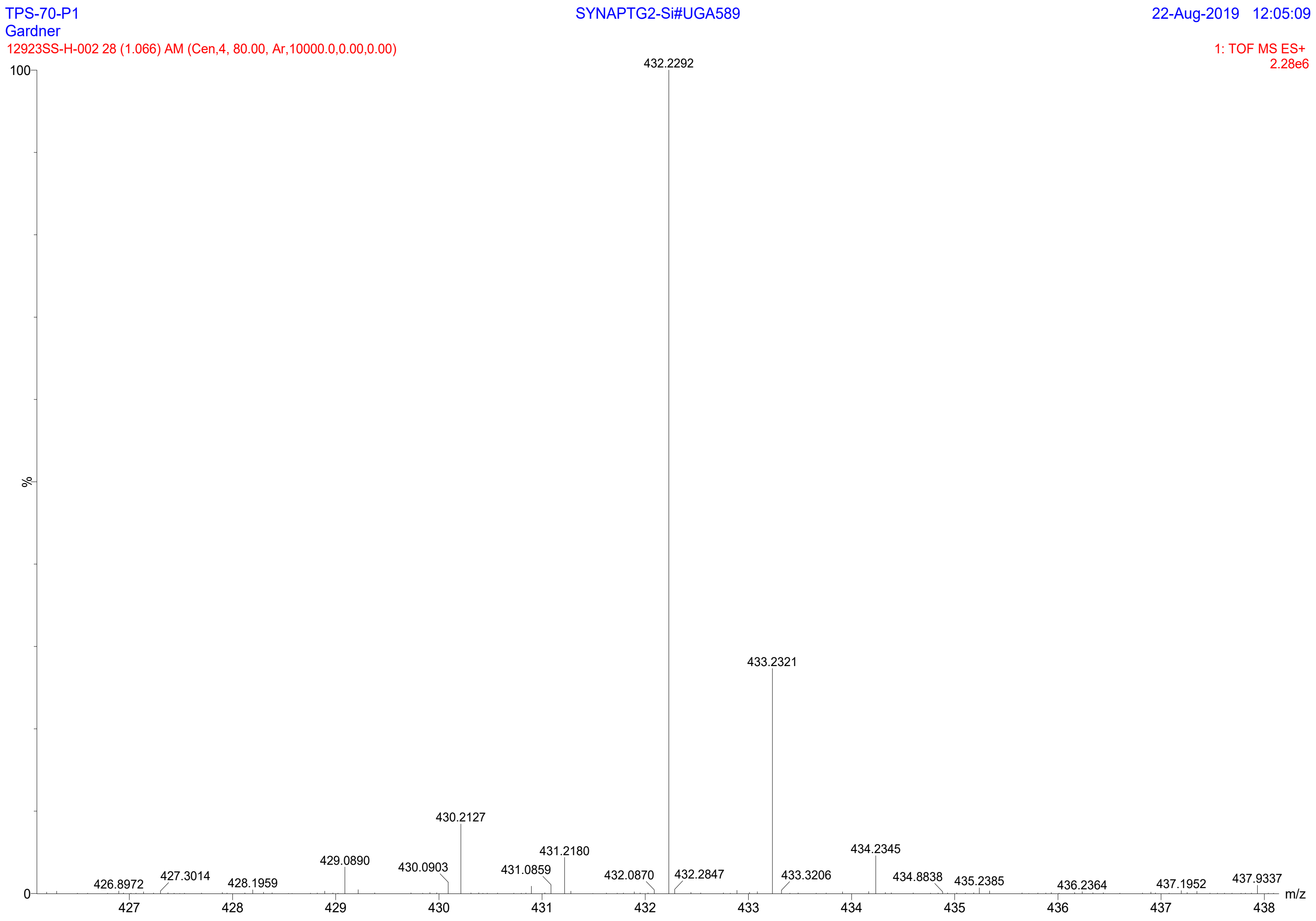

HRMS of **TPS-61-P1**

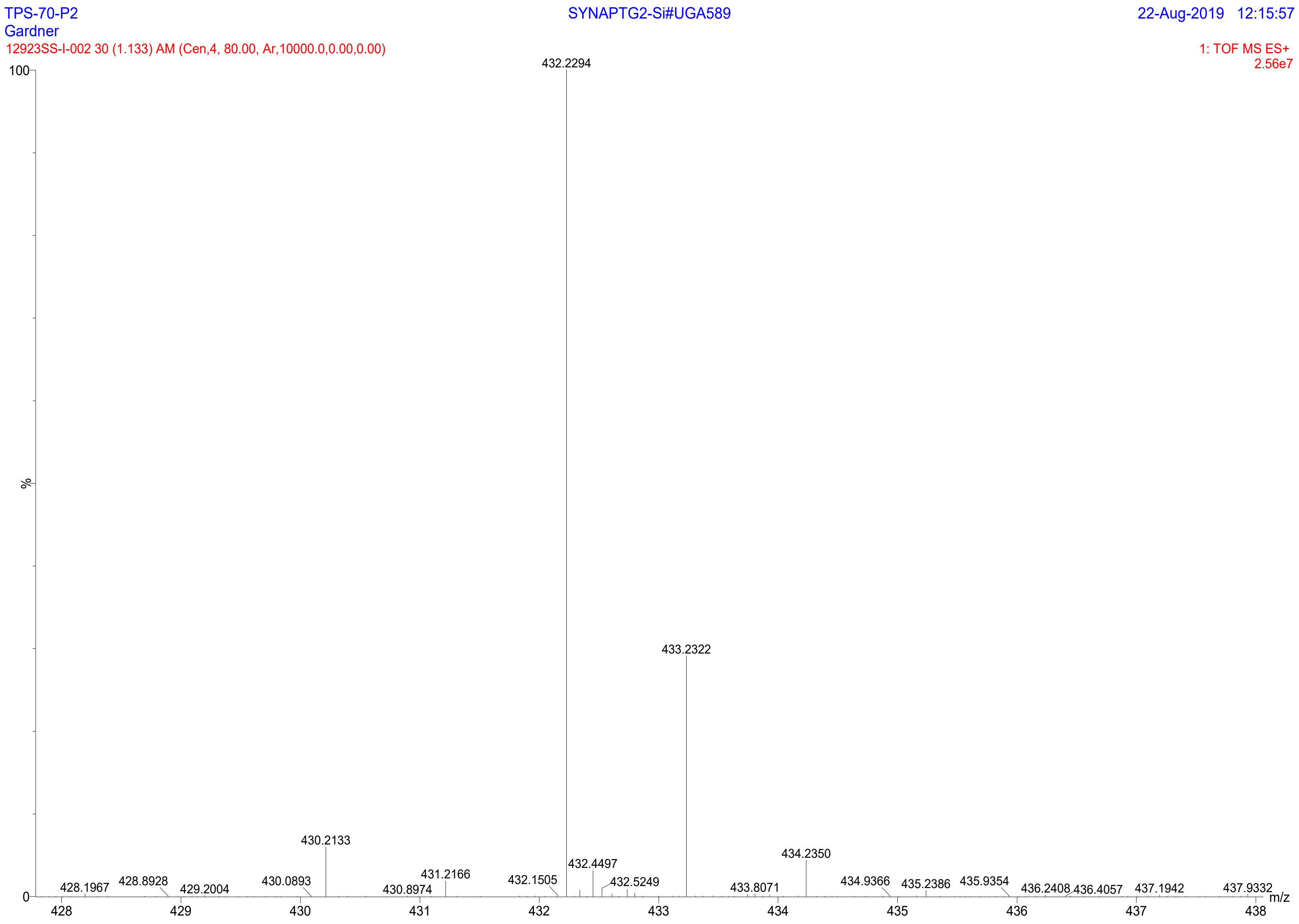

HRMS of **TPS-61-P2**

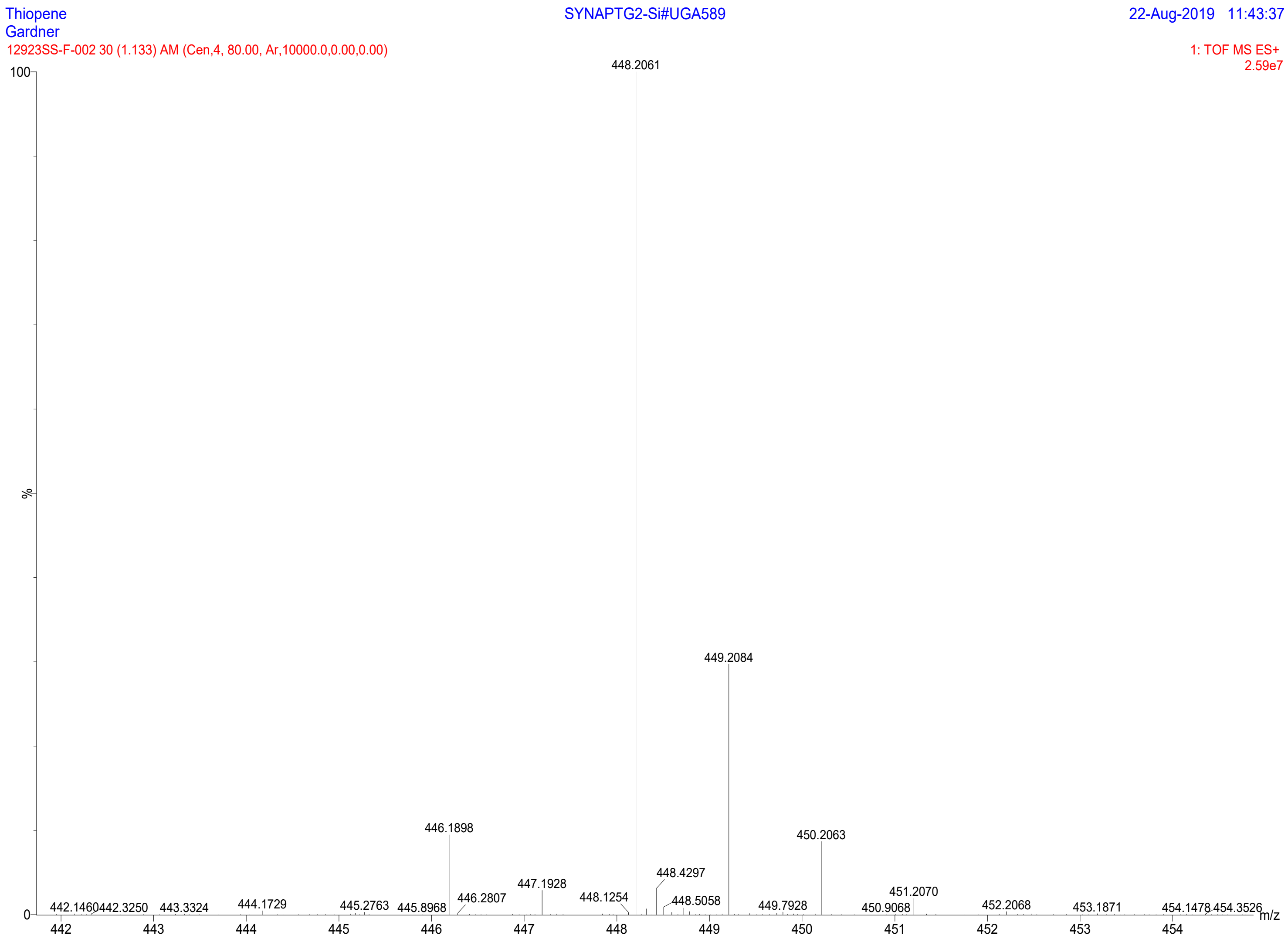

HRMS of **TPS-62**

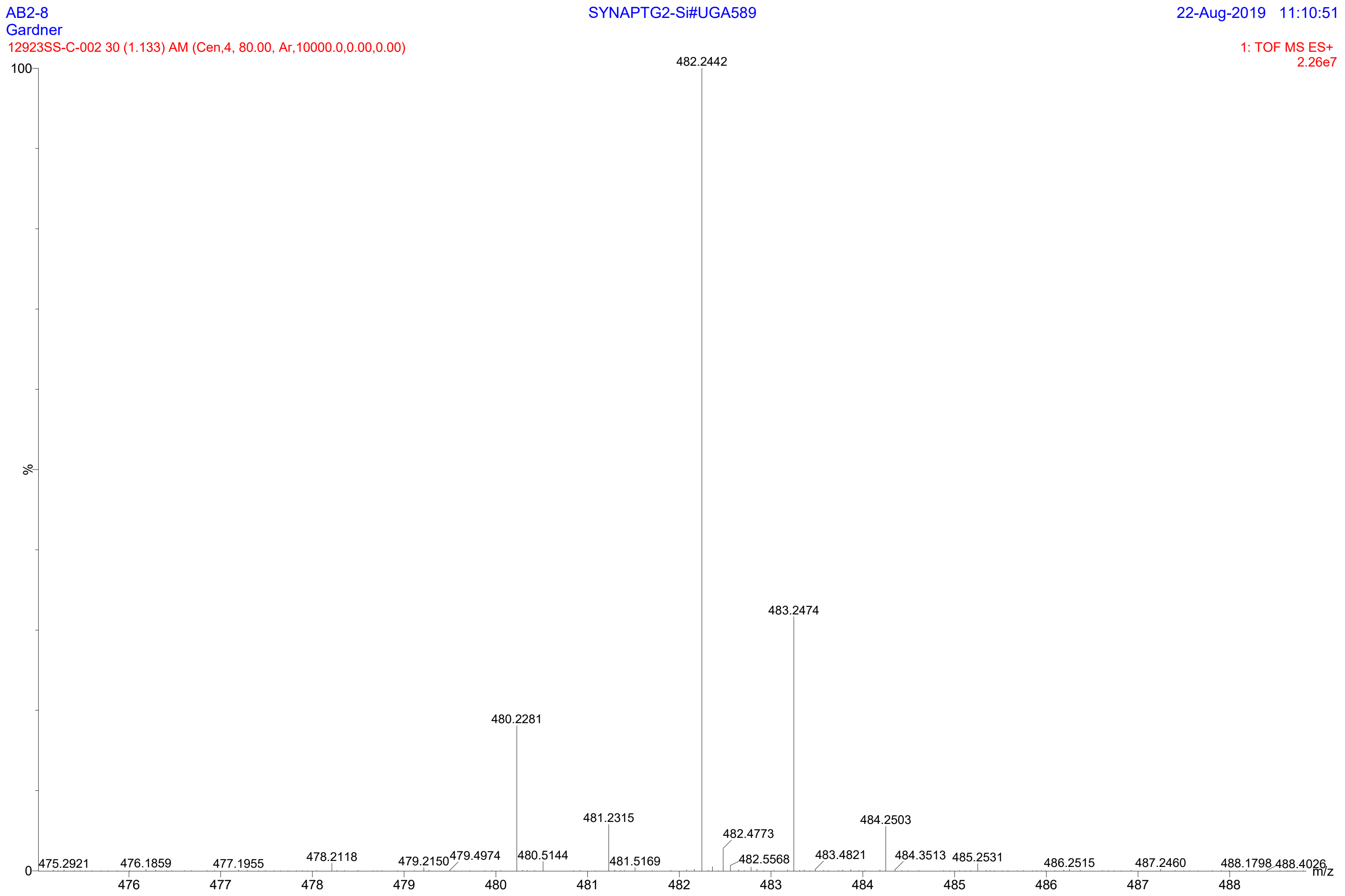

HRMS of **TPS-65**

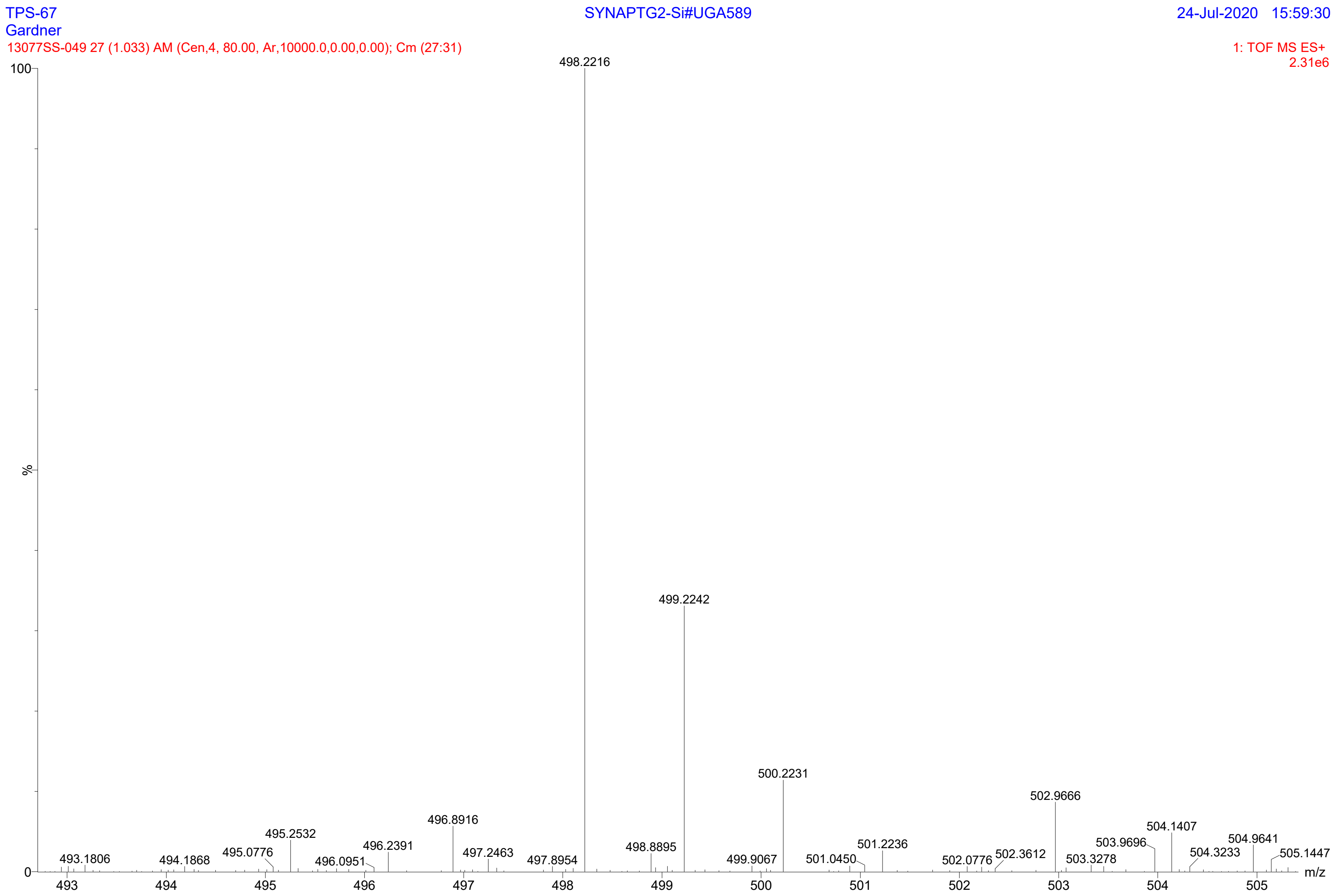

HRMS of **TPS-66**
